# Response strategies for COVID-19 epidemics in African settings: a mathematical modelling study

**DOI:** 10.1186/s12916-020-01789-2

**Published:** 2020-10-14

**Authors:** Kevin van Zandvoort, Christopher I. Jarvis, Carl A. B. Pearson, Nicholas G. Davies, CMMID COVID-19 working group, Emily S. Nightingale, James D. Munday, Amy Gimma, Alicia Rosello, Julian Villabona-Arenas, Sebastian Funk, Katherine E. Atkins, Charlie Diamond, Sophie R. Meakin, Simon R. Procter, Fiona Yueqian Sun, Akira Endo, Damien C. Tully, Eleanor M. Rees, Arminder K. Deol, Anna M. Foss, Petra Klepac, W. John Edmunds, Kiesha Prem, Jon C. Emery, Megan Auzenbergs, Sam Abbott, Samuel Clifford, Thibaut Jombart, Gwen Knight, Stéphane Hué, Quentin J. Leclerc, Kathleen O’Reilly, Billy J. Quilty, Rein M. G. J. Houben, Joel Hellewell, Nikos I. Bosse, Hamish P. Gibbs, Yang Liu, Graham Medley, Ruwan Ratnayake, Timothy W. Russell, Adam J. Kucharski, Mark Jit, Stefan Flasche, Rosalind M. Eggo, Francesco Checchi

**Affiliations:** 1 Department of Infectious Disease Epidemiology, Centre for Mathematical Modelling of Infectious Diseases, London School of Hygiene and Tropical Medicine, Keppel Street,, London, WC1E 7HT UK; 2 South African Centre for Epidemiological Modelling and Analysis, Stellenbosch University, Stellenbosch, Republic of South Africa; 3 Department of Infectious Disease Epidemiology, Health in Humanitarian Crises Centre, London School of Hygiene & Tropical Medicine, Keppel Street, London, WC1E 7HT UK

**Keywords:** COVID-19, SARS-CoV-2, Coronavirus, Africa, Low-income, Control, Response, Mathematical model

## Abstract

**Background:**

The health impact of COVID-19 may differ in African settings as compared to countries in Europe or China due to demographic, epidemiological, environmental and socio-economic factors. We evaluated strategies to reduce SARS-CoV-2 burden in African countries, so as to support decisions that balance minimising mortality, protecting health services and safeguarding livelihoods.

**Methods:**

We used a Susceptible-Exposed-Infectious-Recovered mathematical model, stratified by age, to predict the evolution of COVID-19 epidemics in three countries representing a range of age distributions in Africa (from oldest to youngest average age: Mauritius, Nigeria and Niger), under various effectiveness assumptions for combinations of different non-pharmaceutical interventions: self-isolation of symptomatic people, physical distancing and ‘shielding’ (physical isolation) of the high-risk population. We adapted model parameters to better represent uncertainty about what might be expected in African populations, in particular by shifting the distribution of severity risk towards younger ages and increasing the case-fatality ratio. We also present sensitivity analyses for key model parameters subject to uncertainty.

**Results:**

We predicted median symptomatic attack rates over the first 12 months of 23% (Niger) to 42% (Mauritius), peaking at 2–4 months, if epidemics were unmitigated. Self-isolation while symptomatic had a maximum impact of about 30% on reducing severe cases, while the impact of physical distancing varied widely depending on percent contact reduction and *R*_0_. The effect of shielding high-risk people, e.g. by rehousing them in physical isolation, was sensitive mainly to residual contact with low-risk people, and to a lesser extent to contact among shielded individuals. Mitigation strategies incorporating self-isolation of symptomatic individuals, moderate physical distancing and high uptake of shielding reduced predicted peak bed demand and mortality by around 50%. Lockdowns delayed epidemics by about 3 months. Estimates were sensitive to differences in age-specific social mixing patterns, as published in the literature, and assumptions on transmissibility, infectiousness of asymptomatic cases and risk of severe disease or death by age.

**Conclusions:**

In African settings, as elsewhere, current evidence suggests large COVID-19 epidemics are expected. However, African countries have fewer means to suppress transmission and manage cases. We found that self-isolation of symptomatic persons and general physical distancing are unlikely to avert very large epidemics, unless distancing takes the form of stringent lockdown measures. However, both interventions help to mitigate the epidemic. Shielding of high-risk individuals can reduce health service demand and, even more markedly, mortality if it features high uptake and low contact of shielded and unshielded people, with no increase in contact among shielded people. Strategies combining self-isolation, moderate physical distancing and shielding could achieve substantial reductions in mortality in African countries. Temporary lockdowns, where socioeconomically acceptable, can help gain crucial time for planning and expanding health service capacity.

## Background

The COVID-19 pandemic has not only led to increased mortality, but has also resulted in widespread socio-economic disruption and is severely testing affected countries’ health service capacity [1, 2]. However, to date, its effects have mainly been observed in countries with relatively well-resourced health systems and the financial means to support economies during ‘lock-down’ periods. In these and other low-income settings, two factors (younger age distributions and, potentially, warmer temperatures [3, 4]) may help to attenuate the pandemic’s severity. However, other factors may plausibly combine to worsen its impact: these include demography (larger household sizes and more intergenerational mixing within households), environmental conditions (overcrowded urban settlements, inadequate water and sanitation), pre-existing disease burden (higher prevalence of undiagnosed or unmanaged non-communicable diseases, tuberculosis and, if confirmed to be risk factors for COVID-19 severity, HIV and undernutrition) and, critically, a very low baseline of and access to hospitalisation capacity, particularly intensive and sub-intensive care [5–7]. In several African countries, armed conflict, food insecurity and resulting forced displacement further worsen societal resilience [8–13].

Options to manage COVID-19 in Africa may be limited. Sufficiently scaling up case management may simply be unfeasible for many countries as the requirements, particularly at the epidemic’s peak, may be many-fold greater than the baseline capacity. Even in scenarios where intense suppression measures are successfully implemented, it is plausible that the availability of beds, clinicians, oxygen and personal protective equipment would be critical limiting factors [14, 15]. Suppressing the epidemic through lock-down policies may delay transmission in the short-term, but their economic viability beyond a timeframe of weeks is questionable unless large economic rescue packages are made available by global financing actors and are concretely accessible to populations: indeed, lock-down measures and even less intense distancing restrictions could exacerbate poverty and undernutrition, compromise educational attainment and undo improvements in access to health interventions achieved over the past decades [16–18].

To help inform COVID-19 response strategies for African settings, we undertook a mathematical modelling study. We explored the possible effect on hospitalisation requirements and mortality of interventions considered to date in high-income settings, including self-isolation of symptomatic persons, general distancing (reduction of overall contacts) outside the household and more intensive lock-down measures. We also quantified the potential of an alternative option we refer to as ‘shielding’, whereby people at high risk of COVID-19 severe disease are specifically protected through a variety of community-led arrangements, such as neighbourhood-level house swaps, to create ‘green zones’ wherein high-risk residents are physically isolated for an extended period, but supported to live safely and with dignity: epidemiologically, this option seeks to reduce transmission within the high-risk groups that may otherwise contribute a large amount of hospitalisation and mortality.

## Methods

### Model structure

We adapted a previously developed discrete-time Susceptible-Exposed-Infectious-Removed (SEIR) compartmental model, stratified by age group and disease status (asymptomatic, pre-symptomatic, and symptomatic) [19]. Detail is provided in Additional file 1.

In brief, the model progresses a population through time based on assumed age-dependent contact of susceptible with infectious individuals. After their infectious period, all individuals are assumed to be immune until the end of the simulation (Removed compartment). The model is stratified into 16 age groups, with people under 75 years stratified into 5-year age-bands and one additional stratum for ≥ 75 years. The model population is closed, with no births or ageing, and deaths remain in the Removed compartment.

### Transmissibility assumptions

We adopted epidemiological parameter values (serial interval, infectiousness by symptom stage, and incubation, infectiousness and symptomatic periods) used by Davies et al. [20, 21] (see Additional file 1). We assumed an age-dependent probability of developing clinical symptoms [20]. Asymptomatic individuals were assumed to be half as infectious as symptomatic individuals, but we present below and in Additional file 1 a sensitivity analysis of this assumption. Clinical progression of symptomatic cases to severe disease is assumed not to affect their infectiousness.

To represent the full range of age structures in Africa, we ranked countries according to their mean age, and selected countries with the youngest (Niger), oldest (Mauritius) and median (Nigeria) mean age as case studies. Key country-specific data inputs were age-specific population sizes, sourced from United Nations World Population Prospects estimates [22] and age- and setting-specific social contact matrices. As no empirical data on age-specific social mixing patterns were available for these three countries, we used previously published synthetic contact matrices, namely projections of a European multi-country contact pattern study adjusted to individual countries based on national demographic and socio-economic characteristics [23]. As an alternative (summarised in the main text and presented more extensively in Additional file 1), we sampled randomly from three empirical contact matrices from previously published studies of populations in semi-urban to rural communities in Kilifi, Kenya, rural Mbarara district, Uganda and Bulawayo city in Zimbabwe [24–26], with equal probability of sampling for each of the three matrices. All contact data were stratified according to whether the contact was within or outside the household.

To account for uncertainty regarding the transmissibility of SARS-CoV-2 in Africa, we implemented the model stochastically. We sampled values for the basic reproduction number *R*_0_ from estimates from country-specific effective reproduction numbers *R*_*t*_ [27] for early March, prior to implementation of lockdowns [28], where estimates were above 1. These *R*_*t*_ values are estimated from a time series of reported cases, accounting for delays between infection and case onset. However, these estimates do not account for potential underreporting of cases, nor do they adjust for some of the control measures (e.g. case finding) already implemented before lockdowns (WHO database ref). Therefore, we also sampled *R*_0_ estimates from a pooled distribution of early *R*_0_ estimates and present results for both *R*_*t*_ and *R*_0_ scenarios. Early mean estimates for *R*_*t*_ were 1.8 (1.2–2.5) for Niger and 1.6 (1.1–2.3) for Nigeria. Due to a low number of reported cases, no reliable estimate of *R*_*t*_ was available for Mauritius, and we instead sampled from all early *R*_*t*_ estimates from all available African countries, resulting in a mean *R*_*t*_ estimate of 1.6 (1.1–3.3). Mean estimated *R*_0_ using the global distribution was 2.6 (1.6–3.6). Preliminary sensitivity analyses showed that variability in model output was mainly affected by variability in *R*_0_. Due to the larger variability in the *R*_0_ estimates compared to the *R*_*t*_ estimates, in each scenario, we used 400 model runs using the former and 200 model runs using the latter. We implemented assumed *R*_0_ estimates by scaling the probability of transmission per contact with an infectious person in accordance with the ratio between the target *R*_0_ and the dominant eigenvalue of the Next-Generation Matrix (Additional file 1). We explore the effect of different *R*_0_ values in Additional file 1.

### Case severity assumptions

In high-income countries, the severity of SARS-CoV-2 infections has been shown to increase with age and prevalence of various comorbidities [29–31]. In practice, individual comorbidities co-occur (e.g. diabetes and hypertension), and co- and multimorbidity increase with age [32]. To simplify assumptions, we took age as a single predictor of severity, applying current evidence on its association with risk of symptomatic disease, severe disease (i.e. requiring hospitalisation), critical disease (need for intensive care) and death.

However, in African and low-income countries, an average person’s underlying vulnerability may correspond to that of an individual with greater chronological age in a high-income setting due to life-course effects including malnutrition, infections and often unmanaged non-communicable diseases. It is not yet known how this might affect COVID-19 severity, although Global Burden of Disease estimates show strong associations between income level and the severity of other respiratory infections, particularly in younger age groups [33]. To account for this, we shifted age-specific severity risks (probability of becoming a severe case) towards younger age by 10 years. We also present results for a 0- and 5-year shift. To explore the effect of increased vulnerability and lack of access to healthcare, we also multiplied current estimates of age-specific case-fatality ratios (CFR; from China and the Diamond Princess cruise ship outbreak [34]) among symptomatic cases by a factor of 1.5 (used for our main analysis, with values of 1.0 and 2.0 explored in sensitivity analysis; see Additional file 1). We did not make assumptions about the proportion of cases that would receive appropriate treatment: the CFR multiplier factor attempts to capture worse prognosis under limited or no treatment.

### Response interventions

The range of response interventions explored, alone or in combination, are outlined in Table 1, and more information on their model implementation is found in Additional file 1. We assumed these would be applied at the country level, and initiated once daily incidence crosses a threshold of 1 infection per 10,000 people. Once initiated, interventions would remain in place over 12 months (in practice, we expect that interventions would be lifted sooner if the epidemic is demonstrably over).

**Table 1:** Summary of response interventions explored in the study

Intervention	Description	Model implementation	Range explored
Self-isolation of symptomatic people	People with symptoms of possible COVID-19 isolate themselves in their home until symptom-free.	Relative reduction in all social contacts among symptomatic people only, during the period over which they are symptomatic.	0–100% relative reduction in transmission.
General physical distancing (including reduction of probability of transmission per contact)	Behaviour change, promotion of handwashing, varying degrees of curtailment of transport, social and work gatherings.	Relative reduction in contacts outside of the household.	0–100% relative reduction in transmission (we assumed that ‘lockdown’ measures would correspond to an 80% reduction) [35].
Shielding of high-risk groups	Communities identify people who meet high-risk criteria for poor COVID-19 clinical outcomes and resettle them in a variety of shielded arrangements (either individual, e.g. a dedicated room within a house, or groups of various sizes in vacated / swapped houses, huts or other shelters). Contact is thereafter limited. Within these shielded ‘green zones’, residents also adopt distancing and hygiene (handwashing, face coverings if locally appropriate) measures if they are living together.	A proportion of people aged ≥ 60 years old is ‘shielded’. Contact between shielded people and unshielded people both inside and outside the household is reduced. Contact among shielded people varies depending on the arrangement chosen.	60–100% of high-risk people are shielded. 60–100% relative reduction in contact with non-shielded people. 0–400% relative change in social contact among shielded people, compared to contacts among people eligible for shielding before they were shielded (0% represents individual shielding arrangements; 400% represents what might happen if people are grouped within overcrowded shielded housing).

Self-isolation was implemented as a reduction in transmissibility of infected people during their symptomatic period, equivalent to a reduction in their contacts. We did not account for additional quarantine of other members in the same household.

General physical distancing was implemented as a reduction in all contacts outside the household. We assumed no change in transmission within the household.

Shielding was implemented by stratifying the population into one shielded and one unshielded compartment. In the presence of shielding, mixing between the shielded and unshielded population would reduce by some degree, while mixing within the shielded population may remain the same as the pre-shielding baseline (i.e. how much contact people within the group eligible for shielding had among themselves before they were shielded), decrease (to zero if people are shielded individually) or increase relative to this baseline if shielded people are resettled in overcrowded housing. While in the intervention’s practical application people should be shielded on the basis of age and/or known comorbidities, for this model we assumed more simplistically that varying proportions of the population aged 60 years and above would be shielded.

### Analysis outcomes

We compared outcomes under different intervention values and strategies (combinations of interventions) for each unique combination of sampled *R*_0_ and model seeds. We present median, 50% and 95% quantiles of differences in outcomes across all combinations, and show results stratified by *R*_0_ in Additional file 1.

For the impact of individual interventions, we considered severe cases as our primary outcome. For the impact of different strategies, we observed the total number of symptomatic cases, severe cases (those who require hospitalisation), critical cases (those who require intensive care, ICU), and deaths, and present the expected time until peak of the epidemic, including peak bed demand. We mainly show estimates for the first 12 months after introduction of the first case, as the evolution of the epidemic beyond this period is subject to considerable unknowns (e.g. availability of vaccines or therapeutics; virus mutation, persistence of natural immunity). However, we also observe the probability of a second peak over the subsequent year.

## Results

### Epidemic trajectories in the absence of control

**Fig. 1:**
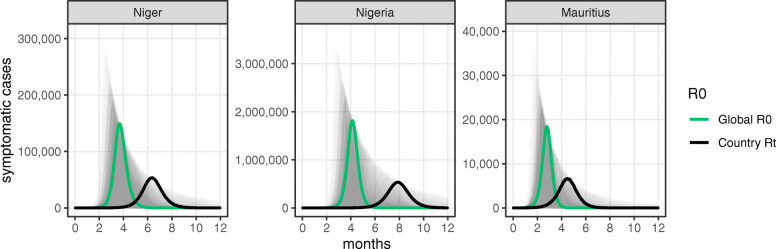
Projected incidence of symptomatic COVID-19 cases over time for simulations of an unmitigated epidemic, by country. The green line shows the run that was closest to the median total number of cases across all model runs using global *R*_0_ estimates. The black line shows the run that was closest to the median total number of cases across all model runs using country-specific *R*_*t*_ estimates. Grey lines show individual stochastic model runs, where *R*_0_ in each run was sampled from the respective distribution

Using country specific *R*_*t*_ estimates, simulations of an unmitigated epidemic in Niger resulted in a median of 3.3 million clinical cases during the first 12 months following introduction of the first case, 35.6 million in Nigeria and 380,000 in Mauritius (Table 2), with the most probable epidemic peaks after 6, 8 and 5 months respectively (Fig. 1). We estimate some 20,000 deaths due to COVID-19 in Niger, 270,000 in Nigeria and 8000 in Mauritius would occur over the same period, not accounting for indirect excess mortality due to health service or socio-economic disruptions. The impact of assumed *R*_0_ is large. Global *R*_0_ estimates yield considerably higher attack rates and caseloads in Niger and Mauritius (Table 2). Larger epidemics that peak early occur in scenarios where *R*_0_ is high (median total severe cases when *R*_0_ ≥ 3 are estimated as 171,000, 2,500,000, and 59,000 in Niger, Nigeria, and Mauritius), whereas epidemics with lower *R*_0_ will have a lower total and peak epidemic size (median total severe cases when 2 ≤ *R*_0_ < 3 are estimated as 108,000, 1,800,000, and 46,000 in Niger, Nigeria, and Mauritius) and will peak later (Additional file 1, Figure S11).

**Table 2:** Projected impact of unmitigated COVID-19 epidemics during the first 12 months following introduction of cases, by country. All values represent the median and 95% lower and upper quantiles from all model runs in each scenario. The symptomatic attack rate is calculated as the total number of symptomatic cases divided by the population. We show the months until the epidemic peak (defined as the day with the highest number of new cases) and present the peak daily number of deaths and hospital bed demand

Key outcome	Niger	Nigeria	Mauritius
Population size	24,100,000	202,900,000	1,300,000
Population aged ≥ 60 years	4%	5%	18%
Projections using country ***R***_***t***_ estimates			
Symptomatic cases	3,270,000 (30,000 to 4,980,000)	35,570,000 (5000 to 53,270,000)	380,000 (5000 to 610,000)
Symptomatic attack rate	13.6% (0.1 to 20.7)	17.5% (0 to 26.3)	29.1% (0.4 to 46.6)
Severe, non-critical cases	60,000 (300 to 110,000)	1,020,000 (100 to 1,740,000)	30,000 (400 to 60,000)
Severe, critical cases	30,000 (100 to 50,000)	440,000 (40 to 750,000)	10,000 (100 to 30,000)
Deaths	20,000 (70 to 30,000)	270,000 (30 to 460,000)	8000 (90 to 20,000)
Deaths per 1000 person-years	0.7 (0 to 1.3)	1.3 (0 to 2.3)	6.5 (0.1 to 12.8)
Epidemic peak (month)	6 (4 to 12)	8 (5 to 12)	5 (2 to 12)
Peak deaths	300 (4 to 700)	4000 (2 to 10,000)	200 (3 to 700)
Peak demand for non-ICU beds	8000 (40 to 20,000)	120,000 (10 to 330,000)	4000 (30 to 20,000)
Peak demand for ICU beds	4000 (20 to 10,000)	60,000 (7 to 180,000)	2000 (20 to 10,000)
Projections using global ***R***_**0**_ estimates			
Symptomatic cases	5,480,000 (2,980,000 to 6,840,000)	60,200,000 (35,920,000 to 69,730,000)	540,000 (360,000 to 610,000)
Symptomatic attack rate	22.7% (12.3 to 28.4)	29.7% (17.7 to 34.4)	41.7% (27.7 to 46.6)
Severe, non-critical cases	130,000 (50,000 to 190,000)	2,090,000 (1,030,000 to 2,670,000)	50,000 (30,000 to 60,000)
Severe, critical cases	60,000 (20,000 to 80,000)	890,000 (440,000 to 1,150,000)	20,000 (10,000 to 30,000)
Deaths	30,000 (10,000 to 50,000)	560,000 (270,000 to 710,000)	10,000 (8000 to 20,000)
Deaths per 1000 person-years	1.4 (0.6 to 2.1)	2.7 (1.4 to 3.5)	10.6 (6.1 to 12.9)
Epidemic peak (month)	4 (3 to 7)	4 (3 to 8)	3 (2 to 5)
Peak deaths	900 (200 to 2000)	20,000 (4000 to 30,000)	400 (100 to 700)
Peak demand for non-ICU beds	30,000 (6000 to 50,000)	480,000 (120,000 to 800,000)	10,000 (4000 to 20,000)
Peak demand for ICU beds	10,000 (3000 to 30,000)	250,000 (70,000 to 420,000)	7000 (2000 to 10,000)

### Effect of individual interventions

#### Self-isolation of symptomatic individuals

**Fig. 2:**
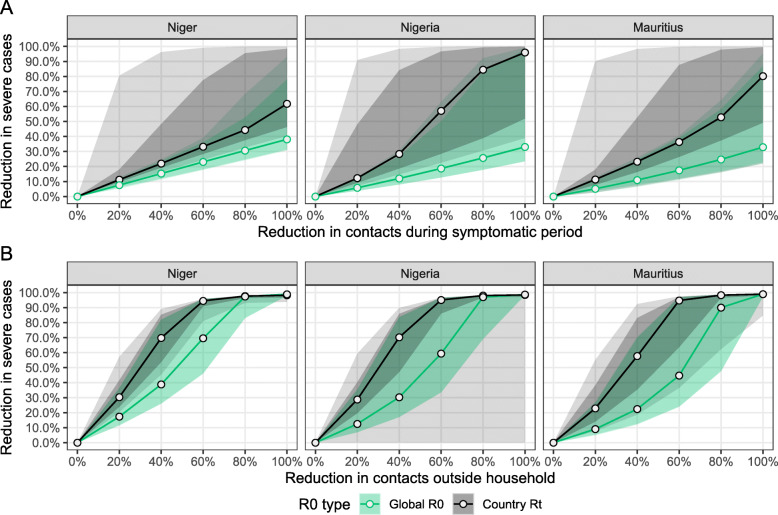
Estimated reduction in severe cases following **a** self-isolation of symptomatic individuals and **b** population-wide physical distancing, using synthetic contact matrices. Medians (circles), 75% (lighter shaded areas) and 50% (darker shaded area) quantiles for the percentage reduction in severe cases during the first 12 months of the epidemic for different levels of compliance, for each country, across all model runs in each scenario. Quantiles are calculated across all simulations representing different stochastic runs and using different *R*_0_ values in each run, drawn from a distribution of global *R*_0_ estimates or from a distribution of country-specific *R*_*t*_ estimates. Estimates for reductions where no point is available are interpolated

We estimate a reduction in severe cases during the first 12 months of the epidemic if symptomatic cases self-isolate throughout this period with varying levels of compliance (Fig. 2a). Increasing compliance has a nearly linear relationship with the incidence of severe cases when *R*_0_ values are high, but the maximum median impact is a 40% reduction (under an extreme scenario of 100% reduction in transmissibility of all cases while symptomatic) when global *R*_0_ estimates are assumed, with little change if empirical contact matrices are used instead (Figure S5). However, impact of self-isolation could be much greater when *R*_0_ estimates are lower, as reflected by the estimates under country-specific *R*_*t*_ estimates. Although the probability of being a symptomatic case is modelled as age-dependent, the impact of self-isolation was similar in all countries despite their different demographic distributions. Uncertainty intervals for Nigeria and Mauritius were wider, but mainly reflected differences in *R*_0_ between simulations (Figure S11). In simulations where a low *R*_0_ was used, there was potential for a larger reduction in severe cases. The impact of *R*_0_ variability is smaller in Niger, where a higher proportion of transmission is due to asymptomatic infections due to its lower average age and the age-dependency of becoming a symptomatic case, as reflected by the symptomatic attack rates in Table 2. As a result, impact of self-isolation is lower in Niger under country-specific *R*_*t*_ estimates.

#### Population-wide physical distancing

Figure 2b shows the estimated impact of population-wide (i.e. not targeting any group) physical distancing, whereby all individuals reduce their contacts outside of the household to a certain degree, while contacts within the household remain unchanged. Across all three countries, reducing all contacts outside the household by an extreme of 100%, if sustained over the entire 12 months’ period, could result in a median reduction in severe cases close to 100%, with even greater impact at lower levels of physical distancing when *R*_0_ values are lower. However, this would largely delay rather than prevent severe cases, as insufficient levels of herd immunity would develop, leading to a second wave of cases following the relaxation of measures. Depending on the actual *R*_0_ of Covid-19, relatively high reductions in contacts may be needed for large impacts. Patterns were largely consistent across countries. Stratified results by *R*_0_ are shown in Figure S11, showing higher potential for impact in settings with lower *R*_0_. For instance, when reducing 40% of contacts outside of the household, reduction in severe cases in the first 12 months of the epidemic could be as low as 10% in scenarios with an *R*_0_ above 3, while it could be as high as 70% in scenarios with an *R*_0_ below 2, e.g. under country-specific *R*_*t*_ estimates. Results using empirical matrices are slightly less favourable (Figure S5).

#### Shielding of high-risk individuals

**Fig. 3:**
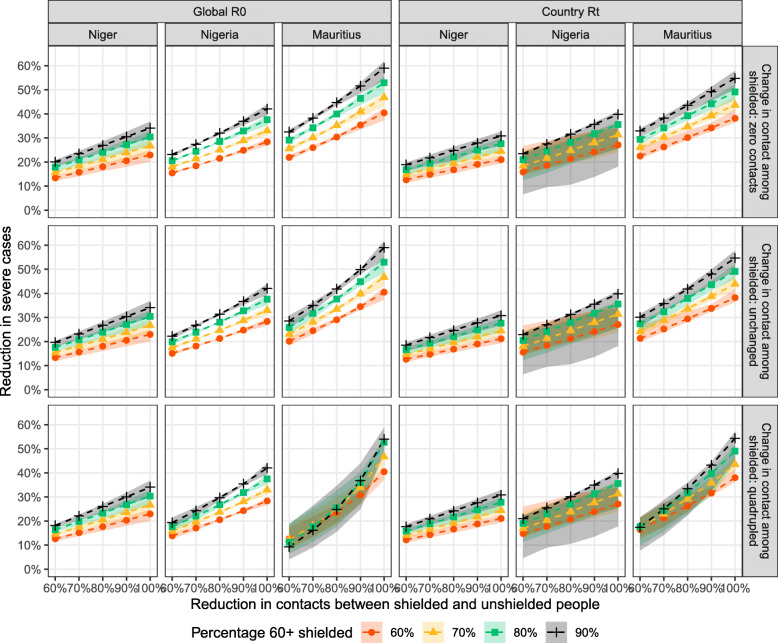
Estimated reduction in severe cases when shielding high-risk individuals, by country, using synthetic contact matrices. Medians (dashed lines) and 75% quantiles (shaded areas) of the percentage reduction in severe cases during the first 12 months of the epidemic for different levels of reduction in contacts between shielded and unshielded people (*x* axis), different level of contacts among shielded people (facet rows), and for different percentages of people ≥ 60 years old shielded (see legend), for each country, across all model runs in each scenario

Shielding of high-risk individuals aims to reduce the number of severe cases among high-risk groups, and thereby in the overall population, while having a smaller effect on transmission in the population and thereby on the total number of cases. Figure 3 shows the reduction in the number of severe cases for different reductions in contacts between shielded and unshielded individuals, percentages of individuals shielded and changes in contact intensity within the shielded group, relative to baseline.

Across all countries, reductions in severe cases increased with the percentage ≥ 60 years old shielded, but the reduction in contacts between shielded and unshielded individuals was more influential, with ≥ 60% reduction in contacts required to achieve ≥ 10% reduction in severe cases. The degree of contact among shielded individuals appeared to be of lesser importance for Niger and Nigeria, with similar effect sizes regardless of whether the shielded individuals reduce their contact with one another to zero, remain at baseline or quadruple it. This pattern does not hold for Mauritius, where a marked drop in the effect is seen when contact among shielded individuals quadruples, even more so when empirical contact matrices are used (Figure S6): this is reflective of Mauritius having a larger elderly population that contributes more to the overall proportion of severe cases in the population and can more easily sustain transmission within itself.

As shielding does not significantly affect transmission dynamics, estimates are similar across scenarios with low and high *R*_0_ (Figure S12), which is why estimates do not differ much between scenarios using global *R*_0_ estimates or country-specific *R*_*t*_ estimates. However, prediction intervals are wider in Mauritius, where a high proportion of the total population is shielded (8–14%). In addition, prediction intervals are wider using country *R*_*t*_ estimates, as the stochastic effects of the model have a larger effect on the results when assumed *R*_0_ is lower and total epidemic sizes are smaller.

### Impact of potential control strategies

#### Without lockdowns

We explored the impact of five different strategies and compared their impact to the unmitigated epidemic over the first 12 months after introduction of the first case. We assumed that self-isolation of symptomatic individuals, featuring 50% reduction in their infectiousness, would be part of any strategy. We then added (i) 20% or (ii) 50% reduction in contacts outside the household through physical distancing; (iii) shielding of 80% of individuals aged 60 and older, with a reduction of 80% in contacts between the shielded and unshielded population and no change in contacts within the shielded population; (iv) a combination of shielding and 20% physical distancing; and (v) shielding with 50% physical distancing.

**Fig. 4:**
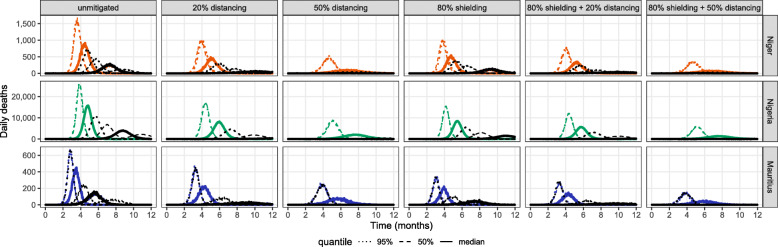
Estimated daily number of deaths during the first 18 months of the epidemic, under different strategies. Black lines show results using country *R*_*t*_ estimates, while coloured lines show results using global *R*_0_ estimates. Thick solid lines show the run which was closest to the median total number of deaths after 12 months across all model runs. Dashed lines are runs closest to the lower and upper 95% quantiles, while dotted lines are runs closest to the lower and upper 50% quantiles of total number of deaths, calculated over 600 model runs. Except for the unmitigated scenario, all scenarios assume 50% self-isolation during the symptomatic period of all symptomatic cases throughout the entire course of the epidemic. Other interventions start when daily incidence of symptomatic cases reaches 1 case per 10,000 people. Distancing strategies assume 20% or 50% reduction in all contacts outside of the household. Shielding strategies assume shielding of 80% of the population aged ≥ 60 years, irrespective of underlying comorbidities, with an 80% reduction in contacts between the shielded and unshielded population, and no change in contacts within the shielded population. Estimates for bed demand over time are given in Figure S2

Figure 4 shows the evolution of deaths depending on the strategy chosen. Evolution of bed demand under each scenario is given in Figure S2. Table 3 shows the corresponding attack rate, total number of cases, severe cases, and critical cases, time of epidemic peak and peak bed demand for severe and critical cases. All strategies yielded substantial but partial reductions in key health outcomes. Under all strategies, we estimate a high bed capacity needed in the three countries modelled.

**Table 3:** Key outcomes during the first 12 months of the epidemic under different strategies, by country, using synthetic contact matrices. Symptomatic attack rate is calculated as number of symptomatic cases over the total population size. Death rate is per 1000 person-years. We show estimates under different modelled strategies: (i) physical distancing with 20% reduction and (ii) 50% reduction in contacts outside of the household; (iii) shielding of 80% of the population aged ≥ 60 years, with an 80% reduction in contacts between the shielded and unshielded population and no change in contacts within the shielded population; and combined shielding and physical distancing with (iv) 20% and (v) 50% reductions in contacts outside of the household. All strategies also include self-isolation of symptomatic cases, reducing their infectiousness by 50%

Key outcome	Unmitigated	20% distancing	50% distancing	80% shielding	80% shielding + 20% distancing	80% shielding + 50% distancing
Using country *R*_*t*_ estimates						
Niger						
Symptomatic cases	3,270,000 (30,000 to 4,980,000)	1,420,000 (200 to 3,410,000)	30,000 (200 to 1,240,000)	2,520,000 (200 to 4,190,000)	1,350,000 (200 to 3,340,000)	30,000 (200 to 1,220,000)
Severe, non-critical cases	60,000 (300 to 110,000)	20,000 (7 to 70,000)	600 (7 to 20,000)	40,000 (7 to 70,000)	20,000 (7 to 50,000)	500 (7 to 20,000)
Severe, critical cases	30,000 (100 to 50,000)	10,000 (4 to 30,000)	200 (4 to 10,000)	20,000 (5 to 30,000)	7000 (5 to 20,000)	200 (5 to 8000)
Deaths	20,000 (70 to 30,000)	6000 (2 to 20,000)	100 (2 to 6000)	9000 (2 to 20,000)	4000 (2 to 10,000)	100 (2 to 5000)
Symptomatic attack rate	13.6% (0.1 to 20.7)	5.9% (0 to 14.2)	0.1% (0 to 5.1)	10.4% (0 to 17.4)	5.6% (0 to 13.8)	0.1% (0 to 5)
Deaths per 1000 person-years	0.7 (0 to 1.3)	0.2 (0 to 0.8)	0 (0 to 0.3)	0.4 (0 to 0.8)	0.2 (0 to 0.6)	0 (0 to 0.2)
Epidemic peak (months)	6 (4 to 12)	8 (0 to 12)	5 (0 to 10)	7 (0 to 12)	8 (0 to 12)	5 (0 to 11)
Peak deaths	300 (4 to 700)	70 (1 to 300)	5 (1 to 60)	100 (1 to 400)	50 (1 to 300)	4 (1 to 40)
Peak non-ICU beds needed	8000 (40 to 20,000)	2000 (4 to 9000)	60 (4 to 1000)	4000 (5 to 10,000)	1000 (4 to 7000)	60 (4 to 900)
Peak ICU beds needed	4000 (20 to 10,000)	900 (2 to 5000)	30 (2 to 700)	2000 (3 to 6000)	700 (3 to 4000)	30 (2 to 500)
Nigeria						
Symptomatic cases	35,570,000 (5000 to 53,270,000)	4,600,000 (300 to 36,610,000)	110,000 (300 to 8,480,000)	24,050,000 (200 to 44,300,000)	4,010,000 (200 to 35,170,000)	120,000 (200 to 7,490,000)
Severe, non-critical cases	1,020,000 (100 to 1,740,000)	90,000 (9 to 1,110,000)	3000 (9 to 230,000)	450,000 (7 to 1,020,000)	60,000 (7 to 780,000)	2000 (7 to 140,000)
Severe, critical cases	440,000 (40 to 750,000)	40,000 (4 to 470,000)	1000 (4 to 100,000)	190,000 (3 to 440,000)	20,000 (3 to 340,000)	1000 (3 to 60,000)
Deaths	270,000 (30 to 460,000)	20,000 (2 to 290,000)	800 (2 to 60,000)	110,000 (3 to 270,000)	10,000 (3 to 210,000)	700 (3 to 40,000)
Symptomatic attack rate	17.5% (0 to 26.3)	2.3% (0 to 18)	0.1% (0 to 4.2)	11.9% (0 to 21.8)	2% (0 to 17.3)	0.1% (0 to 3.7)
Deaths per 1000 person-years	1.3 (0 to 2.3)	0.1 (0 to 1.5)	0 (0 to 0.3)	0.6 (0 to 1.3)	0.1 (0 to 1)	0 (0 to 0.2)
Epidemic peak (months)	8 (5 to 12)	11 (0 to 12)	6 (0 to 12)	9 (0 to 12)	11 (0 to 12)	6 (0 to 12)
Peak deaths	4000 (2 to 10,000)	400 (1 to 5000)	20 (1 to 400)	2000 (1 to 6000)	300 (1 to 3000)	10 (1 to 300)
Peak non-ICU beds needed	120,000 (10 to 330,000)	10,000 (2 to 140,000)	300 (2 to 10,000)	50,000 (3 to 170,000)	8000 (3 to 100,000)	300 (3 to 8000)
Peak ICU beds needed	60,000 (7 to 180,000)	6000 (1 to 70,000)	200 (1 to 6000)	20,000 (2 to 90,000)	4000 (2 to 50,000)	200 (2 to 4000)
Mauritius						
Symptomatic cases	380,000 (5000 to 610,000)	160,000 (200 to 550,000)	3000 (200 to 460,000)	250,000 (100 to 500,000)	140,000 (100 to 480,000)	3000 (100 to 400,000)
Severe, non-critical cases	30,000 (400 to 60,000)	10,000 (10 to 50,000)	200 (10 to 40,000)	10,000 (9 to 40,000)	7000 (9 to 30,000)	200 (9 to 30,000)
Severe, critical cases	10,000 (100 to 30,000)	5000 (6 to 20,000)	100 (6 to 20,000)	6000 (3 to 20,000)	3000 (3 to 10,000)	70 (3 to 10,000)
Deaths	8000 (90 to 20,000)	3000 (3 to 10,000)	70 (3 to 10,000)	4000 (1 to 9000)	2000 (1 to 9000)	40 (1 to 7000)
Symptomatic attack rate	29.1% (0.4 to 46.6)	12.4% (0 to 42)	0.2% (0 to 35.2)	19.1% (0 to 38.8)	10.5% (0 to 36.7)	0.2% (0 to 30.5)
Deaths per 1000 person-years	6.5 (0.1 to 12.8)	2.5 (0 to 10.9)	0.1 (0 to 8.9)	2.8 (0 to 7.2)	1.4 (0 to 6.8)	0 (0 to 5.5)
Epidemic peak (months)	5 (2 to 12)	5 (0 to 12)	3 (0 to 10)	6 (0 to 12)	5 (0 to 12)	3 (0 to 9)
Peak deaths	200 (3 to 700)	30 (1 to 500)	3 (1 to 300)	60 (1 to 400)	20 (1 to 300)	3 (1 to 200)
Peak non-ICU beds needed	4000 (30 to 20,000)	700 (4 to 10,000)	30 (4 to 8000)	1000 (3 to 10,000)	400 (3 to 8000)	20 (3 to 5000)
Peak ICU beds needed	2000 (20 to 10,000)	400 (2 to 7000)	20 (2 to 4000)	700 (2 to 5000)	200 (2 to 4000)	10 (2 to 2000)
Using global *R*_0_ estimates						
Niger						
Symptomatic cases	5,480,000 (2,980,000 to 6,840,000)	3,970,000 (660,000 to 5,600,000)	1,980,000 (10,000 to 4,030,000)	4,680,000 (2,220,000 to 6,080,000)	3,870,000 (710,000 to 5,440,000)	1,920,000 (10,000 to 3,910,000)
Severe, non-critical cases	130,000 (50,000 to 190,000)	80,000 (9000 to 140,000)	40,000 (200 to 100,000)	80,000 (30,000 to 120,000)	60,000 (8000 to 110,000)	30,000 (200 to 70,000)
Severe, critical cases	60,000 (20,000 to 80,000)	40,000 (4000 to 60,000)	20,000 (70 to 40,000)	30,000 (10,000 to 50,000)	30,000 (3000 to 50,000)	10,000 (70 to 30,000)
Deaths	30,000 (10,000 to 50,000)	20,000 (2000 to 40,000)	10,000 (40 to 30,000)	20,000 (8000 to 30,000)	20,000 (2000 to 30,000)	8000 (50 to 20,000)
Symptomatic attack rate	22.7% (12.3 to 28.4)	16.5% (2.7 to 23.2)	8.2% (0 to 16.7)	19.4% (9.2 to 25.2)	16.1% (2.9 to 22.6)	7.9% (0.1 to 16.2)
Deaths per 1000 person-years	1.4 (0.6 to 2.1)	0.9 (0.1 to 1.6)	0.4 (0 to 1.1)	0.9 (0.3 to 1.4)	0.7 (0.1 to 1.2)	0.3 (0 to 0.8)
Epidemic peak (months)	4 (3 to 7)	4 (3 to 12)	4 (4 to 6)	4 (3 to 9)	4 (3 to 11)	4 (4 to 6)
Peak deaths	900 (200 to 2000)	500 (40 to 1000)	100 (2 to 500)	500 (100 to 1000)	400 (30 to 800)	90 (2 to 400)
Peak non-ICU beds needed	30,000 (6000 to 50,000)	10,000 (900 to 30,000)	3000 (20 to 10,000)	20,000 (3000 to 30,000)	10,000 (700 to 20,000)	2000 (20 to 10,000)
Peak ICU beds needed	10,000 (3000 to 30,000)	7000 (500 to 20,000)	2000 (10 to 8000)	8000 (2000 to 20,000)	5000 (400 to 10,000)	1000 (10 to 6000)
Nigeria						
Symptomatic cases	60,200,000 (35,920,000 to 69,730,000)	46,250,000 (3,370,000 to 60,750,000)	25,470,000 (70,000 to 47,040,000)	51,970,000 (23,230,000 to 63,090,000)	44,410,000 (2,790,000 to 58,280,000)	24,180,000 (50,000 to 44,880,000)
Severe, non-critical cases	2,090,000 (1,030,000 to 2,670,000)	1,490,000 (60,000 to 2,180,000)	810,000 (2000 to 1,670,000)	1,270,000 (410,000 to 1,720,000)	1,050,000 (40,000 to 1,550,000)	550,000 (1000 to 1,150,000)
Severe, critical cases	890,000 (440,000 to 1,150,000)	640,000 (30,000 to 940,000)	350,000 (800 to 710,000)	540,000 (170,000 to 740,000)	450,000 (20,000 to 660,000)	240,000 (500 to 490,000)
Deaths	560,000 (270,000 to 710,000)	400,000 (20,000 to 580,000)	210,000 (500 to 440,000)	340,000 (100,000 to 460,000)	280,000 (9000 to 410,000)	150,000 (300 to 310,000)
Symptomatic attack rate	29.7% (17.7 to 34.4)	22.8% (1.7 to 29.9)	12.6% (0 to 23.2)	25.6% (11.5 to 31.1)	21.9% (1.4 to 28.7)	11.9% (0 to 22.1)
Deaths per 1000 person-years	2.7 (1.4 to 3.5)	2 (0.1 to 2.9)	1.1 (0 to 2.2)	1.7 (0.5 to 2.3)	1.4 (0 to 2)	0.7 (0 to 1.5)
Epidemic peak (months)	4 (3 to 8)	5 (3 to 12)	5 (4 to 7)	5 (3 to 10)	5 (3 to 12)	5 (4 to 7)
Peak deaths	20,000 (4000 to 30,000)	8000 (300 to 20,000)	2000 (10 to 9000)	9000 (2000 to 20,000)	6000 (200 to 10,000)	1000 (8 to 6000)
Peak non-ICU beds needed	480,000 (120,000 to 800,000)	240,000 (10,000 to 520,000)	60,000 (200 to 260,000)	260,000 (50,000 to 470,000)	170,000 (6000 to 370,000)	40,000 (100 to 180,000)
Peak ICU beds needed	250,000 (70,000 to 420,000)	130,000 (5000 to 280,000)	30,000 (100 to 140,000)	140,000 (30,000 to 250,000)	90,000 (3000 to 190,000)	20,000 (80 to 100,000)
Mauritius						
Symptomatic cases	540,000 (360,000 to 610,000)	440,000 (110,000 to 540,000)	280,000 (1000 to 450,000)	430,000 (230,000 to 500,000)	380,000 (90,000 to 470,000)	240,000 (1000 to 390,000)
Severe, non-critical cases	50,000 (30,000 to 60,000)	40,000 (8000 to 50,000)	20,000 (100 to 40,000)	30,000 (10,000 to 40,000)	20,000 (5000 to 30,000)	10,000 (100 to 30,000)
Severe, critical cases	20,000 (10,000 to 30,000)	20,000 (3000 to 20,000)	10,000 (50 to 20,000)	10,000 (5000 to 20,000)	10,000 (2000 to 10,000)	6000 (40 to 10,000)
Deaths	10,000 (8000 to 20,000)	10,000 (2000 to 10,000)	7000 (30 to 10,000)	7000 (3000 to 9000)	6000 (1000 to 9000)	4000 (20 to 7000)
Symptomatic attack rate	41.7% (27.7 to 46.6)	33.5% (8.3 to 41.8)	21.5% (0.1 to 34.9)	33.2% (17.6 to 38.7)	29.4% (7 to 36.5)	18.5% (0.1 to 30.3)
Deaths per 1000 person-years	10.6 (6.1 to 12.9)	7.9 (1.5 to 10.9)	5 (0 to 9)	5.6 (2.6 to 7.3)	4.9 (0.9 to 6.7)	3 (0 to 5.4)
Epidemic peak (months)	3 (2 to 5)	3 (2 to 10)	3 (2 to 5)	3 (2 to 7)	3 (2 to 11)	3 (2 to 5)
Peak deaths	400 (100 to 700)	200 (20 to 500)	90 (2 to 300)	200 (40 to 400)	100 (10 to 300)	50 (2 to 200)
Peak non-ICU beds needed	10,000 (4000 to 20,000)	7000 (500 to 10,000)	2000 (20 to 8000)	6000 (1000 to 10,000)	4000 (300 to 8000)	1000 (10 to 5000)
Peak ICU beds needed	7000 (2000 to 10,000)	4000 (300 to 7000)	1000 (9 to 4000)	3000 (600 to 5000)	2000 (200 to 4000)	700 (7 to 2000)

Whereas reducing transmission outside of the household by 20% would be more effective in reducing the total number of symptomatic cases than shielding, shielding could be as effective in reducing the total bed demand at the peak of the epidemic and total number of deaths as moderate physical distancing when *R*_0_ values are higher, as illustrated by estimates using global *R*_0_ estimates. When *R*_0_ values are low however, even moderate levels of social distancing are sufficient to bring *R*_0_ close to or below 1, resulting in larger effect sizes using social distancing alone compared to shielding alone.

More substantial levels of physical distancing (50%) would lead to far greater effects in the first 12 months of the epidemic. However, this strategy, unlike shielding, reduces overall transmission and thus does not necessarily result in a resolution of the epidemic through herd immunity during this period; instead, this intervention would need to be sustained into the second year until herd immunity is reached through either natural immunity or a vaccine, assuming immunity is long lived. This may feature a second, albeit smaller peak during the second year if no further mitigation measures are taken; this further peak is absent under the other strategies. However, predictions beyond the first year may vary considerably depending on the longevity of SARS-CoV-2 immunity and availability of potential pharmaceutical interventions, and should be interpreted with caution [3].

A combination of shielding and physical distancing would be most effective in reducing overall outcomes. Table 4 highlights the relative reduction in hospital bed demand for severe cases at the epidemic peak and total number of deaths in the first 12 months of the epidemic, under each scenario. If *R*_0_ values would be higher than initial *R*_*t*_ estimates, shielding 80% of the high-risk population and reducing contact between the shielded and unshielded populations by 80% could be as effective in reducing severe outcomes as general physical distancing reducing contacts by 20%.

**Table 4:** Relative reductions in key outcomes during the first 12 months of the epidemic under different strategies, compared to an unmitigated epidemic. All strategies include 50% self-isolation of symptomatic cases

Key outcome	Country	20% distancing	50% distancing	80% shielding	80% shielding + 20% distancing	80% shielding + 50% distancing
Projections using country ***R***_***t***_ estimates						
Total symptomatic cases	Niger	56.6% (31.5 to 99.6)	98.8% (75.1 to 99.8)	23% (15.9 to 99.3)	58.7% (33 to 99.7)	98.8% (75.6 to 99.8)
	Nigeria	85.1% (30.6 to 99.6)	99.5% (79.9 to 99.9)	30.8% (16.7 to 99.7)	86.9% (33.4 to 99.8)	99.6% (82.9 to 99.9)
	Mauritius	57.3% (9.9 to 99.5)	98.8% (24.5 to 99.7)	34.3% (16.9 to 99.5)	64% (21.2 to 99.6)	99% (34.5 to 99.8)
Hospital bed demand at epidemic peak	Niger	62.5% (38.8 to 99.6)	98.8% (76.4 to 99.8)	42.9% (38.3 to 98.8)	69.9% (52.3 to 99.4)	99% (80.8 to 99.8)
	Nigeria	87.4% (35.9 to 99.7)	99.5% (76.7 to 99.9)	54.1% (41.4 to 99.6)	89.5% (54.6 to 99.8)	99.6% (84.6 to 99.9)
	Mauritius	61.1% (15.1 to 99.6)	98.9% (29.9 to 99.8)	56.1% (43 to 99.3)	77.3% (47.5 to 99.6)	99.2% (57.6 to 99.8)
Total deaths	Niger	63.9% (38.4 to 99.6)	98.8% (76.8 to 99.8)	42.8% (37.9 to 99)	71.2% (51.9 to 99.5)	99% (81 to 99.8)
	Nigeria	90.3% (36.1 to 99.7)	99.5% (77.4 to 99.9)	56.8% (41.5 to 99.6)	90.4% (54.7 to 99.7)	99.6% (77.2 to 99.9)
	Mauritius	62.1% (14.2 to 99.5)	98.8% (30.2 to 99.7)	56.4% (43.3 to 99.6)	78% (47.2 to 99.7)	99.3% (57.2 to 99.8)
Projections using global ***R***_**0**_ estimates						
Total symptomatic cases	Niger	27.6% (18 to 77.9)	63.9% (41 to 99.7)	14.6% (10.9 to 25.5)	29.3% (20.3 to 76.2)	65% (42.8 to 99.6)
	Nigeria	23.2% (12.9 to 90.6)	57.7% (32.5 to 99.8)	13.7% (9.5 to 35.3)	26.2% (16.4 to 92.2)	59.8% (35.6 to 99.8)
	Mauritius	19.7% (10 to 70.1)	48.5% (24.8 to 99.6)	20.4% (16.8 to 36.6)	29.6% (21.4 to 74.7)	55.7% (34.9 to 99.6)
Hospital bed demand at epidemic peak	Niger	35.3% (25.9 to 83.3)	69.2% (49 to 99.7)	37.8% (35.4 to 45.2)	50.5% (44.3 to 85.4)	76.8% (62.1 to 99.7)
	Nigeria	28.7% (18.2 to 93.4)	61.2% (37.6 to 99.8)	39.2% (35.5 to 59.4)	49.5% (42 to 96)	73.6% (57 to 99.9)
	Mauritius	24.9% (14.9 to 74.7)	52.6% (30.1 to 99.6)	46.8% (42.9 to 58.2)	53.9% (47.5 to 84.6)	71.8% (57.8 to 99.7)
Total deaths	Niger	35.3% (25.6 to 85.8)	69.2% (48.7 to 99.7)	37.9% (35 to 45.9)	50.5% (44 to 87)	76.8% (61.9 to 99.7)
	Nigeria	28.7% (18.2 to 94.5)	61.3% (37.5 to 99.8)	39.2% (35.4 to 63.5)	49.5% (41.9 to 96.7)	73.6% (57 to 99.9)
	Mauritius	24.9% (14.3 to 76.2)	52.5% (29.5 to 99.6)	46.8% (42.5 to 58.7)	53.9% (46.9 to 85.7)	71.8% (57.3 to 99.7)

#### In combination with lockdowns

We also explored the impact a temporary lockdown could have on these same strategies. We assumed a 2-month lockdown, triggered at incidence 1 per 10,000 person-days, during which, in addition to self-isolation as above, all contacts outside the household are reduced by 80%; the remainder of the year consisted of the five alternative strategies above.

Figure S4 shows bed demand and deaths over time while Table S3 shows key outcomes in the first 12 months under this lockdown scenario. A lockdown would delay, but not prevent, the epidemic peak in all countries (under country specific *R*_*t*_ estimates, from 6 to 8–9 months in Niger, from 8 to 8–10 months in Nigeria, and from 5 to 5–7 months in Mauritius, while under global *R*_0_ estimates from 4 to 6–7 months in Niger, from 4 to 6–7 months in Nigeria, and from 3 to 4–5 months in Mauritius). However, it would not substantially affect total epidemic sizes or peak bed demand, compared to strategies without lockdowns, unless the epidemic is suppressed for the entire year.

#### Removing self-isolation of symptomatic individuals

Figure S4 shows bed demand and deaths over time for each modelled strategy in the absence of self-isolation, while Table S4 shows associated key outcomes.

Removing self-isolation substantially increased the number of cases and deaths in all scenarios. However, this relative difference was approximately similar for scenarios with physical distancing only (35%, 16%, and 25% higher mortality in Niger, Nigeria, and Mauritius) and scenarios with shielding only (40%, 13%, and 27%). The resulting increased transmission brought the epidemic peak forward in all countries, under both scenarios using country specific *R*_*t*_ estimates or global *R*_0_ estimates.

#### Additional sensitivity analyses

As expected, varying our base assumption on the infectiousness of asymptomatic cases greatly affects the impact of self-isolation of symptomatic cases: if the former are not at all infectious, self-isolation could be highly impactful (up to 100%), and vice versa (Figure S7). The impact of general distancing is insensitive to this assumption, while for shielding impact would decrease if asymptomatic people are not infectious and shielded people increase their contacts with each other (Figure S8). Overall, the effect of mitigation strategies (all of which assume some symptomatic self-isolation in our base scenario) would be higher, when asymptomatic cases contribute to transmission less (Figure S9).

Our assumption of a shift in severity risk to younger ages had a negligible impact on the relative effectiveness of self-isolation and physical distancing (Figure S13), but the 10-year age-shift yielded a lower effectiveness of shielding compared to no age shift (Figure S14), since it increases risk among younger, unshielded age groups. The severity and CFR assumptions substantially affect the absolute size of the epidemic predicted by the model (Table S6). In scenarios assuming country-specific *R*_*t*_ estimates, changing these assumptions would result in a variation from 5000 to 30,000 deaths in Niger, 80,000 to 400,000 in Nigeria and 3000 to 12,000 in Mauritius if no age shift or CFR increase are assumed, compared to a 10-year shift and doubling of CFR (our worst-case scenario).

## Discussion

### Main findings

We explored the impact of different non-pharmaceutical control interventions and strategies (packages and sequences of interventions) that may effectively be implemented in African countries to mitigate COVID-19 epidemics. Short of an indefinite-duration lock-down, none of the interventions would likely avert very large epidemics that result in high mortality and extreme pressure on health services. While numerous initiatives are underway to rapidly scale up COVID-19 case management capacity, e.g. by increasing oxygen availability at district hospital level, the actual coverage and effectiveness of such treatment is difficult to predict at present, given various possible rate-limiting factors (competent staff availability, infection prevention and control and steady supplies of personal protective equipment, hospital infrastructure, care-seeking preferences) [36]. At baseline, Niger, Nigeria and Mauritius had some 7000, 101,000 and 4000 hospital beds respectively [5], of which presumably only a fraction could be assigned to COVID-19 care without severely compromising essential routine health services. Whether these capacities would be sufficient highly depends on the real *R*_0_ values of Covid-19 epidemics in these countries. When we assume early country-specific *R*_*t*_ estimates for *R*_0_, non-pharmaceutical interventions could effectively be used to maintain hospital bed demand below these levels. However, when we assume global *R*_0_ estimates for *R*_0_, the intervention strategies we explored (see below) would exceed these baseline levels even under the most stringent (and unrealistic) implementation.

Reassuringly, our analysis suggests that both self-isolation of symptomatic people and moderate physical distancing could translate into very sizable reductions in severe cases and deaths, even when higher levels of *R*_0_ are assumed. The shielding option would likely require high levels of adherence and isolation to yield appreciable reductions in health service pressure, but could have a higher potential to reduce mortality in the short-term than other interventions, as it focuses on those who experience the highest CFR. This option also promotes herd immunity through mixing of other age groups and thus carries a lesser risk of further epidemic peaks once measures are lifted.

Different shielding arrangements could be considered, ranging from individual arrangements wherever people already live in multi-room houses or compounds, to neighbours or extended family-members grouping the most vulnerable individuals in vacated houses, to larger, albeit epidemiologically riskier re-housing (e.g. in quarantined street blocks). To avoid the problem of transmission within the shielded population, all such arrangements would need to eliminate any traffic of external people in and out of shielded accommodation as much as possible ensuring basic needs are met, while also instituting infection control barriers, e.g. a designated exchange point for supplies and safe social interactions and limiting contact as well as transmission within the shielded population through distancing, hygiene measures, face coverings if appropriate, etc. While our model does not explore the micro-level dynamics of how seeding of infection into these accommodations would affect residents, we showed, as expected, that the amount of contact among high-risk shielded people matters: zero contact, equivalent to individual shielding, equates to the highest effect of the intervention, while an increase of contact from baseline, e.g. if shielded people are rehoused in more crowded conditions than in their households of origin, dampens the utility of this approach and could even lead to an increase in cases compared to the baseline of no intervention.

We next combined the above interventions into a set of strategies, with a horizon of 12 months, that countries could consider. We assumed that any strategy would feature, at a minimum, self-isolation of symptomatic cases (we caution however that promotion of self-isolation should not discourage care-seeking for other life-threatening health problems, e.g. malaria, particularly among children). Our predictions suggest that countrywide lockdowns of 2 months, if effective, would temporarily suppress and delay epidemics for around 2–3 months, as noted in Europe: this reprieve would potentially enable countries to mobilise resources and plan the implementation of the next phase of their strategy. These findings do not in themselves support lockdown measures as a universal solution, however: such measures may be ineffective (i.e. fail to achieve a contact rate reduction consistent with effective reproduction number < 1, the condition for suppression) or more harmful than beneficial, including in health terms, if they severely disrupt economies and livelihoods or encounter mistrust and community resistance. Rather, our predictions merely indicate that well-implemented lockdowns would achieve the intended effect.

If lockdowns would not have been implemented, or after they end, we predict that a combination of general physical distancing and shielding high-risk individuals could be a potentially achievable mitigation strategy for countries to consider. Physical distancing entails a difficult trade-off between reducing attack rates (and hence epidemic peak size) and extending the duration of the epidemic, which in turn increases the period over which shielding should be maintained (as individuals will require to be shielded until well after the epidemic peak has finished). While stringent physical distancing (e.g. 50% reduction in extra-household contacts) would have a large impact, such reductions may only be achieved through socio-economically damaging and potentially unacceptable restrictions to work, education and/or other forms of public life. By contrast, a 20% reduction in contacts may be more achievable and sustainable—in some settings, this could involve a combination of hygiene promotion; increased access to water, soap and other cleaning supplies (e.g. through state subsidies); face coverings; and curtailment of some gatherings outside of work and school. These moderate reductions could already result in large reductions when *R*_0_ levels in the studied countries are lower than estimated globally.

It is unknown at present whether shielding is at all feasible and can attain our suggested target of 80% contact reduction between high- and low-risk people for 80% of high-risk people. We know of no precedent for this intervention, but instances of its implementation in different countries (Yemen, Ethiopia) are underway; preliminary qualitative research findings from various settings suggest that risk awareness is a barrier and that within-household shielding is preferable to other solutions (F Checchi, pers. comm.). Even at lower effectiveness levels, however, shielding would still offer benefits, particularly in terms of mortality, and accordingly need not be discounted as an option, particularly if it can be designed and led by communities themselves [37] with reference to cultural and religious norms around protection of the elderly and vulnerable, thereby requiring fewer resources than a top-down intervention. Complementary measures to protect high-risk people could include maintenance of high-adherence treatment for NCDs, tuberculosis and HIV; cash transfers to offset loss of income and facilitate isolation arrangements; and mobile medical services to bring routine healthcare to those shielding. To avoid stigma and cross-infection, people living with HIV and active TB cases may need to shield individually, and measures should be introduced to address protection risks, such as intimate partner violence [38].

Both distancing and shielding would be strongly dependent on the local *R*_0_: even moderate reductions in general contacts, and some degree of self-isolation when symptomatic, could achieve very large effects if transmissibility is lower than has hitherto been observed in Europe or China, e.g. in rural parts of Africa, where this intervention would therefore be relatively more cost-effective; by contrast, shielding effectiveness is relatively insensitive to *R*_0_, but would have to be sustained for a longer period if the epidemic is protracted, as one would expect in low transmissibility scenarios. Our analysis showed that, even at lower levels of *R*_0_, shielding would be beneficial to complement distancing, albeit with a lower marginal impact as would be observed in higher transmission settings, as might be expected in urban settings.

We only show estimates for the first 12 months in our analysis to make short-term predictions of the impact of different intervention strategies. There are still many unknowns about how SARS-CoV-2 would behave in in different contexts in Africa (rural, urban, peri-urban, displacement settings, etc.) and how people will respond to policy measures; hence, policy makers will continuously need to revisit the strategy to take current developments into account. Our analysis does not necessarily reflect the total epidemic size, as additional severe and critical cases could accrue during the second year, especially under strategies that focus heavily on physical distancing or if natural immunity to infection is short-lived [3].

### Comparison with other studies

Although several studies have looked at the spread of COVID-19 in African countries [39–44], we are only aware of one other modelling study which considers the impact of different interventions on the spread of COVID-19 in Africa. Walker et al. [45] used a similar SEIR model and predicted a near 90% reduction in cases for sub-Saharan Africa assuming a 75% reduction in contacts starting at an incidence of 0.2 deaths per 100,000 population per week and sustained over the first 250 days of an epidemic. Comparisons between these studies are complicated due to different time periods of models and strategies investigated, but both point to a large unmitigated epidemic which can be reduced substantially due to strong physical distancing measures. However, even with these measures in place, all models suggest a high burden of disease and mortality across Africa.

### Study limitations

While SEIR models have successfully been used to model COVID-19 epidemics to date in Europe, our predictions for African countries are subject to potential inaccuracy. Transmissibility may vary considerably across Africa, and it is possible that countries with very concentrated urban populations would see an acute exponential rise in cases, with a secondary, flatter curve affecting outlying rural regions: models accounting for these very distinct settlement types may be more useful for national planning. Consequently, the duration of time that countries will be affected by COVID-19 according to our model should be treated with caution. To date, estimates based on reported deaths [46] or cases [47, 48] suggest that African countries had transmissibility levels that may have been lower as observed in Europe and elsewhere. However, most African countries adopted stringent containment measures early on in the epidemic, meaning that even early *R*_*t*_ estimates as used in our analysis may not reflect a truly unmitigated epidemic, and underestimate the actual transmission potential of Covid-19 in the countries studied.

We were not able to reliably estimate country-specific *R*_0_ levels, due to relatively low levels of reported cases and deaths (Mauritius) and unknown levels of testing, underreporting of cases or impact of already implemented interventions within all countries. Instead, we conducted two separate analyses showing results using early levels of country-specific *R*_*t*_ estimates and global estimates of *R*_0_. The country-specific *R*_*t*_ estimates are generally lower than the globally estimated *R*_0_, but the median estimates of the former do fall within 95% uncertainty intervals of the latter. Whereas assumed *R*_0_ values have a large effect on the total size of the epidemic, they do not substantially change the qualitative decision of comparing different non-pharmaceutical interventions. However, shielding strategies do not significantly affect transmission in the overall population and may therefore not be preferred in low transmission settings, where moderate nondisruptive levels of distancing and self-isolation could be sufficient to bring *R*_0_ levels below 1.

While we accounted for age distributions, we did not have country-specific data on contact patterns among age groups. Instead, we used synthetic contact matrices extrapolated from European data by using local data on household, workplace and school composition in the African settings considered. A sensitivity analysis with empirical African contact pattern data suggested broadly similar intervention effects, but higher overall epidemic sizes; these matrices were collected in specific areas and may not be representative of contact patterns within the entire country or indeed Africa as a whole.

There is no clear consensus on the impact of asymptomatic infections on transmission. In accordance with other studies [19, 20], we assumed asymptomatic infections to be 50% less infectious. Our sensitivity analysis shows that this assumption did significantly affect the estimated impact of self-isolation strategies. We assumed that individuals who self-isolate would do so on the day of symptom onset, but not when pre-symptomatic. We made no assumptions about the effect of testing and behaviour change on individual behaviour, nor did we assume a potential delay between onset of symptoms and start of self-isolation.

Our results are also affected by disease severity assumptions. We applied age-specific risks of developing severe disease per infection, as estimated using data from China and the Diamond Princess outbreak, but shifted these to earlier life by a decade to represent plausible differences in biological age in Africa resulting from life-course exposures. This crude approach may be confounded by differences in the age-specific prevalence of co-morbidities in African countries, as well as inter-country differences in comorbidity prevalence. Specifically, conditions with potential (tuberculosis [49]) and as yet undocumented (HIV, undernutrition, sickle-cell disease) interactions with SARS-CoV-2 infection are far more prevalent in Africa than China and affect relatively young age groups [32]: these could increase COVID-19 severity overall and shift the distribution of severe cases to younger age. Additionally, the proportion of detected and correctly managed cases of non-communicable diseases of known import to COVID-19 progression (cardiovascular disease, diabetes, chronic obstructive pulmonary disease, chronic kidney disease) is far lower in most of Africa than in China and Europe: this may further exacerbate disease severity. These differing patterns of co-morbidity may also affect the proportion of patients requiring intensive care and the CFR. Overall, our sensitivity analysis shows that uncertainty on these parameters has modest effects on the relative impact of interventions, but can have large effects on model predictions of absolute epidemic size. Our findings will thus need to be refined as more evidence accrues on the virus’ severity and CFR in African settings. Whereas these assumptions have large effects on the estimated total epidemic sizes, their impact on the relative effectiveness of different interventions is only minor.

Generally, our modelling framework is unsuited to incorporating information on underlying vulnerability factors such as population density, mobility within the country, armed conflict intensity, etc. [50], which may affect transmissibility, severity and/or the feasibility and effectiveness of response interventions. Our model would instead need to be updated with empirical evidence from each country, as it accumulates. For example, countries’ ability to scale up case management services (at least non-invasive respiratory support) from a nearly universally low baseline [45] may affect the actual CFR. Moreover, models that can track epidemics across space and time at a sub-national level would be preferable, with a structure (e.g. individual-based modelling) that can incorporate granular information about any mobility and behavioural data, the local age distribution of co-morbidities, treatment availability, the timing and uptake of specific interventions or, conversely, propagation (superspreading) events such as mass gatherings.

Severity and CFR assumptions mostly affect the usefulness of the shielding option. Shielding criteria should include a diagnosis of co-morbidity, and as such our findings, based solely on an age criterion, are under-estimates insofar as they exclude younger people with known comorbidities. However, in practice the low non-communicable disease treatment coverage in Africa means the age criterion would largely define who is shielded: lowering this threshold, e.g., to 50 years, would capture a larger fraction of undiagnosed co-morbidities. Tiered shielding approaches, whereby middle-aged moderate-risk people benefit from partial distancing measures (e.g. support to stay home from work), may also be worth considering.

## Conclusions

COVID-19 epidemics in African countries may bear very serious and multi-faceted impacts. As of the time of writing, many African countries are exiting stringent lockdowns and having to make decisions about how to manage epidemics despite limited testing and treatment capacity, which, among other complexities, hampers situational awareness. Nevertheless, preventive strategies to substantially mitigate the impacts of COVID-19 are not foreclosed to African governments and societies, particularly if they receive assistance from humanitarian and development actors, diaspora communities, faith-based institutions, the private sector and others within these societies who have means to assist the response. Self-isolation and moderate physical distancing can be effective interventions. The shielding option can be proactively explored to test locally appropriate solutions. As the epidemic progresses, real-time modelling and strategy evaluation should be made available to African countries that do not yet have this expertise: this requires coordination and proactive support from the worldwide scientific community, as well as close exchange of information between modelling teams and country-based surveillance, so as to gradually refine predictions.

## Supplementary information

**Additional file 1.**

## Data Availability

All analysis code and data are available at https://github.com/kevinvzandvoort/covid19-response-strategies-african-settings.

## Abbreviations

CFR: Case-fatality ratio
ICU: Intensive care unit
SEIR: Susceptible-Exposed-Infectious-Recovered
