# Supplementary material for: Response strategies for COVID-19 epidemics in African settings: a mathematical modelling study
Source: BMC Med. 2020 Oct 14;18:324. doi: 10.1186/s12916-020-01789-2 (PMC7553800; doi:10.1186/s12916-020-01789-2)
Supplement: Supplementary file 1 — Additional file 1. [file 12916_2020_1789_MOESM1_ESM.docx]

Response strategies for COVID-19 epidemics in African settings: a mathematical modelling study

ADDITIONAL FILE 1

Kevin van Zandvoort^1^**^*^^**, Christopher I Jarvis^1^^, Carl A B Pearson^1,3^, Nicholas G Davies^1^, CMMID COVID-19 working group, Ruwan Ratnayake^1^, Timothy W Russell^1^, Adam J Kucharski^1^, Mark Jit^1^, Stefan Flasche^1^, Rosalind M Eggo^1^, Francesco Checchi^2^

* Corresponding author: [Kevin.Van-Zandvoort@lshtm.ac.uk](mailto:Kevin.Van-Zandvoort@lshtm.ac.uk)

^ contributed equally

The CMMID COVID-19 working group is: Emily S Nightingale, James D Munday, Amy Gimma, Alicia Rosello, Julian Villabona-Arenas, Sebastian Funk, Katherine E. Atkins, Charlie Diamond, Sophie R Meakin, Simon R Procter, Fiona Yueqian Sun, Akira Endo, Damien C Tully, Eleanor M Rees, Arminder K Deol, Anna M Foss, Petra Klepac, W John Edmunds, Kiesha Prem, Jon C Emery, Megan Auzenbergs, Sam Abbott, Samuel Clifford, Thibaut Jombart, Gwen Knight, Stéphane Hué, Quentin J Leclerc, Kathleen O'Reilly, Billy J Quilty, Rein M G J Houben, Joel Hellewell, Nikos I Bosse , Hamish P Gibbs, Yang Liu, Graham Medley. Order of working group determined at random.

^1^ Centre for Mathematical Modelling of Infectious Diseases, Department of Infectious Disease Epidemiology, London School of Hygiene and Tropical Medicine, Keppel Street, WC1E 7HT London, UK

^2^ Health in Humanitarian Crises Centre, Department of Infectious Disease Epidemiology, London School of Hygiene & Tropical Medicine, Keppel Street, WC1E 7HT

^3^ South African Centre for Epidemiological Modelling and Analysis, Stellenbosch University, Stellenbosch, Republic of South Africa

Table of contents

[Details on mathematical model 3](#_Toc44621750)

[Model structure 3](#_Toc44621751)

[Model states and transitions 3](#_Toc44621752)

[Model dynamics 4](#_Toc44621753)

[Clinical outcomes 4](#_Toc44621754)

[Transmissibility under different conditions 4](#_Toc44621755)

[Force of infection 4](#_Toc44621756)

[Effect of interventions 5](#_Toc44621757)

[Parameter values 5](#_Toc44621758)

[Transmission dynamics and state transition parameters 5](#_Toc44621759)

[Severity parameters 6](#_Toc44621760)

[Additional analyses 8](#_Toc44621761)

[Epidemic trajectories under different intervention scenarios 8](#_Toc44621762)

[Sensitivity analyses 21](#_Toc44621763)

[Results using empirical contact matrices 21](#_Toc44621764)

[Sensitivity to assumed infectiousness of asymptomatic cases 29](#_Toc44621765)

[Sensitivity to transmissibility assumption ($R0$) 33](#_Toc44621766)

[Sensitivity to disease severity assumptions 36](#_Toc44621767)

[References 42](#_Toc44621768)

# Details on mathematical model

## Model structure

### Model states and transitions

We used a stochastic compartmental dynamic transmission model, stratified into $a$ age groups $i$ (5-year age groups, with one additional age group for those aged ≥75 years). The model’s structure is depicted in Figure S1.

$$S$$

Susceptible

$$E$$

Infected, not yet infectious

$$I_{P}$$

Infectious, pre-symptomatic

$$I_{C}$$

Infectious, symptomatic

$$R$$

Removed (dead or immune)

$$I_{S}$$

Infected, asymptomatic

$$y_{i}$$

$$1-y_{i}$$

#### **Figure S1. State transitions in the model.** Individuals in the stochastic compartmental model are classified into susceptible, exposed (infected but not yet infectious), infectious (pre-symptomatic, symptomatic, or asymptomatic), and removed states. The model is stratified into 5-year age bands.

Within every age group, the population is distributed into compartments of susceptible individuals ($S$), who become exposed ($E$) after effective contact with an infectious person (exposed here means infected but not yet infectious). At the end of the latency period, people divide into symptomatic and asymptomatic cases with complementary probabilities $y_{i}$ and $1-y_{i}$, at which point infectiousness starts. Symptomatic cases experience a pre-symptomatic but infectious state $I_{P}$ followed by a symptomatic, infectious state $I_{C}$. Asymptomatic cases ($I_{S}$) are assumed to be half as infectious as symptomatic cases (sensitivity analysis of this assumption is presented below). All individuals have the same duration of infectiousness. The clinical severity of cases is not assumed to affect infectiousness. Individuals either recover or die, transitioning to the removed ($R$) compartment. Transitions to different clinical states (symptom onset to severe case, critical or not; from onset of severe symptoms to recovery or death) occur with delays drawn from the literature: these delays have no bearing on the force of infection / transmissibility. The amount of time a given individual spends in states $E$, $I_{P}$, $I_{C}$ , or $I_{s}$ is drawn from distributions $d_{E}$, $d_{P}$, $d_{C}$ and $d_{S}$, respectively (Table S1).

### Model dynamics

The model can be represented by the following system of differential equations:

$$\frac{{dS}_{i}}{dt}=- {\lambda_{i,t}S}_{i,t}$$

$$\frac{{dE}_{i}}{dt}={\lambda_{i,t}S}_{i,t}-E_{i,t-d_{E}}$$

$$\frac{{dI}_{P,i}}{dt}={y_{i}E}_{i,t-d_{E}}-I_{P,i,t-d_{P}}$$

$$\frac{{dI}_{C,i}}{dt}=I_{Pi,t-d_{P}}-I_{C,i,t-d_{C}}$$

$$\frac{{dI}_{S,i}}{dt}={(1-y_{i})E}_{i,t-d_{E}}-I_{S,i,t-d_{S}}$$

$$\frac{{dR}_{i}}{dt}=R_{i,t-1}+I_{C,i,t-d_{C}}+I_{S,i,t-d_{S}}$$

where $\lambda_{i,t}$ is the age-specific instantaneous force of infection (incidence) experienced by a susceptible individual, as detailed below. The model tracks a country’s population over discrete 6-hour $dt$ increments.

### Clinical outcomes

The model tracks clinical outcomes separately from the above equations. A proportion of symptomatic cases are assumed to progress to severe disease (a further proportion of which are critical) based on age-specific risks, and with a varying delay from symptom onset. Severe, non-critical and critical disease have different durations. Death is modelled separately, as an age-specific risk that applies to each symptomatic case, with a varying time from symptom onset to death.

## Transmissibility under different conditions

### Force of infection

The force of infection is defined as the rate at which susceptible people enter the exposed compartment, and is computed for any age group $i$ and time $t$ as

$$\lambda_{i,t}=u\sum_{j=1}^{j=a} c_{ij,t}\frac{I_{P,j,t}+I_{C,j,t}+{fI}_{S,j,t}}{N_{j}}$$

where $u$ is the probability of infection per contact with an infectious person, $c_{ij}$ is the number of contacts that an individual in age group $i$ has with individuals in age group $j$ per time increment (drawn from the matrix $\mathbf{C}$ of contact rates between different age groups), and $\frac{I_{P,j,t}+I_{C,j,t}+{fI}_{S,j,t}}{N_{j}}$ is the probability that any age $j$ individual contacted is in fact infectious, with $f$denoting the relative infectiousness of asymptomatic cases, compared to symptomatic cases.

Both synthetic and empirical contact matrices were asymmetric, i.e. $c_{ij}\neq c_{ji}$ , a result of questionnaire data collection whereby people of age $i$ may not report the same intensity of contact with age $j$ as vice versa. Therefore, before applying either matrix to the model we converted them to symmetric forms using the method of Wallinga et al. [1] In an unmitigated epidemic, the contact matrix is assumed to remain constant.

The basic reproduction number $R_{0}$ is defined as the average number of secondary infections generated by a typical infectious individual in a fully susceptible population and is calculated as the absolute value of the dominant eigenvalue of the next generation matrix (NGM), which was derived by linearizing the system of model equations at epidemic equilibrium [2]. Lastly, $u$ is derived for any stochastic run from the ratio of this eigenvalue and the $R_{0}$ value selected for that run.

### Effect of interventions

The three interventions we considered act on the force of infection by modifying contacts among age groups. Both the synthetic and empirical contact matrices stratify contacts by setting (household, work, school, other), which we simplified as a household and extra-household stratum. Accordingly:

- **Self-isolation** of symptomatic people was modelled as a reduction in the infectiousness of individuals in the $I_{C}$ state;
- **Population-wide physical distancing**, including the more extreme lockdown version, was modelled as a reduction in all extra-household contacts among all individuals in the population;
- **Shielding** was modelled by varying three parameters: (i) the fraction of elderly people (≥ 60 years old) who are actually shielded; (ii) among the fraction shielding, the relative reduction in all contacts (within and outside the household) with anyone who is not shielded, implemented as a multiplier; and (iii) within the fraction shielding, the change in contacts with others who are shielding, relative to what it was before shielding, also implemented as multiplier. In practice, we implemented the above by further dividing the population into shielding and non-shielding strata.

## Parameter values

The model was implemented stochastically by selecting random values of parameters from their uncertainty distributions (Table S1).

### Transmission dynamics and state transition parameters

Model parameters relevant to transmission and state transitions are listed in Table S1.

#### **Table S1. Model parameters relevant to transmission and state transitions.** Adapted from Davies et al. [3].

| Parameter | Description | Value | Reference |
| --- | --- | --- | --- |
| $d_{E}$ | Pre-infectious period in days | ~ Gamma($\mu$ = 4, $k$ = 4) | [4–6] |
| $d_{P}$ | Duration of pre-symptomatic infectiousness in days | ~ Gamma($\mu$ = 1.5, $k$ = 4) | [7] |
| $d_{C}$ | Duration of symptomatic infectiousness in days | ~ Gamma($\mu$ = 3.5, $k$ = 4) | [4–6] |
| $d_{S}$ | Duration of asymptomatic infectiousness in days | ~ Gamma($\mu$ = 5, $k$ = 4) | Assumed to be the same as duration of total infectious period for clinical cases |
| $u$ | Probability of transmission per contact with an infectious individual | See text | Derived |
| $y_{i}$ | Probability of developing symptoms if infected, for age group $i$ | Age-dependent, as estimated by Davies et al. | [8] |
| $f$ | Infectiousness of asymptomatic cases, relative to pre-symptomatic and symptomatic cases | 50% | Assumed |
| $c_{ij}$ | Number of age-j individuals contacted by an age-I individual per day | Country-specific synthetic contact matrix  Alternatively, sampling with equal probability and with replacement from three empirical contact matrices. | [9]  Uganda: [10]  Kenya: [11]  Zimbabwe: [12] |
| $R_{0}$ | Basic reproduction number | Country specific early $R_{t}$ estimates or global $R_{\boldsymbol{0}}$ estimates derived from  ~ Normal($\mu$ = 2.6, $SD$ = 0.5) | [13]  [14] |
| $N_{i}$ | Number of age $i$ individuals | Demographic estimates | [15] |
| $dt$ | Time step for discrete-time simulation | 0.25 days |  |
|  | Delay from symptom onset to becoming a severe case in days | ~ Gamma($\mu$ = 7, $k$ = 7) | [16, 17] |
|  | Duration of severe, non-critical disease in days | ~ Gamma($\mu$ = 8, $k$ = 8) | Duration based on UK National Health Service data for disease code J12: viral pneumonia, not elsewhere classified [18] |
|  | Proportion of severe cases that become critical | 30% | [17] |
|  | Duration of severe, critical disease in days | ~ Gamma($\mu$ = 10, $k$ = 10) | [17] |
|  | Delay from symptom onset to death in days | ~ Gamma($\mu$ = 22, $k$ = 22) | [16, 19] |

### Severity parameters

We used as our baseline the same severity estimates as in Davies et al. [3]: these reflect the early COVID-19 outbreak in China [19], corrected based on data from the Diamond Princess cruise ship outbreak [20]. Note that like Davies et al., we decouple severity from case-fatality, i.e. CFRs are applied to symptomatic cases, irrespective of their severity, as per the above-cited sources.

However, in African and low-income countries, someone’s vulnerability to infection may correspond to that of an individual with greater chronological age in a high-income setting due to life-course effects like malnutrition, infections and often unmanaged non-communicable diseases. It is not yet known whether this increased vulnerability will affect COVID-19 age-specific disease risk in African populations. To provide conservative estimates, we shifted age-specific severity risks by 10 years towards younger ages (below we present sensitivity analyses assuming shifts of 0 and 5 years instead). In addition, we multiplied the CFRs by a factor of 1.5 to account for lower access to care (we also did sensitivity analyses assuming factors of 1.0, i.e. no change, and 2.0: see below). CFRs were also shifted by 0, 5 and 10 years towards younger ages. Corresponding age-specific severity estimates are presented in Table S2.

#### **Table S2. Model parameters relevant to age-specific disease outcomes.** Values used for the main analysis are in **bold**.

| Age group (y)^†^ | | | Proportion of symptomatic cases who become severe | Case-fatality ratio (proportion of symptomatic cases who die) | | |
| --- | --- | --- | --- | --- | --- | --- |
| 0y shift  (base) | 5y shift | **10y shift** |  | x 1.0  (base) | **x 1.5** | x 2.0 |
| < 5 |  |  | 0.0% | 0.0% | **0.00%** | 0.00% |
| 5 to 9 | < 5 |  | 0.0% | 0.0% | **0.00%** | 0.00% |
| 10 to 14 | 5 to 9 | **< 5** | 0.8% | 0.09% | **0.14%** | 0.19% |
| 15 to 19 | 10 to 14 | **5 to 9** | 0.8% | 0.09% | **0.14%** | 0.19% |
| 20 to 24 | 15 to 19 | **10 to 14** | 0.8% | 0.10% | **0.15%** | 0.20% |
| 25 to 29 | 20 to 24 | **15 to 19** | 0.8% | 0.10% | **0.15%** | 0.20% |
| 30 to 34 | 25 to 29 | **20 to 24** | 1.0% | 0.12% | **0.18%** | 0.25% |
| 35 to 39 | 30 to 34 | **25 to 29** | 1.0% | 0.12% | **0.18%** | 0.25% |
| 40 to 44 | 35 to 39 | **30 to 34** | 1.9% | 0.23% | **0.35%** | 0.46% |
| 45 to 49 | 40 to 44 | **35 to 39** | 1.9% | 0.23% | **0.35%** | 0.46% |
| 50 to 54 | 45 to 49 | **40 to 44** | 5.4% | 0.68% | **1.01%** | 1.35% |
| 55 to 59 | 50 to 54 | **45 to 49** | 5.4% | 0.68% | **1.01%** | 1.35% |
| 60 to 64 | 55 to 59 | **50 to 54** | 15.1% | 1.87% | **2.81%** | 3.74% |
| 65 to 69 | 60 to 64 | **55 to 59** | 15.1% | 1.87% | **2.81%** | 3.74% |
| 70 to 74 | 65 to 69 | **60 to 64** | 33.3% | 4.14% | **6.21%** | 8.28% |
| 75 to 79 | 70 to 74 | **65 to 69** | 33.3% | 4.14% | **6.21%** | 8.28% |
| ≥ 80 | 75 to 79 | **70 to 74** | 61.8% | 7.68% | **11.52%** | 15.36% |
|  | ≥ 80 | **75 to 79** | 61.8% | 7.68% | **11.52%** | 15.36% |
|  |  | **≥ 80** | 61.8% | 7.68% | **11.52%** | 15.36% |

† The oldest age group used in the transmission model is ≥ 75 years. Where values are different for those aged 75-79 and ≥ 80, we applied an average of the corresponding values, weighted for the relative proportions of the population in these age groups.

## Additional analyses

## Epidemic trajectories under different intervention scenarios

The trajectory of the epidemic for each of the three countries, and using synthetic matrices, is shown below, under different intervention scenarios, for scenarios using country specific $R_{t}$ estimates (in black lines) and scenarios using global $R_{\boldsymbol{0}}$ estimates (in coloured lines):

- Figure S2 shows predicted bed demand and mortality over 12 months, for each of the strategies, including an assumption of 50% self-isolation of symptomatic cases. Shielding strategies assume shielding of 80% of the population aged ≥ 60 years, irrespective of underlying comorbidities, with an 80% reduction in contacts between the shielded and unshielded population, and no change in contacts within the shielded population;
- Figure S3 is equivalent to Figure S2, but with a two-month lockdown (80% reduction in all contacts) preceding the implementation of interventions. Lockdown starts when daily incidence of symptomatic cases reaches 1 case per 10 000 people, while other strategies start once the lockdown is lifted. Table S3 shows key outcomes by country under this scenario.
- Figure S4 is equivalent to Figure S2, but without any self-isolation of symptomatic cases. Table S4 shows key outcomes by country under this scenario.

In each figure, thick solid lines show the runs representing the median daily number of hospital beds needed for severe but non-critical cases, critical cases, and the number of deaths during the first 12 months of the epidemic. Dashed lines show runs representing 50% quantiles, while dotted lines show runs representing 95% quantiles, of each respective value over all model runs used in the scenario.


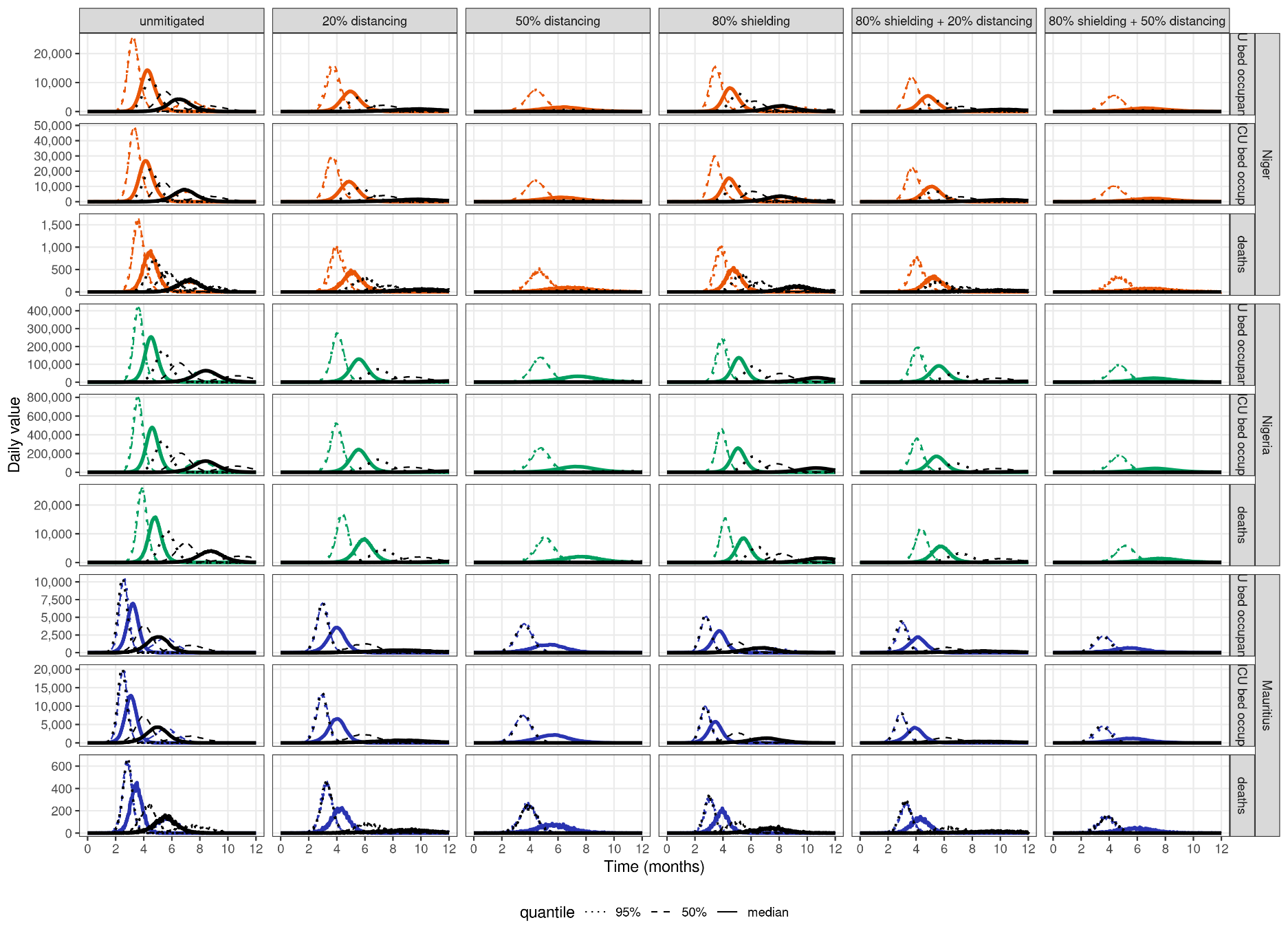


**Figure S2. Bed demand and deaths during the first 12 months of the epidemic, under different strategies**. Lines show the median, 95%, and 50% quantiles of total number of the corresponding outcome. Black lines show estimates for scenarios using country specific $R_{t}$ estimates, while coloured lines show estimates for scenarios using global $R_{\boldsymbol{0}}$ estimates.

**
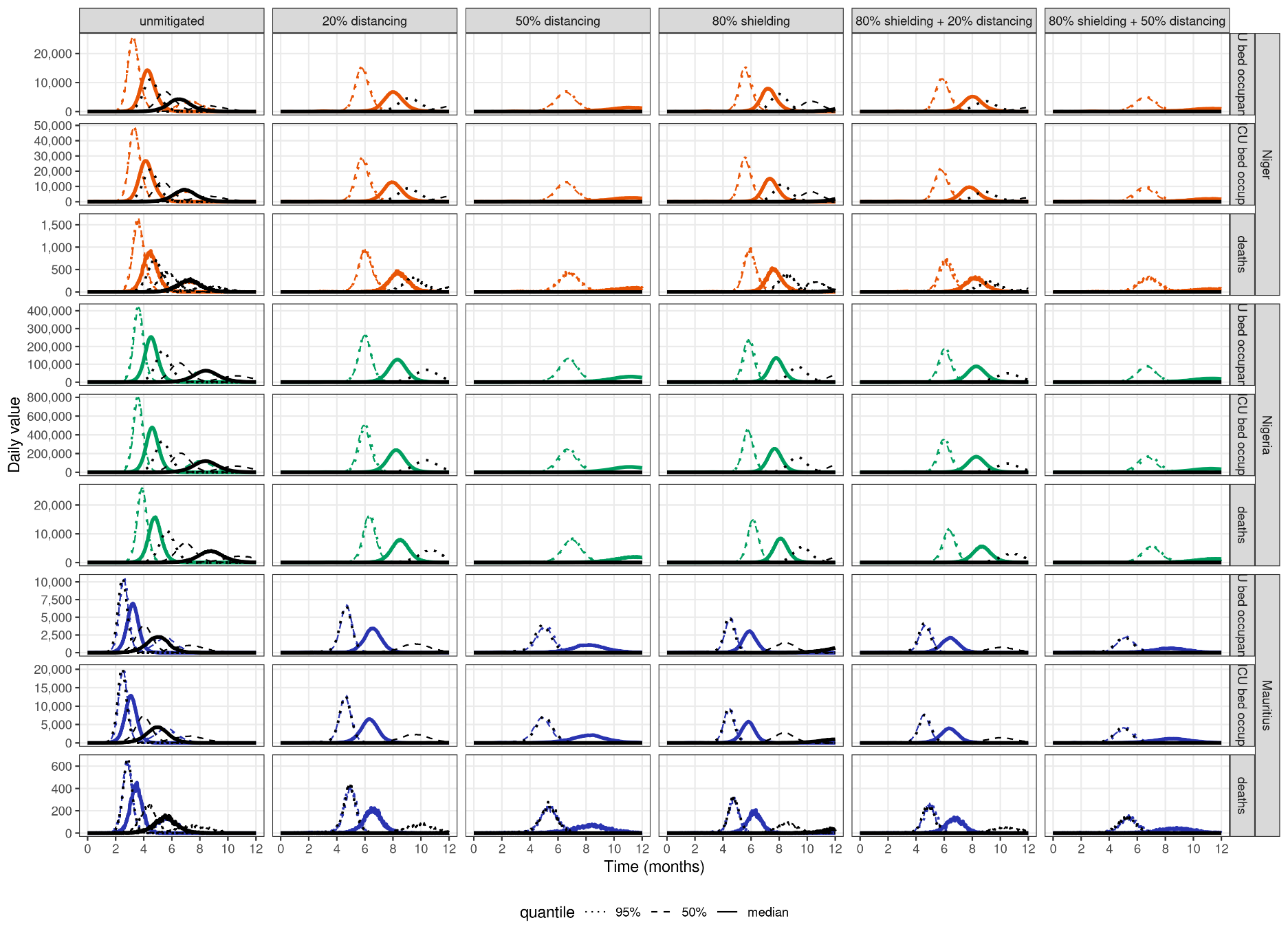
 Figure S3. Bed demand and deaths during the first 12 months of the epidemic, under different strategies preceded by a two-month lockdown.** Lines show the median, 95%, and 50% quantiles of total number of the corresponding outcome. Black lines show estimates for scenarios using country specific $R_{t}$ estimates, while coloured lines show estimates for scenarios using global $R_{\boldsymbol{0}}$ estimates.

#### **Table S3. Key outcomes during the first 12 months of the epidemic, under different strategies preceded by two-month lockdown.**

| Niger | | | | | | | | |
| --- | --- | --- | --- | --- | --- | --- | --- | --- |
| *Using Niger specific* $\boldsymbol{R}_{\boldsymbol{t}}$ *estimates* | | | | | | | | |
| Key outcome | **Unmitigated** | **20% distancing** | **50% distancing** | **80% shielding** | | **80% shielding + 20% distancing** | | **80% shielding + 50% distancing** |
| Symptomatic cases | 3,270,000 (30,000 to 4,980,000) | 30,000 (200 to 3,390,000) | 10,000 (200 to 260,000) | | 660,000 (200 to 4,170,000) | 30,000 (200 to 3,300,000) | 10,000 (200 to 260,000) | |
| Severe cases | 60,000 (300 to 110,000) | 400 (7 to 70,000) | 200 (7 to 4,000) | | 4,000 (7 to 70,000) | 400 (7 to 50,000) | 200 (7 to 3,000) | |
| Critical cases | 30,000 (100 to 50,000) | 200 (4 to 30,000) | 100 (4 to 2,000) | | 2,000 (5 to 30,000) | 200 (5 to 20,000) | 90 (5 to 1,000) | |
| Deaths | 20,000 (70 to 30,000) | 100 (2 to 20,000) | 60 (2 to 900) | | 800 (2 to 20,000) | 90 (2 to 10,000) | 60 (2 to 800) | |
| Symptomatic attack rate | 13.6% (0.1 to 20.7) | 0.1% (0 to 14.1) | 0.1% (0 to 1.1) | | 2.7% (0 to 17.3) | 0.1% (0 to 13.7) | 0.1% (0 to 1.1) | |
| Deaths per 1000 person-years | 0.7 (0 to 1.3) | 0 (0 to 0.8) | 0 (0 to 0) | | 0 (0 to 0.8) | 0 (0 to 0.6) | 0 (0 to 0) | |
| Epidemic peak (months) | 6 (4 to 12) | 8 (0 to 12) | 4 (0 to 12) | | 9 (0 to 12) | 8 (0 to 12) | 4 (0 to 12) | |
| Peak deaths | 300 (4 to 700) | 5 (1 to 300) | 4 (1 to 20) | | 40 (1 to 400) | 5 (1 to 300) | 4 (1 to 20) | |
| Peak non-ICU beds needed | 8,000 (40 to 20,000) | 70 (4 to 9,000) | 50 (4 to 500) | | 2,000 (4 to 10,000) | 70 (4 to 7,000) | 50 (4 to 300) | |
| Peak ICU beds needed | 4,000 (20 to 10,000) | 40 (2 to 5,000) | 30 (2 to 200) | | 900 (2 to 6,000) | 40 (2 to 4,000) | 30 (2 to 200) | |
| *Using global* $\boldsymbol{R}_{\boldsymbol{0}}$ *estimates* | | | | | | | | |
| Key outcome | **Unmitigated** | **20% distancing** | **50% distancing** | **80% shielding** | | **80% shielding + 20% distancing** | | **80% shielding + 50% distancing** |
| Symptomatic cases | 5,480,000 (2,980,000 to 6,840,000) | 3,940,000 (6,000 to 5,560,000) | 1,550,000 (6,000 to 3,980,000) | 4,660,000 (50,000 to 6,060,000) | | 3,850,000 (7,000 to 5,410,000) | | 1,460,000 (7,000 to 3,860,000) |
| Severe cases | 130,000 (50,000 to 190,000) | 80,000 (100 to 140,000) | 30,000 (90 to 90,000) | 80,000 (400 to 120,000) | | 60,000 (100 to 100,000) | | 20,000 (100 to 70,000) |
| Critical cases | 60,000 (20,000 to 80,000) | 40,000 (40 to 60,000) | 10,000 (40 to 40,000) | 30,000 (200 to 50,000) | | 30,000 (50 to 40,000) | | 8,000 (40 to 30,000) |
| Deaths | 30,000 (10,000 to 50,000) | 20,000 (20 to 40,000) | 7,000 (20 to 30,000) | 20,000 (80 to 30,000) | | 20,000 (30 to 30,000) | | 5,000 (30 to 20,000) |
| Symptomatic attack rate | 22.7% (12.3 to 28.4) | 16.4% (0 to 23.1) | 6.4% (0 to 16.5) | 19.3% (0.2 to 25.1) | | 16% (0 to 22.5) | | 6.1% (0 to 16) |
| Deaths per 1000 person-years | 1.4 (0.6 to 2.1) | 0.9 (0 to 1.5) | 0.3 (0 to 1.1) | 0.9 (0 to 1.3) | | 0.7 (0 to 1.2) | | 0.2 (0 to 0.8) |
| Epidemic peak (months) | 4 (3 to 7) | 5 (4 to 7) | 6 (4 to 11) | 7 (5 to 12) | | 5 (4 to 7) | | 6 (4 to 11) |
| Peak deaths | 900 (200 to 2,000) | 400 (2 to 1,000) | 100 (2 to 500) | 500 (4 to 1,000) | | 300 (2 to 800) | | 80 (2 to 400) |
| Peak non-ICU beds needed | 30,000 (6,000 to 50,000) | 10,000 (20 to 30,000) | 3,000 (20 to 10,000) | 20,000 (100 to 30,000) | | 10,000 (20 to 20,000) | | 2,000 (20 to 10,000) |
| Peak ICU beds needed | 10,000 (3,000 to 30,000) | 7,000 (10 to 20,000) | 1,000 (10 to 7,000) | 8,000 (50 to 20,000) | | 5,000 (10 to 10,000) | | 1,000 (10 to 5,000) |

| Nigeria | | | | | | | |
| --- | --- | --- | --- | --- | --- | --- | --- |
| *Using Nigeria specific* $\boldsymbol{R}_{\boldsymbol{t}}$ *estimates* | | | | | | | |
| Key outcome | **Unmitigated** | | **20% distancing** | **50% distancing** | **80% shielding** | **80% shielding + 20% distancing** | **80% shielding + 50% distancing** |
| Symptomatic cases | 35,570,000 (5,000 to 53,270,000) | 70,000 (300 to 35,600,000) | | 60,000 (300 to 550,000) | 180,000 (200 to 44,210,000) | 70,000 (200 to 34,220,000) | 60,000 (200 to 440,000) |
| Severe cases | 1,020,000 (100 to 1,740,000) | 2,000 (9 to 1,030,000) | | 1,000 (9 to 10,000) | 2,000 (7 to 1,010,000) | 2,000 (7 to 720,000) | 1,000 (7 to 9,000) |
| Critical cases | 440,000 (40 to 750,000) | 800 (4 to 440,000) | | 600 (4 to 6,000) | 1,000 (3 to 430,000) | 700 (3 to 310,000) | 600 (3 to 4,000) |
| Deaths | 270,000 (30 to 460,000) | 500 (2 to 270,000) | | 400 (2 to 4,000) | 600 (3 to 270,000) | 400 (3 to 190,000) | 400 (3 to 2,000) |
| Symptomatic attack rate | 17.5% (0 to 26.3) | 0% (0 to 17.5) | | 0% (0 to 0.3) | 0.1% (0 to 21.8) | 0% (0 to 16.9) | 0% (0 to 0.2) |
| Deaths per 1000 person-years | 1.3 (0 to 2.3) | 0 (0 to 1.3) | | 0 (0 to 0) | 0 (0 to 1.3) | 0 (0 to 0.9) | 0 (0 to 0) |
| Epidemic peak (months) | 8 (5 to 12) | 8 (0 to 12) | | 5 (0 to 12) | 10 (0 to 12) | 8 (0 to 12) | 5 (0 to 12) |
| Peak deaths | 4,000 (2 to 10,000) | 10 (1 to 5,000) | | 10 (1 to 50) | 20 (1 to 6,000) | 10 (1 to 3,000) | 10 (1 to 40) |
| Peak non-ICU beds needed | 120,000 (10 to 330,000) | 300 (2 to 130,000) | | 300 (2 to 1,000) | 500 (3 to 170,000) | 300 (3 to 90,000) | 300 (3 to 1,000) |
| Peak ICU beds needed | 60,000 (7 to 180,000) | 100 (1 to 70,000) | | 100 (1 to 600) | 300 (2 to 90,000) | 200 (2 to 50,000) | 200 (2 to 500) |
| *Using global* $\boldsymbol{R}_{\boldsymbol{0}}$ *estimates* | | | | | | | |
| Key outcome | **Unmitigated** | | **20% distancing** | **50% distancing** | **80% shielding** | **80% shielding + 20% distancing** | **80% shielding + 50% distancing** |
| Symptomatic cases | 60,200,000 (35,920,000 to 69,730,000) | | 46,100,000 (40,000 to 60,510,000) | 20,750,000 (40,000 to 46,680,000) | 51,850,000 (100,000 to 62,930,000) | 44,260,000 (30,000 to 58,070,000) | 19,220,000 (30,000 to 44,560,000) |
| Severe cases | 2,090,000 (1,030,000 to 2,670,000) | | 1,480,000 (1,000 to 2,170,000) | 570,000 (1,000 to 1,650,000) | 1,260,000 (1,000 to 1,710,000) | 1,050,000 (800 to 1,540,000) | 370,000 (700 to 1,140,000) |
| Critical cases | 890,000 (440,000 to 1,150,000) | | 630,000 (500 to 930,000) | 240,000 (400 to 710,000) | 540,000 (600 to 740,000) | 450,000 (300 to 660,000) | 160,000 (300 to 490,000) |
| Deaths | 560,000 (270,000 to 710,000) | | 390,000 (300 to 580,000) | 140,000 (200 to 440,000) | 340,000 (300 to 460,000) | 280,000 (200 to 410,000) | 90,000 (200 to 300,000) |
| Symptomatic attack rate | 29.7% (17.7 to 34.4) | | 22.7% (0 to 29.8) | 10.2% (0 to 23) | 25.6% (0.1 to 31) | 21.8% (0 to 28.6) | 9.5% (0 to 22) |
| Deaths per 1000 person-years | 2.7 (1.4 to 3.5) | | 1.9 (0 to 2.9) | 0.7 (0 to 2.2) | 1.7 (0 to 2.3) | 1.4 (0 to 2) | 0.4 (0 to 1.5) |
| Epidemic peak (months) | 4 (3 to 8) | | 6 (5 to 8) | 6 (5 to 11) | 7 (5 to 12) | 6 (5 to 8) | 6 (5 to 11) |
| Peak deaths | 20,000 (4,000 to 30,000) | | 8,000 (9 to 20,000) | 2,000 (9 to 8,000) | 8,000 (9 to 20,000) | 6,000 (8 to 10,000) | 1,000 (8 to 6,000) |
| Peak non-ICU beds needed | 480,000 (120,000 to 800,000) | | 240,000 (200 to 500,000) | 60,000 (200 to 250,000) | 250,000 (300 to 460,000) | 170,000 (100 to 360,000) | 40,000 (100 to 170,000) |
| Peak ICU beds needed | 250,000 (70,000 to 420,000) | | 130,000 (100 to 270,000) | 30,000 (100 to 130,000) | 130,000 (100 to 240,000) | 90,000 (80 to 190,000) | 20,000 (80 to 90,000) |

| Mauritius | | | | | | |
| --- | --- | --- | --- | --- | --- | --- |
| *Using a distribution of* $\boldsymbol{R}_{\boldsymbol{t}}$ *estimates in African countries* | | | | | | |
| Key outcome | **Unmitigated** | **20% distancing** | **50% distancing** | **80% shielding** | **80% shielding + 20% distancing** | **80% shielding + 50% distancing** |
| Symptomatic cases | 380,000 (5,000 to 610,000) | 7,000 (200 to 540,000) | 2,000 (200 to 450,000) | 140,000 (100 to 500,000) | 3,000 (100 to 470,000) | 1,000 (100 to 390,000) |
| Severe cases | 30,000 (400 to 60,000) | 400 (10 to 50,000) | 100 (10 to 40,000) | 5,000 (9 to 40,000) | 200 (9 to 30,000) | 100 (9 to 30,000) |
| Critical cases | 10,000 (100 to 30,000) | 200 (6 to 20,000) | 50 (6 to 20,000) | 2,000 (3 to 20,000) | 70 (3 to 10,000) | 40 (3 to 10,000) |
| Deaths | 8,000 (90 to 20,000) | 90 (3 to 10,000) | 30 (3 to 10,000) | 1,000 (1 to 9,000) | 40 (1 to 9,000) | 30 (1 to 7,000) |
| Symptomatic attack rate | 29.1% (0.4 to 46.6) | 0.5% (0 to 41.7) | 0.1% (0 to 34.9) | 10.8% (0 to 38.6) | 0.3% (0 to 36.4) | 0.1% (0 to 30.3) |
| Deaths per 1000 person-years | 6.5 (0.1 to 12.8) | 0.1 (0 to 10.9) | 0 (0 to 8.9) | 0.9 (0 to 7.2) | 0 (0 to 6.6) | 0 (0 to 5.3) |
| Epidemic peak (months) | 5 (2 to 12) | 4 (0 to 12) | 2 (0 to 12) | 7 (0 to 12) | 4 (0 to 12) | 2 (0 to 12) |
| Peak deaths | 200 (3 to 700) | 4 (1 to 400) | 3 (1 to 300) | 40 (1 to 300) | 3 (1 to 300) | 2 (1 to 200) |
| Peak non-ICU beds needed | 4,000 (30 to 20,000) | 50 (4 to 10,000) | 20 (4 to 7,000) | 1,000 (3 to 10,000) | 30 (3 to 8,000) | 20 (3 to 4,000) |
| Peak ICU beds needed | 2,000 (20 to 10,000) | 30 (2 to 7,000) | 10 (2 to 4,000) | 600 (2 to 5,000) | 20 (2 to 4,000) | 10 (2 to 2,000) |
| *Using global* $\boldsymbol{R}_{\boldsymbol{0}}$ *estimates* | | | | | | |
| Key outcome | **Unmitigated** | **20% distancing** | **50% distancing** | **80% shielding** | **80% shielding + 20% distancing** | **80% shielding + 50% distancing** |
| Symptomatic cases | 540,000 (360,000 to 610,000) | 430,000 (900 to 540,000) | 270,000 (800 to 450,000) | 430,000 (1,000 to 500,000) | 380,000 (900 to 470,000) | 240,000 (800 to 390,000) |
| Severe cases | 50,000 (30,000 to 60,000) | 40,000 (70 to 50,000) | 20,000 (60 to 40,000) | 30,000 (70 to 40,000) | 20,000 (60 to 30,000) | 10,000 (60 to 30,000) |
| Critical cases | 20,000 (10,000 to 30,000) | 20,000 (30 to 20,000) | 10,000 (30 to 20,000) | 10,000 (30 to 20,000) | 10,000 (20 to 10,000) | 6,000 (20 to 10,000) |
| Deaths | 10,000 (8,000 to 20,000) | 10,000 (20 to 10,000) | 6,000 (20 to 10,000) | 7,000 (20 to 9,000) | 6,000 (20 to 9,000) | 4,000 (10 to 7,000) |
| Symptomatic attack rate | 41.7% (27.7 to 46.6) | 33.3% (0.1 to 41.5) | 21.1% (0.1 to 34.6) | 33.1% (0.1 to 38.5) | 29.2% (0.1 to 36.3) | 18.2% (0.1 to 30) |
| Deaths per 1000 person-years | 10.6 (6.1 to 12.9) | 7.9 (0 to 10.8) | 4.9 (0 to 8.8) | 5.6 (0 to 7.2) | 4.8 (0 to 6.7) | 2.9 (0 to 5.4) |
| Epidemic peak (months) | 3 (2 to 5) | 4 (2 to 12) | 5 (2 to 8) | 5 (2 to 12) | 4 (2 to 12) | 5 (2 to 8) |
| Peak deaths | 400 (100 to 700) | 200 (2 to 500) | 80 (2 to 300) | 200 (2 to 300) | 100 (2 to 300) | 50 (1 to 200) |
| Peak non-ICU beds needed | 10,000 (4,000 to 20,000) | 6,000 (20 to 10,000) | 2,000 (10 to 7,000) | 6,000 (20 to 10,000) | 4,000 (10 to 8,000) | 1,000 (10 to 4,000) |
| Peak ICU beds needed | 7,000 (2,000 to 10,000) | 3,000 (8 to 7,000) | 1,000 (7 to 4,000) | 3,000 (8 to 5,000) | 2,000 (7 to 4,000) | 600 (6 to 2,000) |


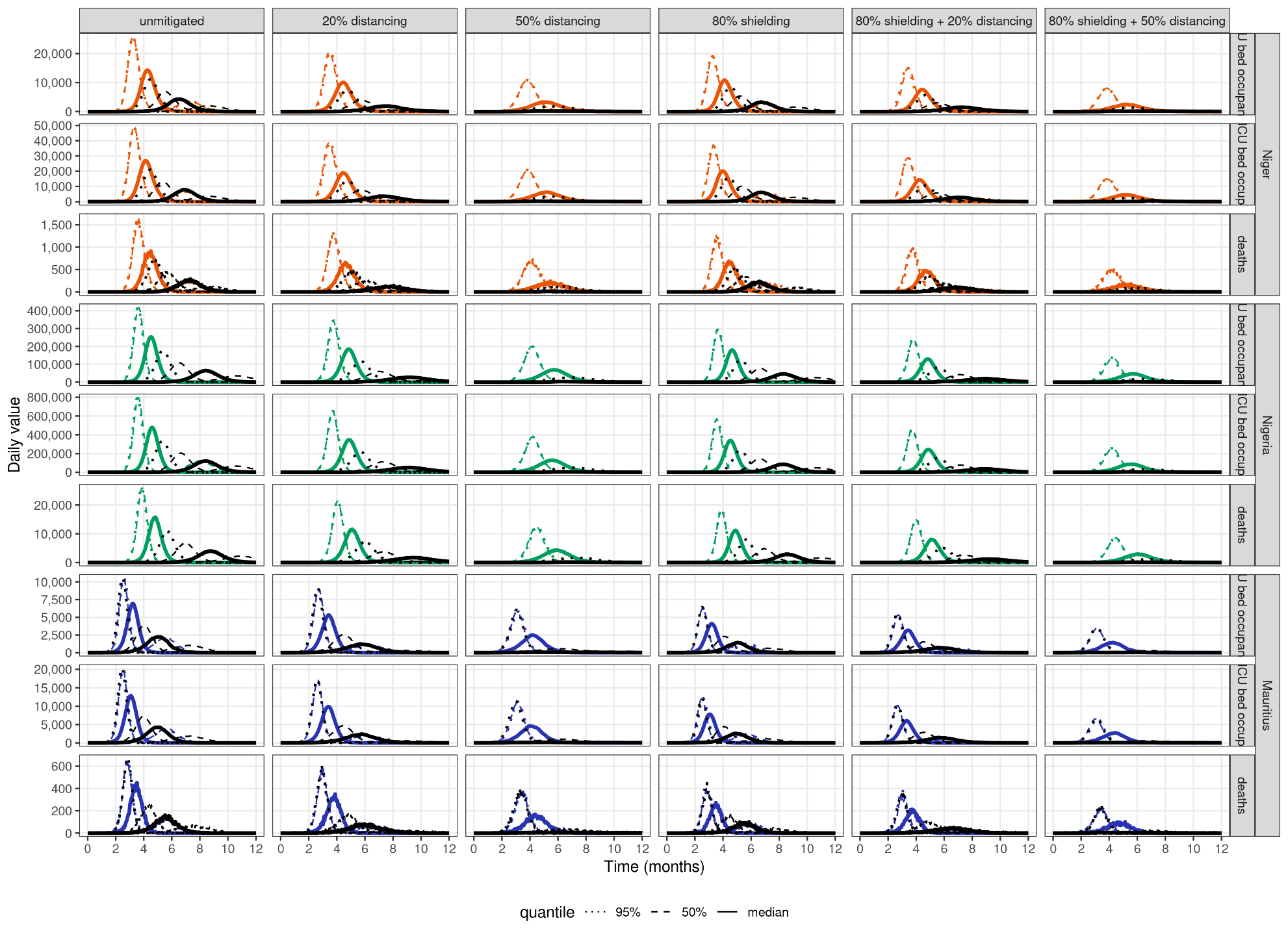
 **Figure S4. Bed demand and deaths during the first 12 months of the epidemic, under different strategies but in the absence of self-isolation.** Lines show the median, 95%, and 50% quantiles of total number of the corresponding outcome. Black lines show estimates for scenarios using country specific $R_{t}$ estimates, while coloured lines show estimates for scenarios using global $R_{\boldsymbol{0}}$ estimates.

#### **Table S4. Key outcomes during the first 12 months of the epidemic, under different strategies but in the absence of self-isolation.**

| Niger | | | | | | | | |
| --- | --- | --- | --- | --- | --- | --- | --- | --- |
| *Using Niger specific* $\boldsymbol{R}_{\boldsymbol{t}}$ *estimates* | | | | | | | | |
| Key outcome | **Unmitigated** | **20% distancing** | **50% distancing** | **80% shielding** | | **80% shielding + 20% distancing** | | **80% shielding + 50% distancing** |
| Symptomatic cases | 3,270,000 (30,000 to 4,980,000) | 2,280,000 (30,000 to 4,140,000) | 310,000 (30,000 to 2,170,000) | | 3,200,000 (10,000 to 4,860,000) | 2,230,000 (10,000 to 4,040,000) | 320,000 (10,000 to 2,100,000) | |
| Severe cases | 60,000 (300 to 110,000) | 40,000 (300 to 90,000) | 6,000 (300 to 50,000) | | 50,000 (200 to 90,000) | 30,000 (200 to 70,000) | 5,000 (200 to 30,000) | |
| Critical cases | 30,000 (100 to 50,000) | 20,000 (100 to 40,000) | 2,000 (100 to 20,000) | | 20,000 (60 to 40,000) | 10,000 (60 to 30,000) | 2,000 (60 to 10,000) | |
| Deaths | 20,000 (70 to 30,000) | 10,000 (70 to 20,000) | 1,000 (70 to 10,000) | | 10,000 (40 to 20,000) | 9,000 (40 to 20,000) | 1,000 (40 to 9,000) | |
| Symptomatic attack rate | 13.6% (0.1 to 20.7) | 9.5% (0.1 to 17.2) | 1.3% (0.1 to 9) | | 13.3% (0.1 to 20.1) | 9.3% (0.1 to 16.8) | 1.3% (0.1 to 8.7) | |
| Deaths per 1000 person-years | 0.7 (0 to 1.3) | 0.5 (0 to 1) | 0.1 (0 to 0.5) | | 0.5 (0 to 1) | 0.4 (0 to 0.8) | 0 (0 to 0.4) | |
| Epidemic peak (months) | 6 (4 to 12) | 6 (4 to 12) | 5 (4 to 12) | | 6 (4 to 12) | 7 (4 to 12) | 5 (4 to 12) | |
| Peak deaths | 300 (4 to 700) | 100 (4 to 500) | 20 (4 to 100) | | 200 (3 to 600) | 100 (3 to 400) | 20 (3 to 100) | |
| Peak non-ICU beds needed | 8,000 (40 to 20,000) | 4,000 (40 to 10,000) | 400 (40 to 4,000) | | 6,000 (30 to 20,000) | 3,000 (30 to 10,000) | 400 (30 to 3,000) | |
| Peak ICU beds needed | 4,000 (20 to 10,000) | 2,000 (20 to 8,000) | 200 (20 to 2,000) | | 3,000 (10 to 9,000) | 2,000 (10 to 6,000) | 200 (10 to 1,000) | |
| *Using global* $\boldsymbol{R}_{\boldsymbol{0}}$ *estimates* | | | | | | | | |
| Key outcome | **Unmitigated** | **20% distancing** | **50% distancing** | **80% shielding** | | **80% shielding + 20% distancing** | | **80% shielding + 50% distancing** |
| Symptomatic cases | 5,480,000 (2,980,000 to 6,840,000) | 4,690,000 (1,960,000 to 6,250,000) | 2,840,000 (220,000 to 4,820,000) | 5,330,000 (2,910,000 to 6,630,000) | | 4,560,000 (1,900,000 to 6,060,000) | | 2,740,000 (120,000 to 4,650,000) |
| Severe cases | 130,000 (50,000 to 190,000) | 110,000 (30,000 to 170,000) | 60,000 (4,000 to 120,000) | 100,000 (40,000 to 140,000) | | 80,000 (30,000 to 130,000) | | 50,000 (2,000 to 90,000) |
| Critical cases | 60,000 (20,000 to 80,000) | 50,000 (10,000 to 70,000) | 30,000 (2,000 to 50,000) | 40,000 (20,000 to 60,000) | | 40,000 (10,000 to 50,000) | | 20,000 (700 to 40,000) |
| Deaths | 30,000 (10,000 to 50,000) | 30,000 (9,000 to 40,000) | 20,000 (1,000 to 30,000) | 30,000 (10,000 to 40,000) | | 20,000 (7,000 to 30,000) | | 10,000 (400 to 20,000) |
| Symptomatic attack rate | 22.7% (12.3 to 28.4) | 19.5% (8.1 to 25.9) | 11.8% (0.9 to 20) | 22.1% (12.1 to 27.5) | | 18.9% (7.9 to 25.1) | | 11.4% (0.5 to 19.3) |
| Deaths per 1000 person-years | 1.4 (0.6 to 2.1) | 1.2 (0.4 to 1.9) | 0.7 (0 to 1.4) | 1.1 (0.5 to 1.6) | | 0.9 (0.3 to 1.4) | | 0.5 (0 to 1) |
| Epidemic peak (months) | 4 (3 to 7) | 4 (3 to 7) | 4 (3 to 5) | 4 (3 to 7) | | 4 (3 to 8) | | 4 (3 to 5) |
| Peak deaths | 900 (200 to 2,000) | 700 (100 to 1,000) | 200 (20 to 700) | 700 (200 to 1,000) | | 500 (80 to 1,000) | | 200 (9 to 500) |
| Peak non-ICU beds needed | 30,000 (6,000 to 50,000) | 20,000 (3,000 to 40,000) | 6,000 (300 to 20,000) | 20,000 (5,000 to 40,000) | | 10,000 (2,000 to 30,000) | | 5,000 (100 to 20,000) |
| Peak ICU beds needed | 10,000 (3,000 to 30,000) | 10,000 (1,000 to 20,000) | 3,000 (200 to 10,000) | 10,000 (3,000 to 20,000) | | 8,000 (1,000 to 20,000) | | 2,000 (80 to 8,000) |

| Nigeria | | | | | | | |
| --- | --- | --- | --- | --- | --- | --- | --- |
| *Using Nigeria specific* $\boldsymbol{R}_{\boldsymbol{t}}$ *estimates* | | | | | | | |
| Key outcome | **Unmitigated** | | **20% distancing** | **50% distancing** | **80% shielding** | **80% shielding + 20% distancing** | **80% shielding + 50% distancing** |
| Symptomatic cases | 35,570,000 (5,000 to 53,270,000) | 23,540,000 (5,000 to 45,070,000) | | 2,250,000 (5,000 to 23,150,000) | 34,220,000 (4,000 to 51,200,000) | 22,700,000 (4,000 to 43,200,000) | 2,600,000 (4,000 to 22,020,000) |
| Severe cases | 1,020,000 (100 to 1,740,000) | 650,000 (100 to 1,440,000) | | 60,000 (100 to 730,000) | 740,000 (80 to 1,250,000) | 470,000 (80 to 1,020,000) | 50,000 (80 to 500,000) |
| Critical cases | 440,000 (40 to 750,000) | 280,000 (40 to 620,000) | | 30,000 (40 to 310,000) | 320,000 (40 to 540,000) | 200,000 (40 to 440,000) | 20,000 (40 to 210,000) |
| Deaths | 270,000 (30 to 460,000) | 170,000 (30 to 380,000) | | 20,000 (30 to 190,000) | 200,000 (20 to 330,000) | 120,000 (20 to 270,000) | 10,000 (20 to 130,000) |
| Symptomatic attack rate | 17.5% (0 to 26.3) | 11.6% (0 to 22.2) | | 1.1% (0 to 11.4) | 16.9% (0 to 25.2) | 11.2% (0 to 21.3) | 1.3% (0 to 10.9) |
| Deaths per 1000 person-years | 1.3 (0 to 2.3) | 0.8 (0 to 1.9) | | 0.1 (0 to 1) | 1 (0 to 1.6) | 0.6 (0 to 1.3) | 0.1 (0 to 0.7) |
| Epidemic peak (months) | 8 (5 to 12) | 8 (5 to 12) | | 7 (5 to 12) | 8 (5 to 12) | 8 (5 to 12) | 7 (5 to 12) |
| Peak deaths | 4,000 (2 to 10,000) | 2,000 (2 to 7,000) | | 200 (2 to 2,000) | 3,000 (2 to 8,000) | 1,000 (2 to 5,000) | 200 (2 to 1,000) |
| Peak non-ICU beds needed | 120,000 (10 to 330,000) | 50,000 (10 to 220,000) | | 5,000 (10 to 50,000) | 90,000 (9 to 240,000) | 40,000 (9 to 150,000) | 5,000 (9 to 30,000) |
| Peak ICU beds needed | 60,000 (7 to 180,000) | 30,000 (7 to 120,000) | | 3,000 (7 to 30,000) | 50,000 (5 to 130,000) | 20,000 (5 to 80,000) | 3,000 (5 to 20,000) |
| *Using global* $\boldsymbol{R}_{\boldsymbol{0}}$ *estimates* | | | | | | | |
| Key outcome | **Unmitigated** | | **20% distancing** | **50% distancing** | **80% shielding** | **80% shielding + 20% distancing** | **80% shielding + 50% distancing** |
| Symptomatic cases | 60,200,000 (35,920,000 to 69,730,000) | | 53,630,000 (23,900,000 to 65,860,000) | 35,690,000 (2,270,000 to 54,620,000) | 57,830,000 (34,540,000 to 66,930,000) | 51,410,000 (22,710,000 to 63,110,000) | 33,900,000 (1,500,000 to 52,070,000) |
| Severe cases | 2,090,000 (1,030,000 to 2,670,000) | | 1,820,000 (660,000 to 2,490,000) | 1,200,000 (60,000 to 2,030,000) | 1,500,000 (740,000 to 1,930,000) | 1,290,000 (460,000 to 1,770,000) | 820,000 (30,000 to 1,400,000) |
| Critical cases | 890,000 (440,000 to 1,150,000) | | 780,000 (280,000 to 1,070,000) | 510,000 (30,000 to 870,000) | 640,000 (320,000 to 830,000) | 550,000 (200,000 to 760,000) | 350,000 (10,000 to 600,000) |
| Deaths | 560,000 (270,000 to 710,000) | | 490,000 (170,000 to 660,000) | 320,000 (20,000 to 540,000) | 400,000 (200,000 to 510,000) | 340,000 (120,000 to 470,000) | 220,000 (8,000 to 370,000) |
| Symptomatic attack rate | 29.7% (17.7 to 34.4) | | 26.4% (11.8 to 32.5) | 17.6% (1.1 to 26.9) | 28.5% (17 to 33) | 25.3% (11.2 to 31.1) | 16.7% (0.7 to 25.7) |
| Deaths per 1000 person-years | 2.7 (1.4 to 3.5) | | 2.4 (0.9 to 3.3) | 1.6 (0.1 to 2.7) | 2 (1 to 2.5) | 1.7 (0.6 to 2.3) | 1.1 (0 to 1.8) |
| Epidemic peak (months) | 4 (3 to 8) | | 4 (3 to 9) | 5 (4 to 6) | 4 (3 to 8) | 4 (3 to 9) | 5 (4 to 6) |
| Peak deaths | 20,000 (4,000 to 30,000) | | 10,000 (2,000 to 20,000) | 4,000 (200 to 10,000) | 10,000 (3,000 to 20,000) | 8,000 (1,000 to 20,000) | 3,000 (100 to 9,000) |
| Peak non-ICU beds needed | 480,000 (120,000 to 800,000) | | 350,000 (50,000 to 660,000) | 130,000 (5,000 to 380,000) | 340,000 (90,000 to 570,000) | 240,000 (40,000 to 460,000) | 90,000 (3,000 to 260,000) |
| Peak ICU beds needed | 250,000 (70,000 to 420,000) | | 180,000 (30,000 to 350,000) | 70,000 (3,000 to 200,000) | 180,000 (50,000 to 300,000) | 130,000 (20,000 to 240,000) | 50,000 (1,000 to 140,000) |

| Mauritius | | | | | | |
| --- | --- | --- | --- | --- | --- | --- |
| *Using a distribution of* $\boldsymbol{R}_{\boldsymbol{t}}$ *estimates in African countries* | | | | | | |
| Key outcome | **Unmitigated** | **20% distancing** | **50% distancing** | **80% shielding** | **80% shielding + 20% distancing** | **80% shielding + 50% distancing** |
| Symptomatic cases | 380,000 (5,000 to 610,000) | 290,000 (5,000 to 590,000) | 50,000 (5,000 to 520,000) | 330,000 (6,000 to 530,000) | 250,000 (6,000 to 510,000) | 40,000 (3,000 to 450,000) |
| Severe cases | 30,000 (400 to 60,000) | 20,000 (400 to 60,000) | 4,000 (400 to 50,000) | 20,000 (400 to 40,000) | 10,000 (300 to 40,000) | 2,000 (200 to 30,000) |
| Critical cases | 10,000 (100 to 30,000) | 10,000 (100 to 30,000) | 2,000 (100 to 20,000) | 8,000 (200 to 20,000) | 6,000 (200 to 20,000) | 900 (80 to 10,000) |
| Deaths | 8,000 (90 to 20,000) | 6,000 (90 to 20,000) | 1,000 (90 to 10,000) | 5,000 (100 to 10,000) | 4,000 (90 to 10,000) | 500 (60 to 9,000) |
| Symptomatic attack rate | 29.1% (0.4 to 46.6) | 22.2% (0.4 to 45) | 3.6% (0.4 to 40.2) | 25.6% (0.5 to 40.8) | 19.5% (0.4 to 39.4) | 2.9% (0.2 to 34.8) |
| Deaths per 1000 person-years | 6.5 (0.1 to 12.8) | 4.9 (0.1 to 12.3) | 0.8 (0.1 to 10.7) | 4 (0.1 to 8.1) | 3 (0.1 to 7.6) | 0.4 (0 to 6.6) |
| Epidemic peak (months) | 5 (2 to 12) | 5 (2 to 11) | 5 (2 to 11) | 5 (2 to 12) | 5 (2 to 12) | 5 (2 to 12) |
| Peak deaths | 200 (3 to 700) | 90 (3 to 600) | 10 (3 to 400) | 100 (3 to 400) | 60 (3 to 400) | 8 (2 to 200) |
| Peak non-ICU beds needed | 4,000 (30 to 20,000) | 2,000 (30 to 20,000) | 100 (30 to 10,000) | 3,000 (40 to 10,000) | 1,000 (30 to 10,000) | 100 (20 to 7,000) |
| Peak ICU beds needed | 2,000 (20 to 10,000) | 1,000 (20 to 9,000) | 80 (20 to 6,000) | 1,000 (20 to 7,000) | 700 (20 to 6,000) | 60 (20 to 4,000) |
| *Using global* $\boldsymbol{R}_{\boldsymbol{0}}$ *estimates* | | | | | | |
| Key outcome | **Unmitigated** | **20% distancing** | **50% distancing** | **80% shielding** | **80% shielding + 20% distancing** | **80% shielding + 50% distancing** |
| Symptomatic cases | 540,000 (360,000 to 610,000) | 500,000 (270,000 to 580,000) | 380,000 (30,000 to 520,000) | 470,000 (320,000 to 530,000) | 440,000 (230,000 to 510,000) | 330,000 (20,000 to 450,000) |
| Severe cases | 50,000 (30,000 to 60,000) | 50,000 (20,000 to 60,000) | 40,000 (2,000 to 50,000) | 30,000 (20,000 to 40,000) | 30,000 (10,000 to 40,000) | 20,000 (1,000 to 30,000) |
| Critical cases | 20,000 (10,000 to 30,000) | 20,000 (9,000 to 30,000) | 20,000 (1,000 to 20,000) | 10,000 (8,000 to 20,000) | 10,000 (6,000 to 20,000) | 9,000 (400 to 10,000) |
| Deaths | 10,000 (8,000 to 20,000) | 10,000 (6,000 to 20,000) | 9,000 (600 to 10,000) | 9,000 (5,000 to 10,000) | 8,000 (4,000 to 10,000) | 6,000 (300 to 8,000) |
| Symptomatic attack rate | 41.7% (27.7 to 46.6) | 38.5% (20.5 to 44.9) | 29.4% (2.2 to 39.9) | 36.5% (24.4 to 40.8) | 33.6% (17.9 to 39.2) | 25.3% (1.3 to 34.5) |
| Deaths per 1000 person-years | 10.6 (6.1 to 12.9) | 9.6 (4.5 to 12.2) | 7.2 (0.4 to 10.7) | 6.6 (3.8 to 8.2) | 5.9 (2.7 to 7.6) | 4.3 (0.2 to 6.5) |
| Epidemic peak (months) | 3 (2 to 5) | 3 (2 to 6) | 3 (2 to 5) | 3 (2 to 5) | 3 (2 to 6) | 3 (2 to 4) |
| Peak deaths | 400 (100 to 700) | 300 (70 to 600) | 200 (7 to 400) | 300 (80 to 400) | 200 (40 to 400) | 100 (4 to 200) |
| Peak non-ICU beds needed | 10,000 (4,000 to 20,000) | 10,000 (2,000 to 20,000) | 5,000 (100 to 10,000) | 8,000 (2,000 to 10,000) | 6,000 (1,000 to 10,000) | 3,000 (50 to 7,000) |
| Peak ICU beds needed | 7,000 (2,000 to 10,000) | 5,000 (1,000 to 9,000) | 2,000 (60 to 6,000) | 4,000 (1,000 to 6,000) | 3,000 (600 to 5,000) | 1,000 (30 to 4,000) |

# Sensitivity analyses

## Results using empirical contact matrices

We replicated the analysis by substituting the synthetic contact matrices with a combination of three empirical contact matrices (Kenya [11], Uganda [10] and Zimbabwe [12]), selected because at the time of analysis they were the sole published matrices from low-income settings in Sub-Saharan Africa with sufficient detail on the age and location of contacts. As these data came from household sample surveys, in each model run we randomly selected one of the three and used the socialmixr [21] package to select a bootstrap contact matrix for that run by sampling with replacement from the set of contact survey observations.

Generally, empirical matrix results are subject to considerably uncertainty due to the bootstrapping procedure above. Figure S5 shows the effects of self-isolation and physical distancing using these empirical matrices. These suggest a slightly higher effect of self-isolation compared to synthetic matrix results; by contrast, general distancing would require slightly higher reductions in contacts to achieve comparable effects.


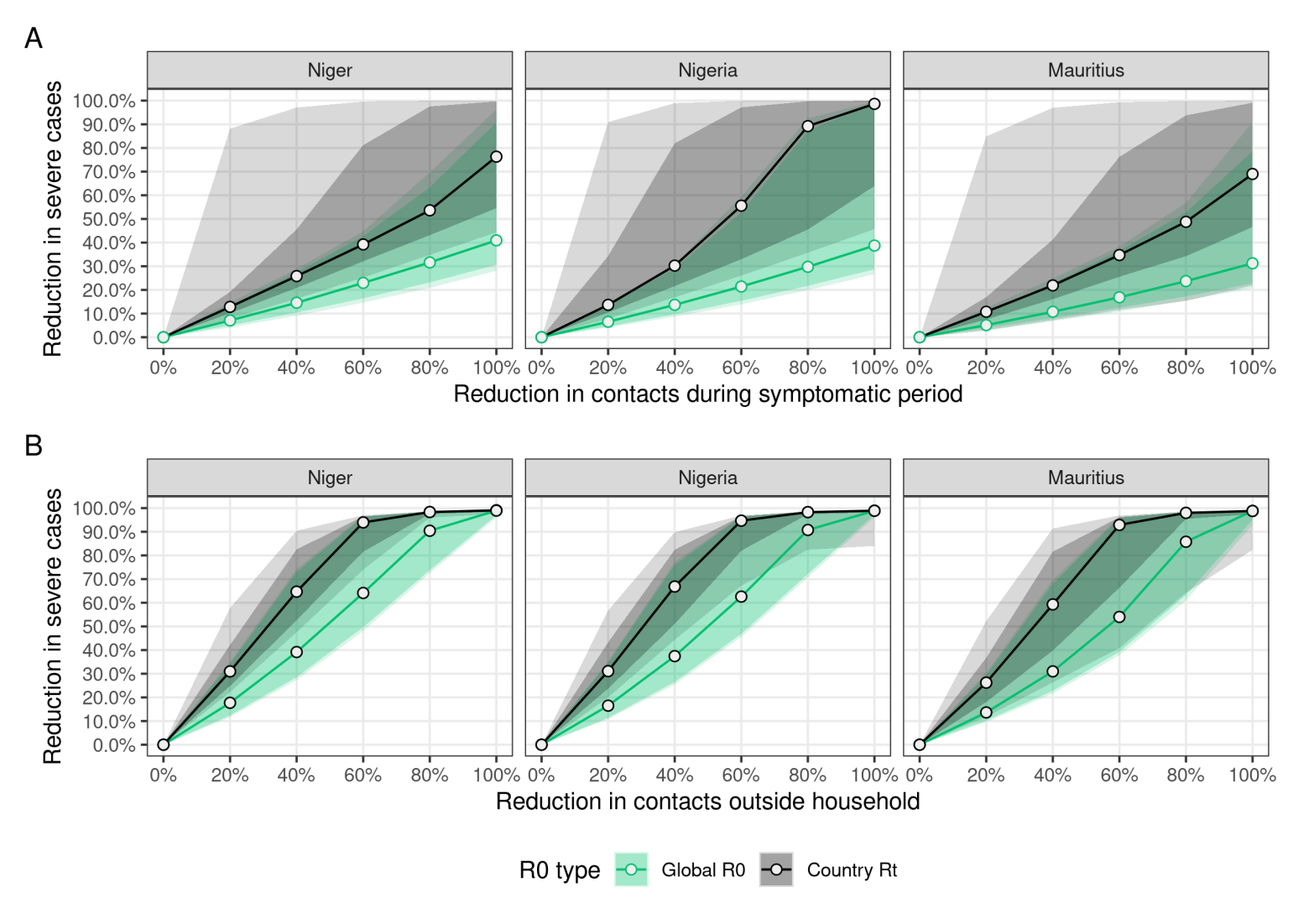


#### **Figure S5: Estimated reduction in severe cases following A) self-isolation of symptomatic individuals and B) population-wide physical distancing, by country, using empirical contact matrix data.** Medians (circles), 95% (light-shaded area) and 50% (dark-shaded area) quantiles for the percentage reduction in severe cases (patients requiring hospitalisation) during the first 12 months of the epidemic for different levels of compliance, for each country, across all model runs in each scenario. Black areas show estimates for scenarios using country specific $R_{t}$ estimates, while coloured areas show estimates for scenarios using global $R_{\boldsymbol{0}}$ estimates.

Figure S6 shows the effects of varying levels of shielding coverage and stringency. The salient difference between these results and those obtained under synthetic matrices is the very low or negative effect of shielding if contacts among shielded people are quadrupled. Empirical contact matrices feature a much higher intensity of contact in older age groups than synthetic ones (data not shown), such that the epidemic would be self-sustaining within the shielded group even after contacts with unshielded people are reduced or completely eliminated, an intervention which in our model is triggered by crossing an incidence threshold: in this quadrupling scenario, shielding is mostly harmful. However, note that our model considers the shielded group as a large single compartment, whereas in practice small numbers of shielded people would be living together, with presumably minimal contact among shielded residences.

Table S5 shows key outcomes over 12 months under the different strategies. Table S6 reports the relative reduction in key outcomes under these same strategies.

####
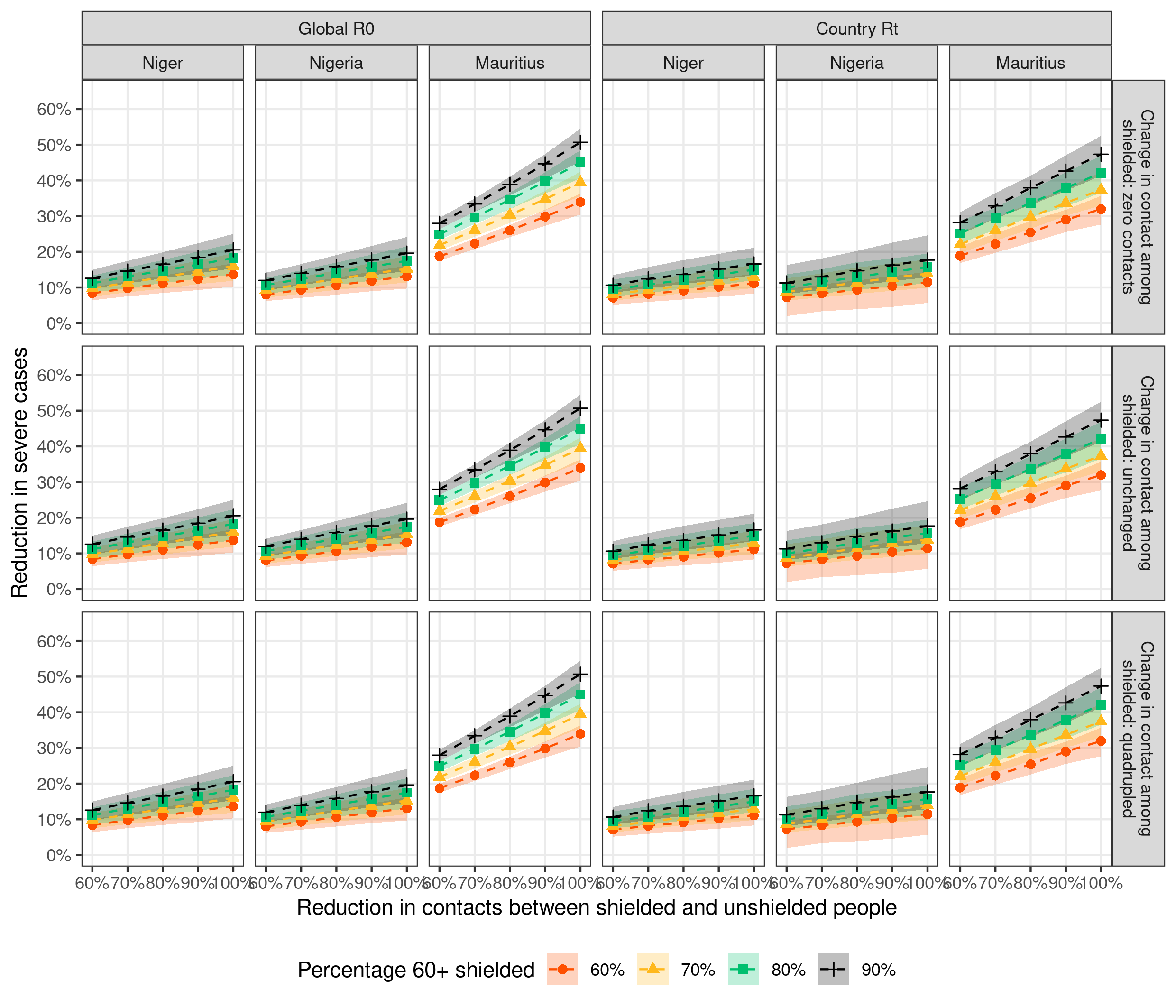
 **Figure S6: Estimated reduction in severe cases following shielding of high-risk individuals, by country with empirical contact matrix data.** Medians (dashed lines) and 95% quantiles (shaded areas) of the percentage reduction in severe cases during the first 12 months of the epidemic for different levels of reduction in contacts between shielded and unshielded people (x axis), different level of contacts among shielded people (facet rows), and for different percentages of people ≥ 60 years old being shielded (see legend) for each country, across all model runs in each scenario.

####

#### **Table S5. Key outcomes during the first 12 months of the epidemic, under different strategies, using empirical contact matrices.**

| Niger | | | | | | | | |
| --- | --- | --- | --- | --- | --- | --- | --- | --- |
| *Using Niger specific* $\boldsymbol{R}_{\boldsymbol{t}}$ *estimates* | | | | | | | | |
| Key outcome | **Unmitigated** | **20% distancing** | **50% distancing** | **80% shielding** | | **80% shielding + 20% distancing** | | **80% shielding + 50% distancing** |
| Symptomatic cases | 5,260,000 (90,000 to 6,850,000) | 2,620,000 (1,000 to 5,150,000) | 70,000 (1,000 to 2,890,000) | | 3,990,000 (1,000 to 5,950,000) | 2,520,000 (1,000 to 5,070,000) | 60,000 (1,000 to 2,870,000) | |
| Severe cases | 90,000 (800 to 140,000) | 30,000 (20 to 80,000) | 700 (20 to 40,000) | | 50,000 (20 to 100,000) | 30,000 (20 to 70,000) | 600 (20 to 30,000) | |
| Critical cases | 40,000 (300 to 60,000) | 10,000 (7 to 40,000) | 300 (7 to 20,000) | | 20,000 (7 to 40,000) | 10,000 (8 to 30,000) | 300 (7 to 10,000) | |
| Deaths | 20,000 (200 to 40,000) | 8,000 (6 to 20,000) | 200 (6 to 10,000) | | 10,000 (5 to 30,000) | 7,000 (6 to 20,000) | 200 (5 to 9,000) | |
| Symptomatic attack rate | 21.8% (0.4 to 28.4) | 10.9% (0 to 21.4) | 0.3% (0 to 12) | | 16.6% (0 to 24.7) | 10.5% (0 to 21.1) | 0.3% (0 to 11.9) | |
| Deaths per 1000 person-years | 1 (0 to 1.5) | 0.4 (0 to 0.9) | 0 (0 to 0.4) | | 0.6 (0 to 1.1) | 0.3 (0 to 0.8) | 0 (0 to 0.4) | |
| Epidemic peak (months) | 5 (3 to 12) | 7 (2 to 12) | 5 (2 to 11) | | 6 (1 to 12) | 7 (1 to 12) | 5 (1 to 11) | |
| Peak deaths | 400 (5 to 900) | 80 (1 to 400) | 4 (1 to 100) | | 200 (1 to 600) | 80 (1 to 300) | 5 (1 to 90) | |
| Peak non-ICU beds needed | 10,000 (100 to 30,000) | 2,000 (5 to 10,000) | 50 (5 to 2,000) | | 5,000 (4 to 20,000) | 2,000 (4 to 10,000) | 50 (3 to 2,000) | |
| Peak ICU beds needed | 6,000 (60 to 10,000) | 1,000 (3 to 6,000) | 30 (3 to 1,000) | | 3,000 (2 to 8,000) | 1,000 (2 to 5,000) | 30 (2 to 1,000) | |
| *Using global* $\boldsymbol{R}_{\boldsymbol{0}}$ *estimates* | | | | | | | | |
| Key outcome | **Unmitigated** | **20% distancing** | **50% distancing** | **80% shielding** | | **80% shielding + 20% distancing** | | **80% shielding + 50% distancing** |
| Symptomatic cases | 7,130,000 (4,740,000 to 7,970,000) | 5,680,000 (1,680,000 to 7,150,000) | 3,710,000 (30,000 to 5,860,000) | 6,340,000 (3,430,000 to 7,480,000) | | 5,570,000 (1,580,000 to 7,050,000) | | 3,640,000 (30,000 to 5,770,000) |
| Severe cases | 150,000 (70,000 to 200,000) | 100,000 (20,000 to 150,000) | 50,000 (300 to 100,000) | 110,000 (40,000 to 150,000) | | 80,000 (20,000 to 130,000) | | 40,000 (300 to 90,000) |
| Critical cases | 60,000 (30,000 to 90,000) | 40,000 (8,000 to 60,000) | 20,000 (100 to 40,000) | 50,000 (20,000 to 60,000) | | 40,000 (7,000 to 60,000) | | 20,000 (100 to 40,000) |
| Deaths | 40,000 (20,000 to 50,000) | 30,000 (5,000 to 40,000) | 10,000 (80 to 30,000) | 30,000 (10,000 to 40,000) | | 20,000 (4,000 to 30,000) | | 10,000 (70 to 20,000) |
| Symptomatic attack rate | 29.6% (19.7 to 33.1) | 23.6% (7 to 29.7) | 15.4% (0.1 to 24.3) | 26.3% (14.2 to 31) | | 23.1% (6.5 to 29.3) | | 15.1% (0.1 to 23.9) |
| Deaths per 1000 person-years | 1.7 (0.8 to 2.2) | 1.1 (0.2 to 1.7) | 0.5 (0 to 1.1) | 1.2 (0.5 to 1.6) | | 0.9 (0.2 to 1.4) | | 0.5 (0 to 1) |
| Epidemic peak (months) | 3 (2 to 6) | 4 (3 to 10) | 3 (3 to 5) | 3 (3 to 8) | | 4 (3 to 10) | | 3 (3 to 5) |
| Peak deaths | 1,000 (300 to 2,000) | 500 (50 to 1,000) | 200 (3 to 600) | 700 (100 to 1,000) | | 500 (50 to 1,000) | | 200 (3 to 500) |
| Peak non-ICU beds needed | 30,000 (8,000 to 60,000) | 10,000 (1,000 to 30,000) | 5,000 (30 to 20,000) | 20,000 (4,000 to 40,000) | | 10,000 (1,000 to 30,000) | | 4,000 (30 to 10,000) |
| Peak ICU beds needed | 20,000 (4,000 to 30,000) | 8,000 (700 to 20,000) | 2,000 (20 to 9,000) | 10,000 (2,000 to 20,000) | | 7,000 (600 to 20,000) | | 2,000 (20 to 8,000) |

| Nigeria | | | | | | | | |
| --- | --- | --- | --- | --- | --- | --- | --- | --- |
| *Using Nigeria specific* $\boldsymbol{R}_{\boldsymbol{t}}$ *estimates* | | | | | | | | |
| Key outcome | **Unmitigated** | **20% distancing** | **50% distancing** | **80% shielding** | | **80% shielding + 20% distancing** | | **80% shielding + 50% distancing** |
| Symptomatic cases | 41,930,000 (20,000 to 56,670,000) | 6,900,000 (500 to 40,170,000) | 150,000 (500 to 18,200,000) | | 28,050,000 (400 to 48,020,000) | 6,210,000 (400 to 39,300,000) | 140,000 (400 to 17,550,000) | |
| Severe cases | 770,000 (300 to 1,270,000) | 70,000 (10 to 730,000) | 2,000 (10 to 270,000) | | 410,000 (10 to 860,000) | 60,000 (10 to 640,000) | 2,000 (10 to 240,000) | |
| Critical cases | 330,000 (100 to 540,000) | 30,000 (4 to 310,000) | 900 (4 to 110,000) | | 170,000 (4 to 370,000) | 30,000 (4 to 270,000) | 800 (4 to 100,000) | |
| Deaths | 210,000 (70 to 340,000) | 20,000 (3 to 200,000) | 500 (3 to 70,000) | | 110,000 (3 to 230,000) | 20,000 (3 to 170,000) | 500 (3 to 60,000) | |
| Symptomatic attack rate | 20.7% (0 to 27.9) | 3.4% (0 to 19.8) | 0.1% (0 to 9) | | 13.8% (0 to 23.7) | 3.1% (0 to 19.4) | 0.1% (0 to 8.6) | |
| Deaths per 1000 person-years | 1 (0 to 1.7) | 0.1 (0 to 1) | 0 (0 to 0.3) | | 0.5 (0 to 1.1) | 0.1 (0 to 0.8) | 0 (0 to 0.3) | |
| Epidemic peak (months) | 7 (4 to 12) | 10 (1 to 12) | 6 (1 to 12) | | 9 (0 to 12) | 10 (0 to 12) | 6 (0 to 12) | |
| Peak deaths | 3,000 (3 to 8,000) | 300 (1 to 3,000) | 10 (1 to 500) | | 1,000 (1 to 5,000) | 300 (1 to 3,000) | 10 (1 to 500) | |
| Peak non-ICU beds needed | 90,000 (30 to 240,000) | 9,000 (3 to 90,000) | 200 (3 to 20,000) | | 40,000 (4 to 140,000) | 8,000 (4 to 80,000) | 200 (4 to 10,000) | |
| Peak ICU beds needed | 50,000 (10 to 130,000) | 5,000 (2 to 50,000) | 100 (2 to 8,000) | | 20,000 (2 to 70,000) | 4,000 (2 to 40,000) | 90 (2 to 7,000) | |
| *Using global* $\boldsymbol{R}_{\boldsymbol{0}}$ *estimates* | | | | | | | | |
| Key outcome | **Unmitigated** | **20% distancing** | **50% distancing** | **80% shielding** | | **80% shielding + 20% distancing** | | **80% shielding + 50% distancing** |
| Symptomatic cases | 62,330,000 (39,670,000 to 70,270,000) | 48,950,000 (6,530,000 to 62,500,000) | 30,990,000 (110,000 to 50,300,000) | 55,170,000 (26,970,000 to 65,470,000) | | 48,060,000 (5,580,000 to 61,220,000) | | 30,310,000 (100,000 to 49,410,000) |
| Severe cases | 1,480,000 (710,000 to 1,980,000) | 970,000 (70,000 to 1,500,000) | 510,000 (1,000 to 1,030,000) | 1,060,000 (380,000 to 1,460,000) | | 850,000 (60,000 to 1,280,000) | | 450,000 (1,000 to 890,000) |
| Critical cases | 630,000 (300,000 to 850,000) | 420,000 (30,000 to 640,000) | 220,000 (600 to 440,000) | 460,000 (160,000 to 630,000) | | 370,000 (20,000 to 550,000) | | 190,000 (500 to 380,000) |
| Deaths | 390,000 (190,000 to 530,000) | 260,000 (20,000 to 400,000) | 140,000 (400 to 270,000) | 280,000 (100,000 to 390,000) | | 230,000 (10,000 to 340,000) | | 120,000 (300 to 240,000) |
| Symptomatic attack rate | 30.7% (19.6 to 34.6) | 24.1% (3.2 to 30.8) | 15.3% (0.1 to 24.8) | 27.2% (13.3 to 32.3) | | 23.7% (2.7 to 30.2) | | 14.9% (0 to 24.4) |
| Deaths per 1000 person-years | 1.9 (0.9 to 2.6) | 1.3 (0.1 to 2) | 0.7 (0 to 1.4) | 1.4 (0.5 to 1.9) | | 1.1 (0.1 to 1.7) | | 0.6 (0 to 1.2) |
| Epidemic peak (months) | 4 (3 to 7) | 5 (3 to 12) | 5 (4 to 6) | 4 (3 to 10) | | 5 (3 to 12) | | 5 (4 to 6) |
| Peak deaths | 10,000 (3,000 to 20,000) | 5,000 (300 to 10,000) | 2,000 (8 to 6,000) | 7,000 (1,000 to 10,000) | | 5,000 (200 to 10,000) | | 1,000 (8 to 5,000) |
| Peak non-ICU beds needed | 330,000 (80,000 to 580,000) | 160,000 (10,000 to 350,000) | 50,000 (100 to 170,000) | 210,000 (40,000 to 390,000) | | 140,000 (7,000 to 300,000) | | 40,000 (100 to 150,000) |
| Peak ICU beds needed | 170,000 (40,000 to 300,000) | 80,000 (5,000 to 180,000) | 30,000 (70 to 90,000) | 110,000 (20,000 to 210,000) | | 70,000 (4,000 to 160,000) | | 20,000 (60 to 80,000) |

| Mauritius | | | | | | | | |
| --- | --- | --- | --- | --- | --- | --- | --- | --- |
| *Using a distribution of* $\boldsymbol{R}_{\boldsymbol{t}}$ *estimates in African countries* | | | | | | | | |
| Key outcome | **Unmitigated** | **20% distancing** | **50% distancing** | **80% shielding** | | **80% shielding + 20% distancing** | | **80% shielding + 50% distancing** |
| Symptomatic cases | 310,000 (2,000 to 540,000) | 140,000 (200 to 470,000) | 4,000 (200 to 370,000) | | 210,000 (100 to 460,000) | 120,000 (100 to 420,000) | 3,000 (100 to 340,000) | |
| Severe cases | 20,000 (100 to 50,000) | 9,000 (10 to 40,000) | 300 (10 to 30,000) | | 10,000 (9 to 30,000) | 6,000 (9 to 20,000) | 200 (9 to 20,000) | |
| Critical cases | 9,000 (40 to 20,000) | 4,000 (4 to 20,000) | 100 (4 to 10,000) | | 5,000 (5 to 10,000) | 2,000 (5 to 10,000) | 80 (5 to 8,000) | |
| Deaths | 6,000 (20 to 10,000) | 2,000 (2 to 10,000) | 70 (2 to 7,000) | | 3,000 (2 to 7,000) | 1,000 (2 to 7,000) | 50 (2 to 5,000) | |
| Symptomatic attack rate | 24.1% (0.1 to 41.7) | 10.8% (0 to 36.1) | 0.3% (0 to 28.6) | | 16.2% (0 to 35.1) | 9.1% (0 to 32.5) | 0.3% (0 to 26) | |
| Deaths per 1000 person-years | 4.5 (0 to 9.6) | 1.8 (0 to 7.7) | 0.1 (0 to 5.6) | | 2.2 (0 to 5.6) | 1.1 (0 to 5.1) | 0 (0 to 3.8) | |
| Epidemic peak (months) | 5 (2 to 12) | 5 (0 to 11) | 3 (0 to 9) | | 5 (0 to 12) | 5 (0 to 12) | 3 (0 to 9) | |
| Peak deaths | 100 (2 to 500) | 30 (1 to 300) | 3 (1 to 200) | | 40 (1 to 300) | 20 (1 to 200) | 3 (1 to 100) | |
| Peak non-ICU beds needed | 3,000 (8 to 10,000) | 500 (3 to 9,000) | 30 (3 to 5,000) | | 1,000 (2 to 8,000) | 300 (2 to 6,000) | 20 (2 to 3,000) | |
| Peak ICU beds needed | 2,000 (4 to 8,000) | 300 (2 to 5,000) | 20 (2 to 3,000) | | 500 (1 to 4,000) | 200 (1 to 3,000) | 10 (1 to 2,000) | |
| *Using global* $\boldsymbol{R}_{\boldsymbol{0}}$ *estimates* | | | | | | | | |
| Key outcome | **Unmitigated** | **20% distancing** | **50% distancing** | **80% shielding** | | **80% shielding + 20% distancing** | | **80% shielding + 50% distancing** |
| Symptomatic cases | 470,000 (290,000 to 560,000) | 370,000 (110,000 to 480,000) | 230,000 (2,000 to 380,000) | 380,000 (190,000 to 470,000) | | 330,000 (80,000 to 430,000) | | 210,000 (2,000 to 340,000) |
| Severe cases | 40,000 (20,000 to 50,000) | 30,000 (6,000 to 40,000) | 20,000 (100 to 30,000) | 20,000 (9,000 to 30,000) | | 20,000 (4,000 to 30,000) | | 10,000 (90 to 20,000) |
| Critical cases | 20,000 (8,000 to 20,000) | 10,000 (3,000 to 20,000) | 7,000 (60 to 10,000) | 9,000 (4,000 to 10,000) | | 8,000 (2,000 to 10,000) | | 5,000 (40 to 8,000) |
| Deaths | 10,000 (5,000 to 10,000) | 7,000 (2,000 to 10,000) | 4,000 (30 to 8,000) | 6,000 (2,000 to 8,000) | | 5,000 (1,000 to 7,000) | | 3,000 (20 to 5,000) |
| Symptomatic attack rate | 36.4% (22.3 to 42.8) | 28.3% (8.2 to 37.2) | 17.9% (0.2 to 29.6) | 29.5% (14.4 to 35.8) | | 25.5% (6.4 to 33.2) | | 15.9% (0.1 to 26.5) |
| Deaths per 1000 person-years | 7.7 (3.9 to 10.5) | 5.6 (1.3 to 8.4) | 3.3 (0 to 6.1) | 4.4 (1.9 to 6) | | 3.7 (0.7 to 5.3) | | 2.2 (0 to 4) |
| Epidemic peak (months) | 3 (2 to 5) | 3 (2 to 9) | 3 (2 to 5) | 3 (2 to 7) | | 3 (2 to 10) | | 3 (2 to 5) |
| Peak deaths | 300 (90 to 600) | 200 (20 to 400) | 60 (2 to 200) | 200 (30 to 300) | | 100 (10 to 200) | | 40 (2 to 100) |
| Peak non-ICU beds needed | 9,000 (2,000 to 20,000) | 5,000 (400 to 10,000) | 2,000 (10 to 6,000) | 5,000 (800 to 8,000) | | 3,000 (200 to 6,000) | | 1,000 (10 to 3,000) |
| Peak ICU beds needed | 5,000 (1,000 to 8,000) | 3,000 (200 to 5,000) | 900 (8 to 3,000) | 2,000 (400 to 4,000) | | 2,000 (100 to 3,000) | | 500 (6 to 2,000) |

#### **Table S6: Relative reductions in key outcomes during the first 12 months of the epidemic under different strategies, compared to an unmitigated epidemic, using empirical contact matrices.**

| Key outcome | Country | 20% distancing | 50% distancing | 80% shielding | 80% shielding + 20% distancing | 80% shielding + 50% distancing |
| --- | --- | --- | --- | --- | --- | --- |
| *Using country specific* $\boldsymbol{R}_{\boldsymbol{t}}$ *estimates* | | | | | | |
| Total deaths in first 12 months | Niger | 63.2% (39.7 to 99.4) | 99% (71.7 to 99.8) | 40.3% (30.1 to 99.4) | 67.9% (46.3 to 99.7) | 99.1% (73.4 to 99.8) |
|  | Nigeria | 89.7% (41 to 99.8) | 99.6% (79.3 to 99.9) | 49.3% (30.2 to 99.6) | 90.9% (48.1 to 99.8) | 99.6% (81.8 to 99.9) |
|  | Mauritius | 58.6% (18.6 to 99.4) | 98.1% (37.9 to 99.6) | 52.7% (40.9 to 99.2) | 75% (46.9 to 99.6) | 98.8% (59.3 to 99.8) |
| Hospital bed demand at epidemic peak | Niger | 63.5% (39.9 to 99.5) | 99% (73.3 to 99.8) | 40.1% (30 to 99.4) | 66.9% (46.2 to 99.7) | 99.1% (75.2 to 99.8) |
|  | Nigeria | 87.9% (41.7 to 99.8) | 99.6% (78.9 to 99.9) | 47.7% (30.6 to 99.6) | 90.7% (48.2 to 99.8) | 99.6% (81.4 to 99.9) |
|  | Mauritius | 58.1% (17.5 to 99.3) | 98.2% (36.2 to 99.6) | 52.9% (40.5 to 99.3) | 73.8% (46.2 to 99.5) | 98.7% (59 to 99.8) |
| Cases | Niger | 51.3% (24.5 to 99.4) | 98.6% (57.4 to 99.7) | 24.4% (12.7 to 99.4) | 52.5% (25.6 to 99.7) | 98.6% (57.9 to 99.8) |
|  | Nigeria | 81.8% (28.8 to 99.8) | 99.5% (68.6 to 99.9) | 32.2% (15.1 to 99.7) | 85.2% (30.5 to 99.8) | 99.5% (69.5 to 99.9) |
|  | Mauritius | 54.1% (13.1 to 99.4) | 98% (27.4 to 99.6) | 34% (15.2 to 99.3) | 62.7% (21 to 99.5) | 98.3% (36.6 to 99.7) |
| *Using global* $\boldsymbol{R}_{\boldsymbol{0}}$ *estimates* | | | | | | |
| Total deaths in first 12 months | Niger | 36.3% (23.6 to 78.4) | 67.2% (49 to 99.6) | 29.7% (23.6 to 44.7) | 43.9% (33 to 81.2) | 70.2% (54 to 99.7) |
|  | Nigeria | 34.3% (22.9 to 91.6) | 65.7% (47.2 to 99.8) | 28.4% (22.6 to 53.1) | 42.4% (32.1 to 93.5) | 69.5% (52.7 to 99.8) |
|  | Mauritius | 27.9% (18.2 to 70.5) | 57.6% (38.6 to 99.5) | 43.4% (38.9 to 57) | 52.3% (45.1 to 83.4) | 72.1% (58.4 to 99.6) |
| Hospital bed demand at epidemic peak | Niger | 36.2% (23.8 to 76.6) | 67% (48.7 to 99.6) | 29.6% (23.5 to 44.6) | 43.6% (32.8 to 79.6) | 70.2% (53.9 to 99.7) |
|  | Nigeria | 34.4% (22.8 to 90.1) | 65.7% (47.1 to 99.8) | 28.3% (22.6 to 50.3) | 42.5% (32.1 to 92.4) | 69.5% (52.7 to 99.8) |
|  | Mauritius | 28.2% (18.5 to 68.6) | 57.7% (38.7 to 99.4) | 43.3% (39.1 to 56.5) | 52.4% (45.6 to 82.3) | 71.9% (58.7 to 99.6) |
| Cases | Niger | 20.4% (10.1 to 66.4) | 48% (26.3 to 99.4) | 10.9% (5.9 to 28.6) | 21.6% (11.4 to 68.3) | 48.9% (27.2 to 99.5) |
|  | Nigeria | 21.5% (11.1 to 84.3) | 50.1% (28 to 99.7) | 11.6% (6.4 to 34.4) | 23% (12.5 to 86.7) | 51.3% (29.2 to 99.8) |
|  | Mauritius | 22.2% (12.4 to 64.5) | 50.9% (29.7 to 99.3) | 19% (14 to 38.2) | 29.9% (20.1 to 72.9) | 56.4% (36.1 to 99.5) |

## Sensitivity to assumed infectiousness of asymptomatic cases

In our main analysis, we assumed asymptomatic cases to be half as infectious as symptomatic cases ($f=50\%$). We re-ran the analysis assuming asymptomatic cases are as infectious as pre- symptomatic and symptomatic cases ($f=100\%$) or not infectious at all ($f=0\%$). Note that we calibrated $R_{0}$­ to take the same value as in the main analysis.

As expected, when $f=100\%$, the effect of self-isolation is much smaller, as individuals with symptoms attribute to the overall force of infection to a smaller extent (Figure S7). In contrast, when $f=0\%$, transmission only results from pre-symptomatic and symptomatic individuals: if the latter self-isolate, a much greater reduction in transmission would be expected. By contrast, the effect of general distancing is insensitive to assumptions on asymptomatic infectiousness (Figure S7), since reducing everyone’s contacts will have a similar effect overall, regardless of which group is most responsible for transmission.

As regards shielding, the main sensitivity is to quadrupling of contacts among shielded people (Figure S8), which would greatly decrease the effect of shielding if asymptomatic people are not infectious at all: given that $R_{0}$ remains the same, in this sensitivity scenario the force of infection is attributable entirely to pre-symptomatic and symptomatic individuals, who in turn are relatively much more frequent among the elderly. In other words, the elderly contribute much more to the force of infection, which, combined with a quadrupling of contacts among them if shielded, dampens the effect of this intervention.

As shown in Figure S9, strategies generally have a greater effect if asymptomatic cases are not infectious at all, than if they are fully infectious. Recall that all strategies consider include 50% self-isolation of symptomatic cases: as shown above, the latter intervention would make a much greater contribution to overall transmission reduction if there are no asymptomatic transmitters.

#### Assuming asymptomatic cases are as infectious as symptomatic cases ($f=100\%$)

####
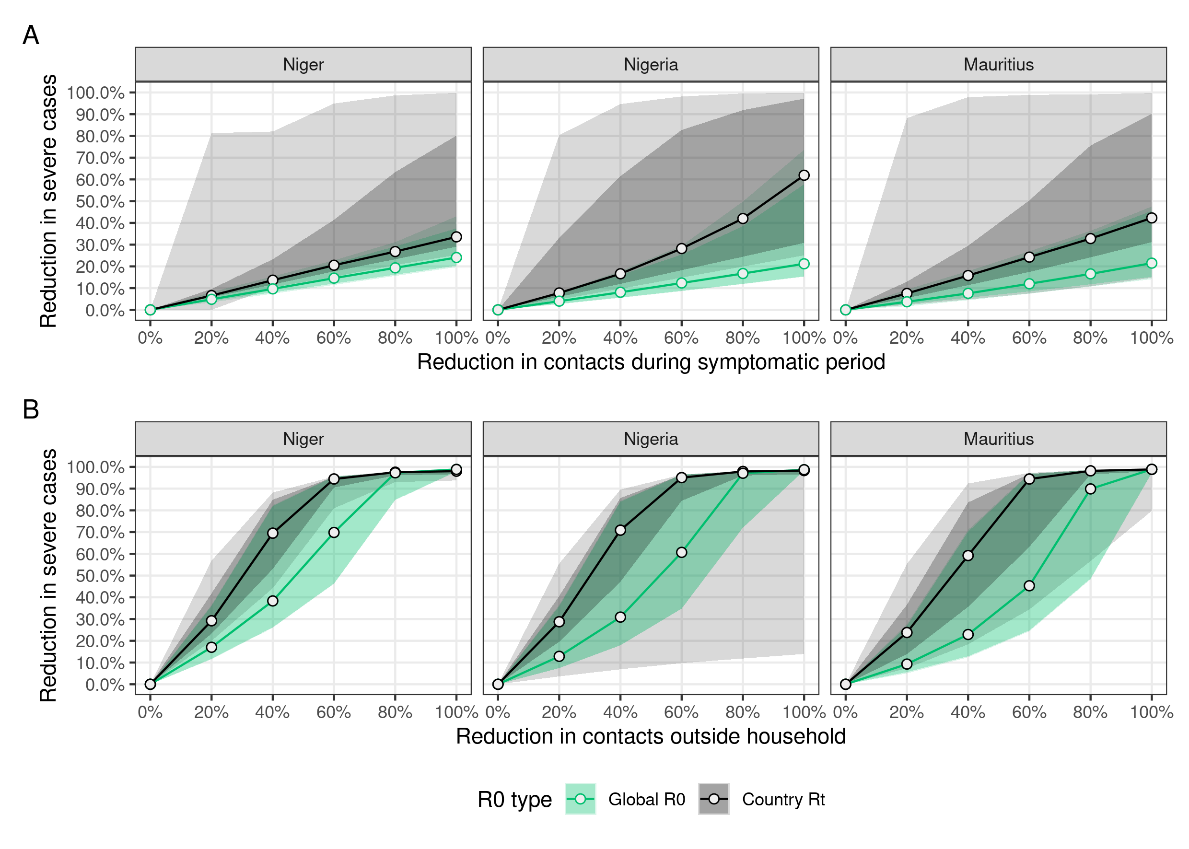


#### Assuming asymptomatic cases are not infectious at all ($f=0\%$)

#### **
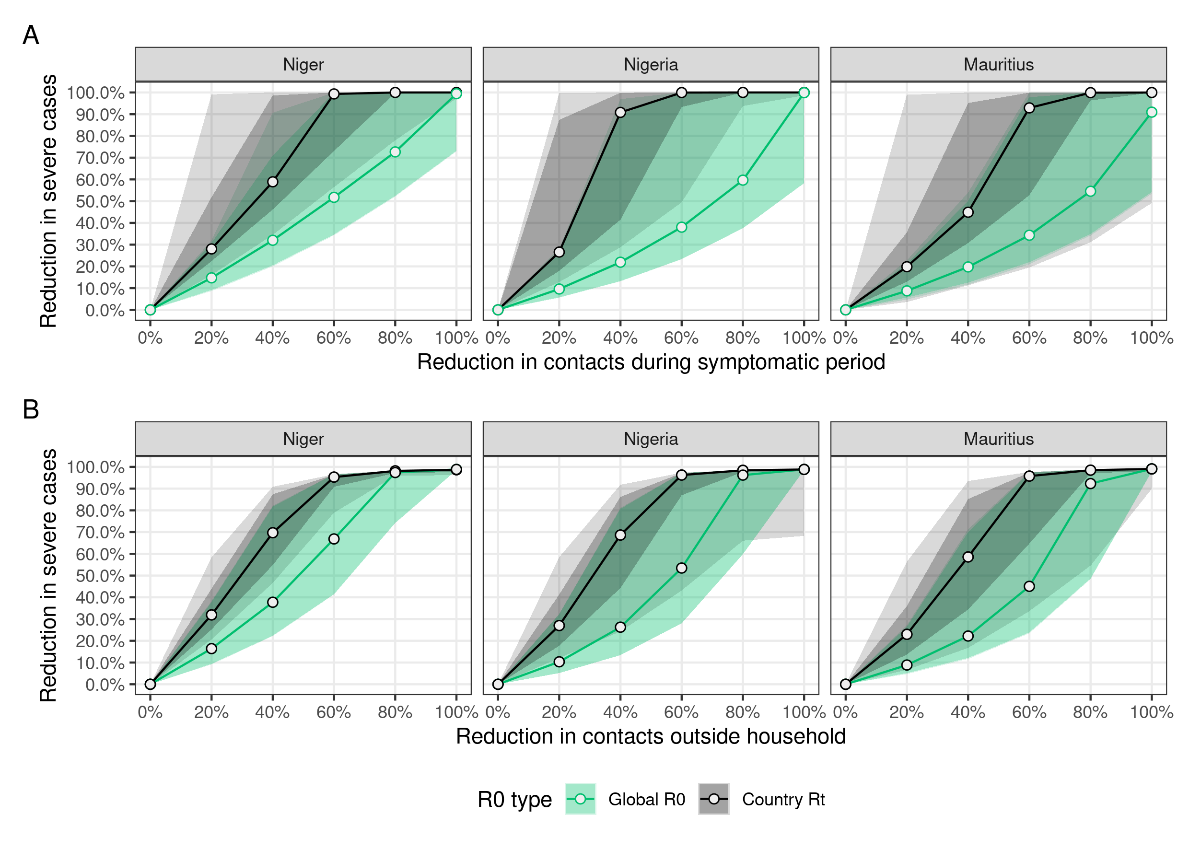
**

#### **Figure S7. Relative impact of A) self-isolation and B) general distancing under different assumptions on the relative infectiousness of asymptomatic cases.**

#### Assuming asymptomatic cases are as infectious as symptomatic cases ($f=100\%$)

####
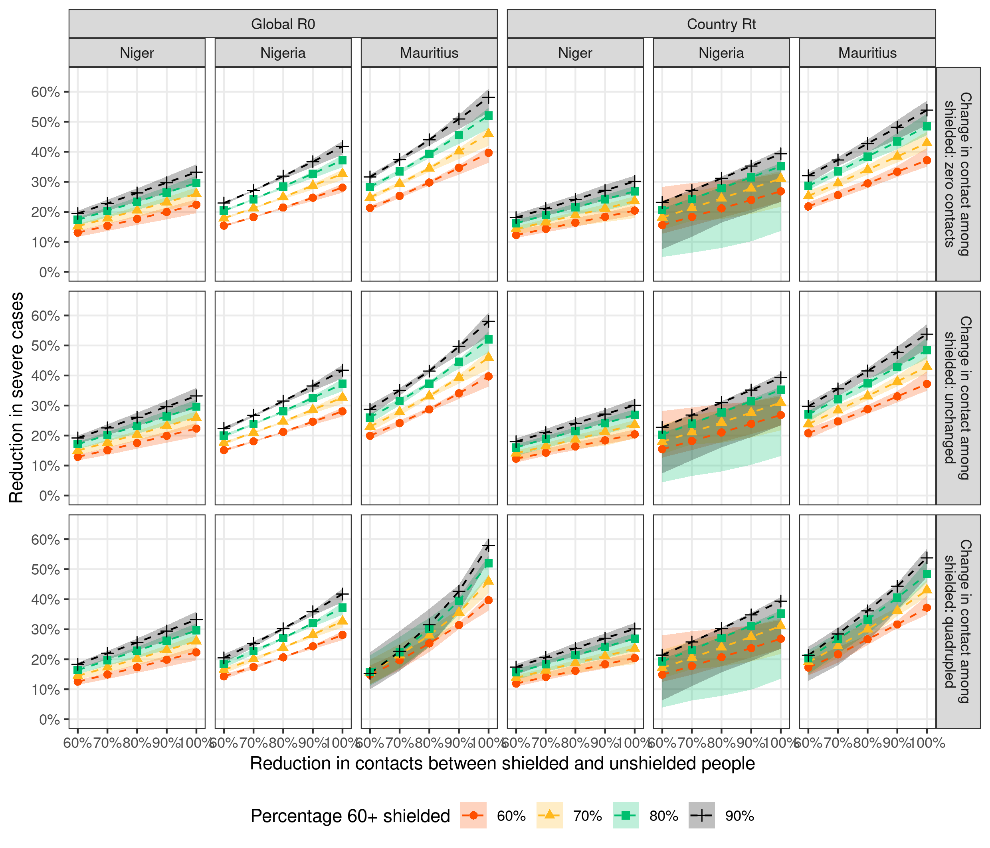


#### Assuming asymptomatic cases are not infectious at all ($f=0\%$)

#### **
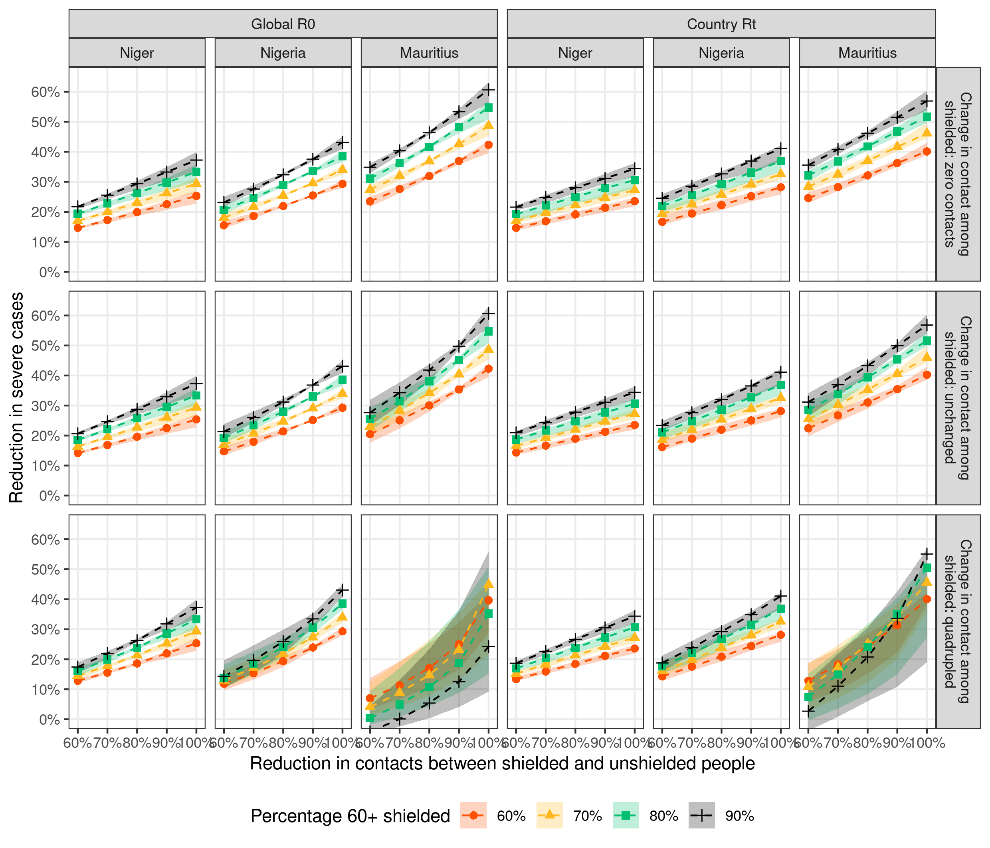
**

#### **Figure S8. Relative impact of shielding under different assumptions on the relative infectiousness of asymptomatic cases.**

| Assuming asymptomatic cases are as infectious as symptomatic cases($f=100\%$) | 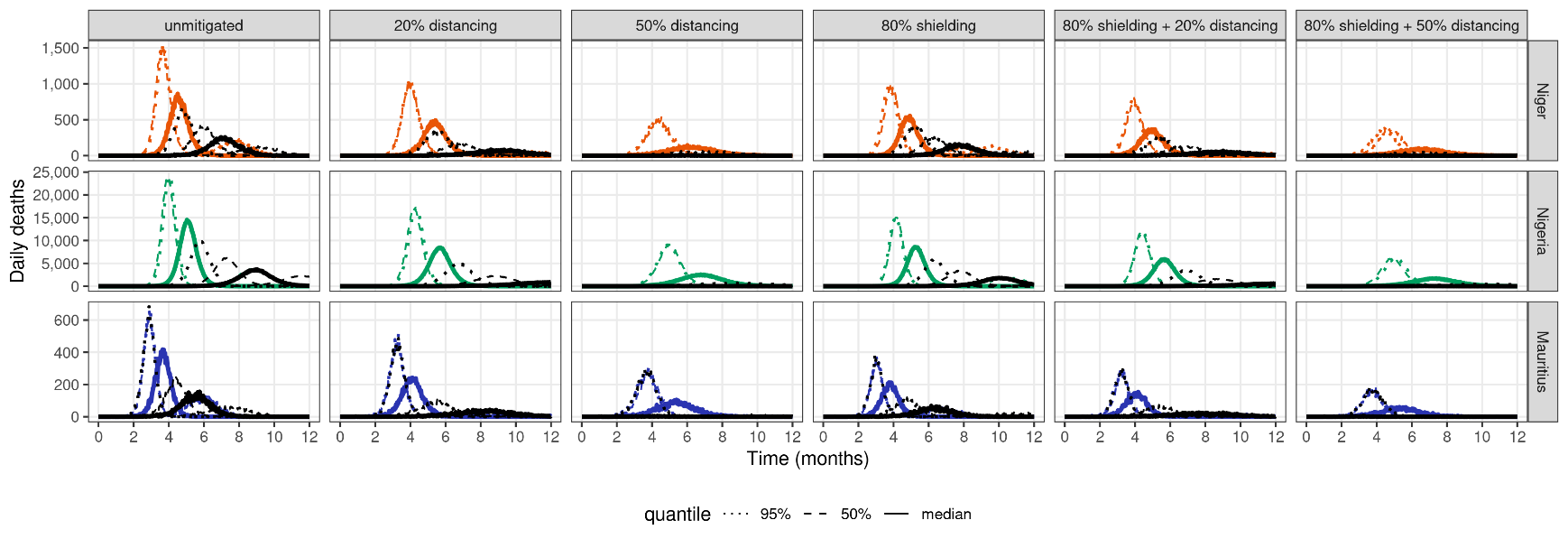 |
| --- | --- |
| Assuming asymptomatic cases are not infectious at all($f=0\%$) | 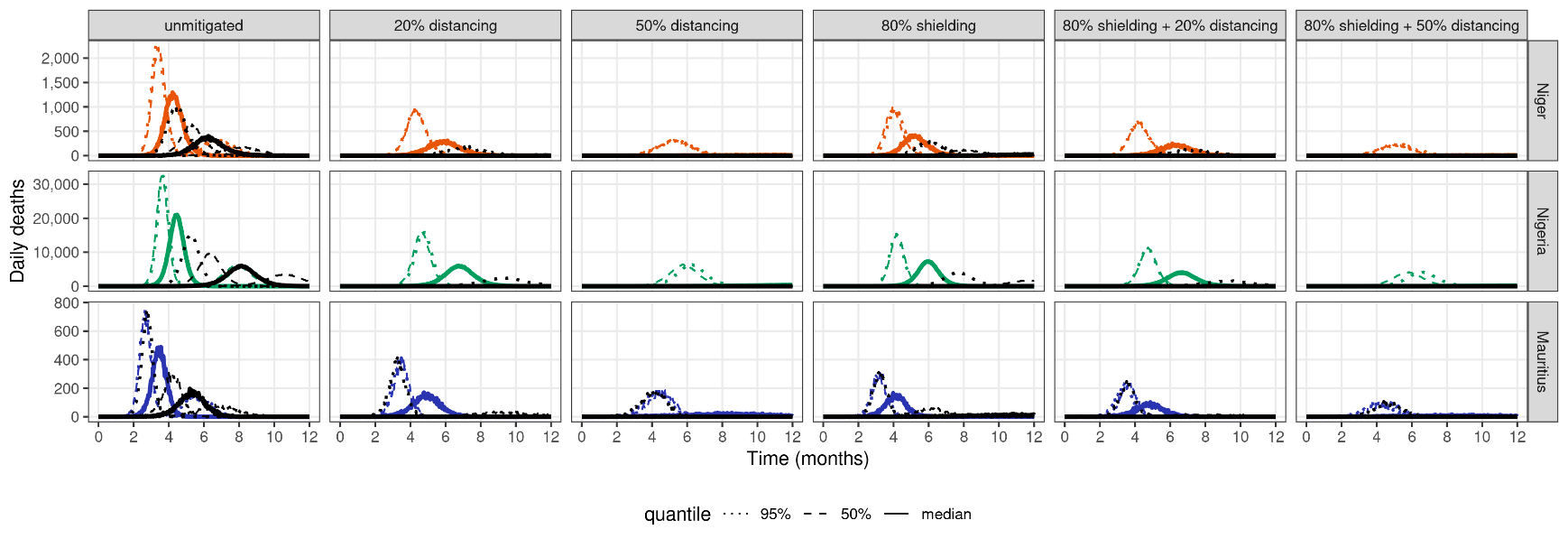 |

#### **Figure S9. Deaths over time under different strategies under alternative assumptions on the relative infectiousness of asymptomatic cases.** Black lines show estimates for scenarios using country specific $R_{t}$ estimates, while coloured lines show estimates for scenarios using global $R_{\boldsymbol{0}}$ estimates.

## Sensitivity to transmissibility assumption ($R_{0}$)

Uncertainty intervals in our results, and differences between country specific $R_{t}$ estimates and global $R_{\boldsymbol{0}}$ estimates, were mainly reflective of the range of $R_{0}$ values we sampled in the model. Here we show our results stratified by $R_{0}$ values, as sampled across 400 runs from a normal $R_{0}$ distribution with mean 2.6 and standard deviation of 0.5.

Figure S10 shows the unmitigated epidemic in each country for every model run (i.e. randomly sampled $R_{0}$value) with $R_{0}$ grouped into values <2, between 2 and 3, and >3. Epidemics with high $R_{0}$ have a higher peak number of cases and total number of cases, and will peak earlier, whereas epidemics with a low$R_{0}$will have a lower peak number of cases and total number of cases, and will peak later.


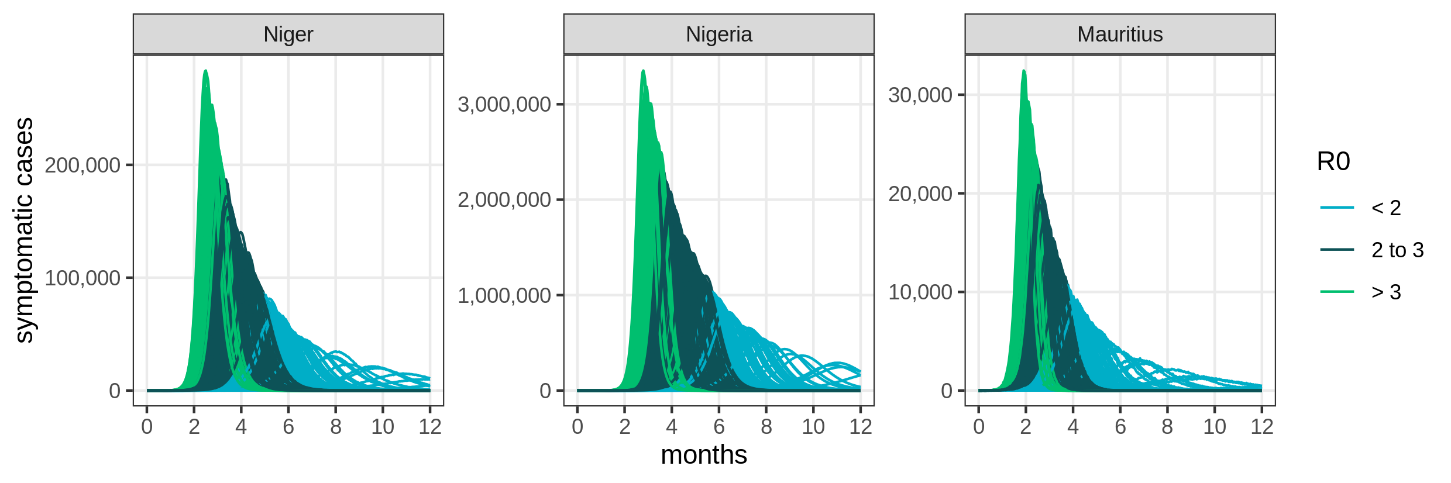


#### **Figure S10. Projected incidence of symptomatic COVID-19 cases over time for simulations of an unmitigated epidemic, by** $\boldsymbol{R}_{\mathbf{0}}$ **value.**

Figure S11 shows the impact of self-isolation and general distancing (equivalent to Figure 2 in the main text), but for every single run and stratified by $R_{0}$. High reductions as a result of self-isolation may only be achieved with very low values of $R_{0}$, and are less likely in Niger, which has a younger population (i.e. symptomatic individuals are relatively fewer and thus contribute less to transmissibility). However, with higher values of $R_{0}$, the impact of self-isolation is roughly similar across countries. shows the impact of physical distancing on the total number of severe cases. The impact of physical distancing can be substantially lower when $R_{0}$ is higher, as greater reductions in contacts will be needed to bring $R$ closer to 1. Generally, runs with smaller $R_{0}$ are more affected by stochasticity in the model, which is why these estimates do not always continue their upward trend.


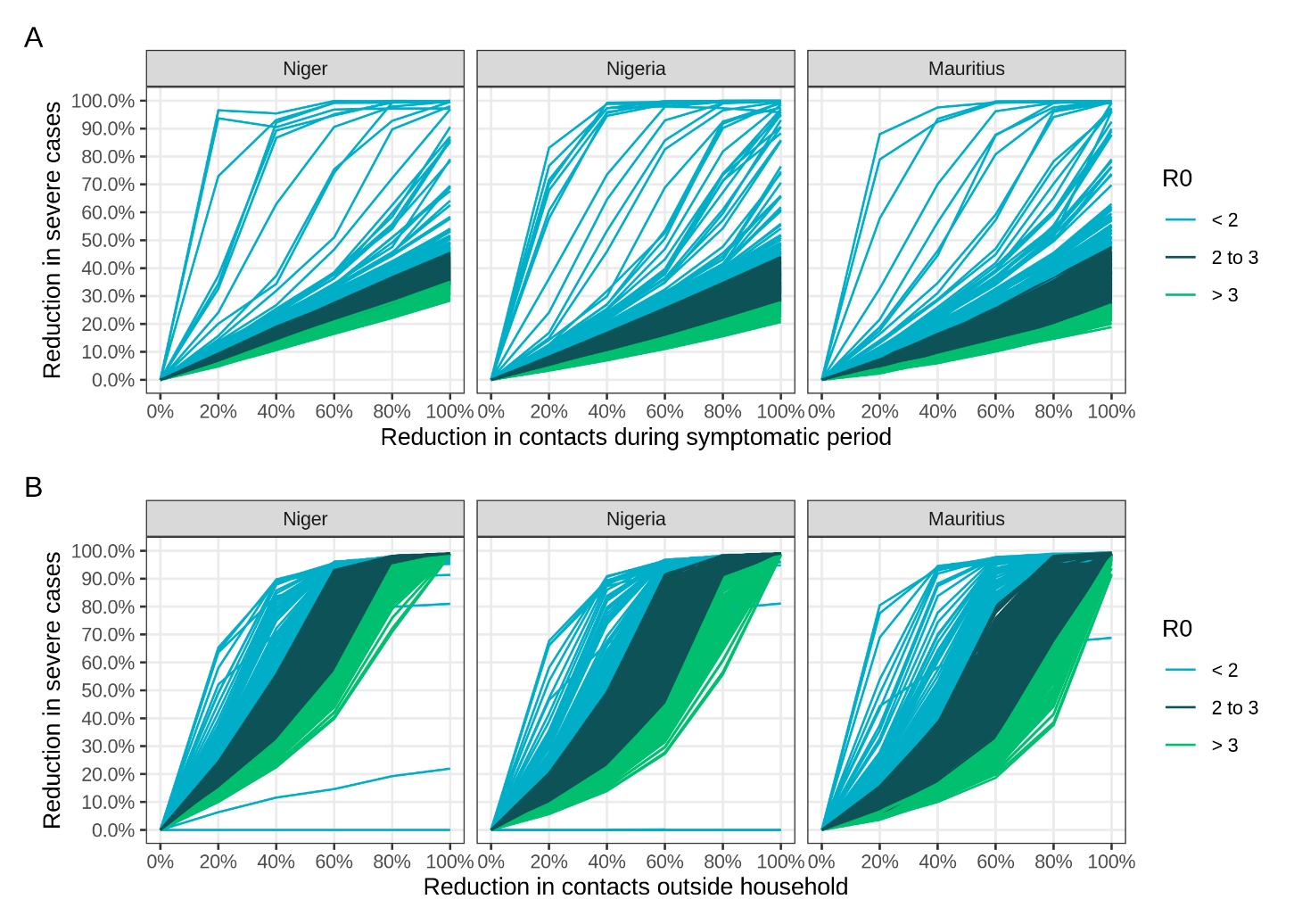


#### **Figure S11. Impact of A) self-isolation and B) general distancing, by** $\boldsymbol{R}_{\mathbf{0}}$ **value sampled.**

Figure S12 shows the impact of shielding 80% of people aged ≥ 60 years old under various reductions in contacts between the shielded and unshielded population, and changes in contact within the shielded population, for every single run. As shielding does not significantly affect the overall transmission dynamics in the population, impact does not vary much between different values of $R_{0}$. In Mauritius, where 18% of the population are ≥ 60 years old (i.e. 15% are shielded under a shielding coverage of 80%), variation in $R_{0}$ matters if contact within the shielded population changes from baseline, as this does significantly affect transmission dynamics in the overall population (the 15% of people shielded are relatively more infectious than the average).


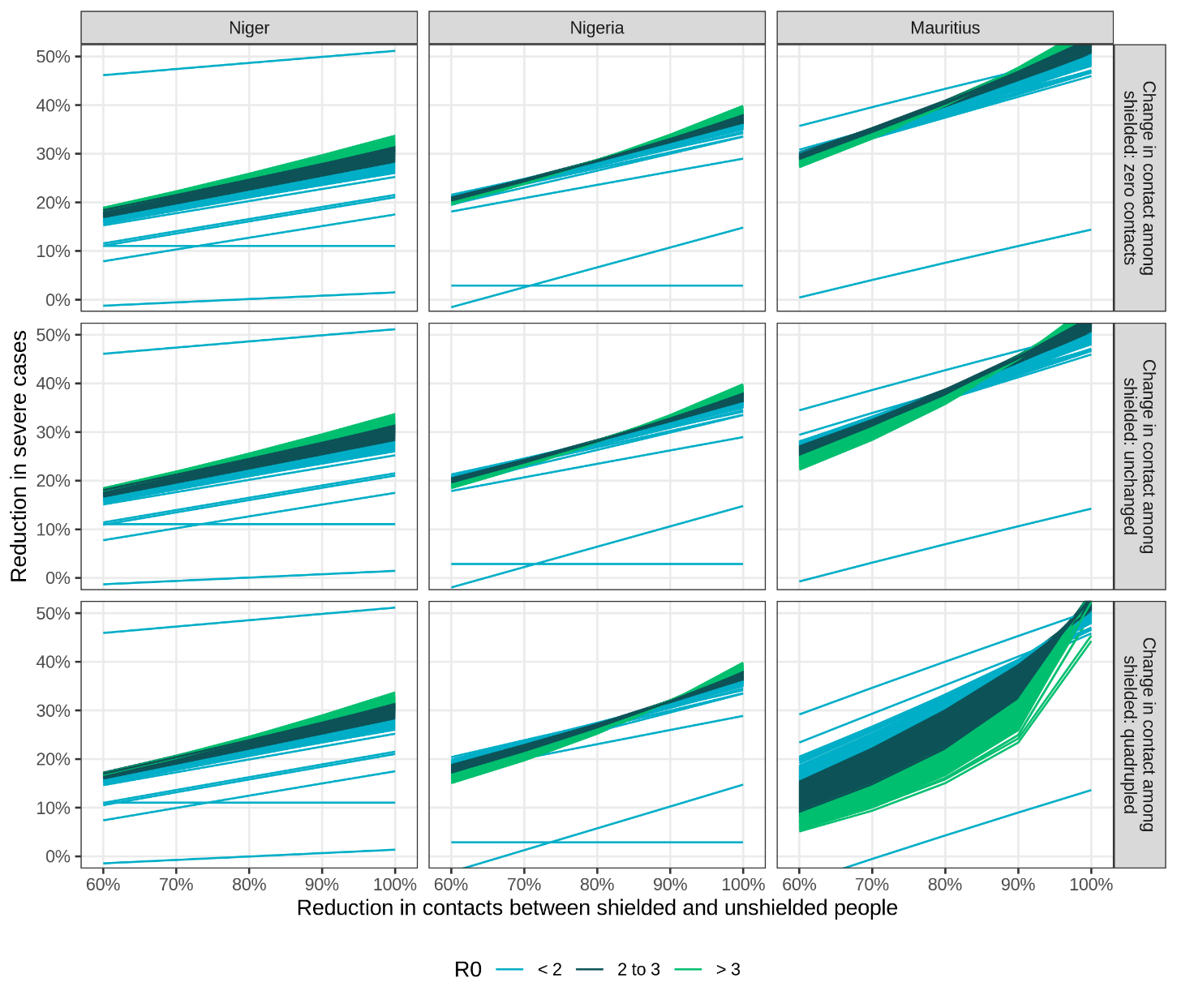


#### **Figure S12. Impact of shielding 80% of the population aged ≥ 60 years, by** $\boldsymbol{R}_{\boldsymbol{0}}$ **value sampled.**

## Sensitivity to disease severity assumptions

The assumption on the shift in severity risk to younger groups has a negligible effect on the impact of self-isolation and social distancing, since severity is not assumed to affect infectiousness (Figure S13).

Shielding, however, is sensitive to the age shift assumption, as this intervention specifically focusses on the elderly. If no age shift is applied, the relative reduction in severe cases across all age groups would be greater, as the overall risk in younger ages would be smaller (Figure S14).

The impact on absolute numbers is significant. Table S6 shows the estimated number of severe cases, critical cases, deaths, and hospital bed demand under different assumptions on the age shift in severity risk and the CFR. Shifting the severity estimates towards younger group by 10 years can more than double the estimates, with the impact being greatest in Niger, where this shift would affect the largest proportion of the population of the three countries studied.

As expected, the impact of increasing the assumed CFR scales the death estimates linearly, and illustrate the extent to which the COVID-19 death toll could vary as a function of availability of treatment and other factors affecting the infection’s lethality.

#### 5y shift

####
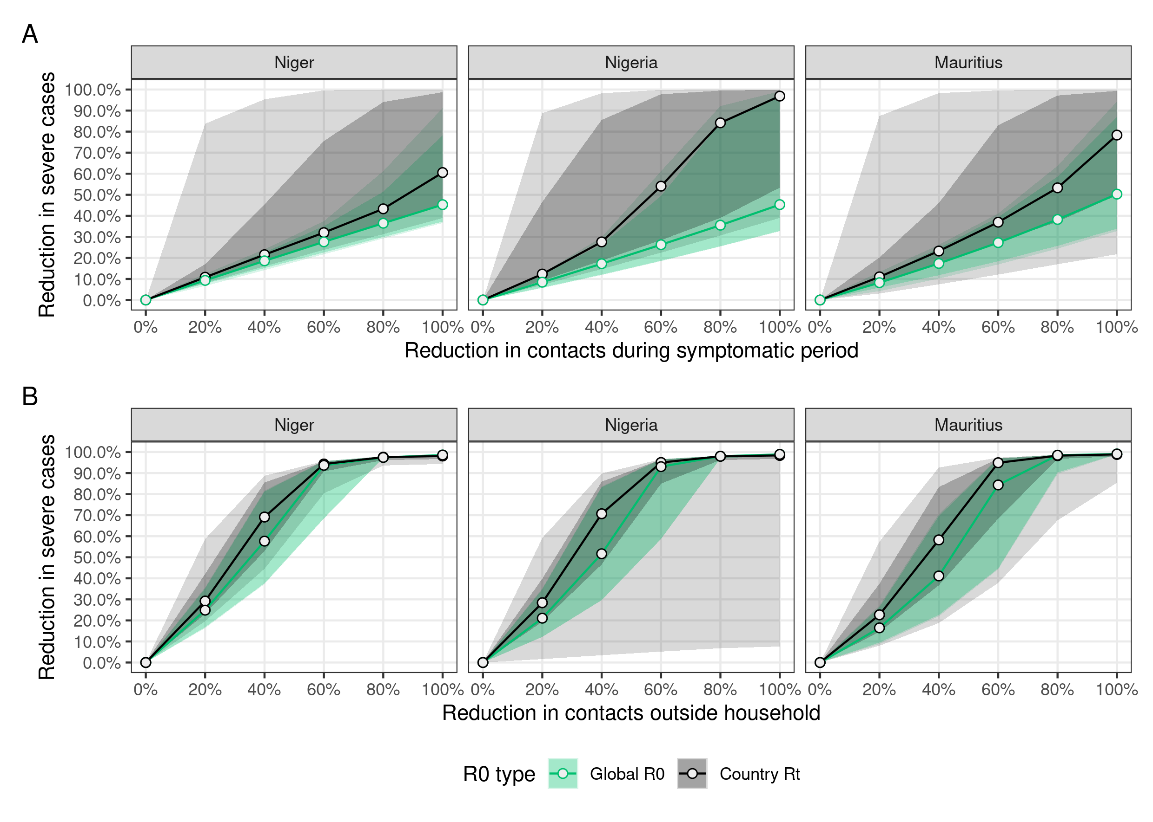


#### 0y shift

#### **
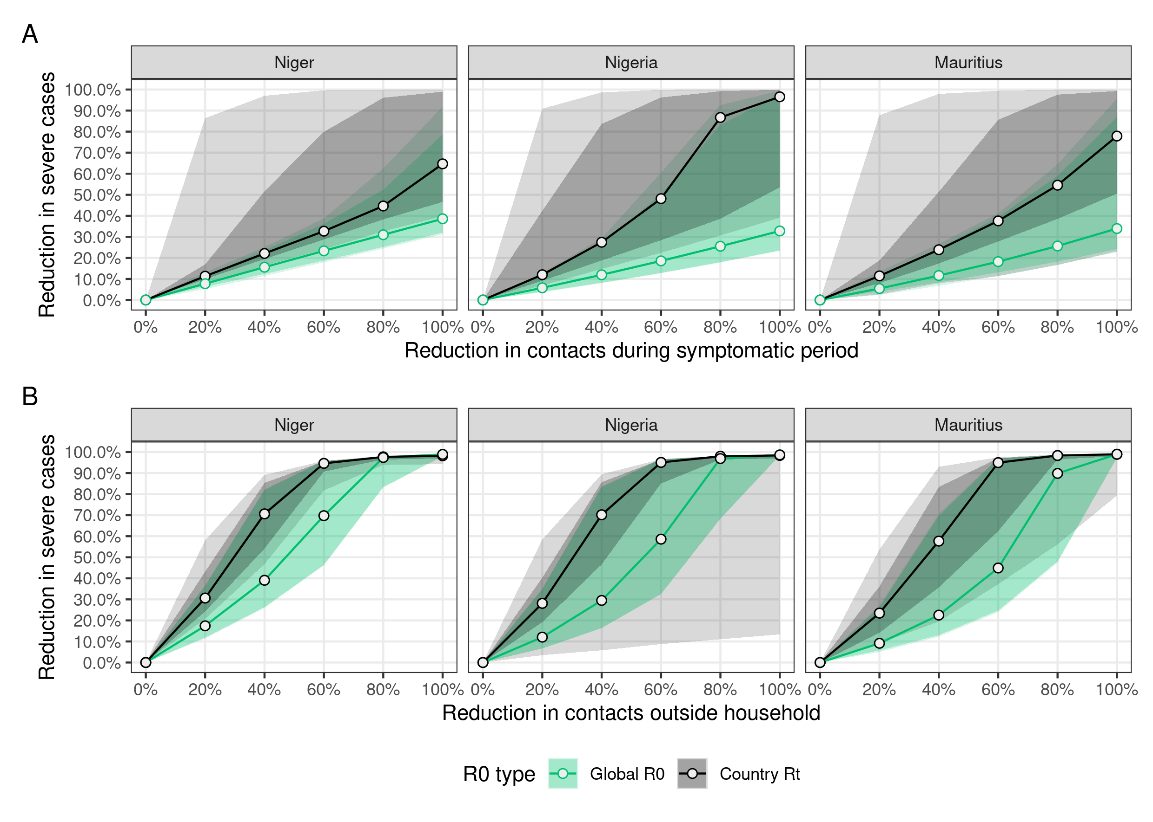
**

#### **Figure S13. Sensitivity analysis: relative impact of A) self-isolation and B) general distancing under different assumptions on the age shift in the risk of severe disease among African populations.**

#### 5y shift

####
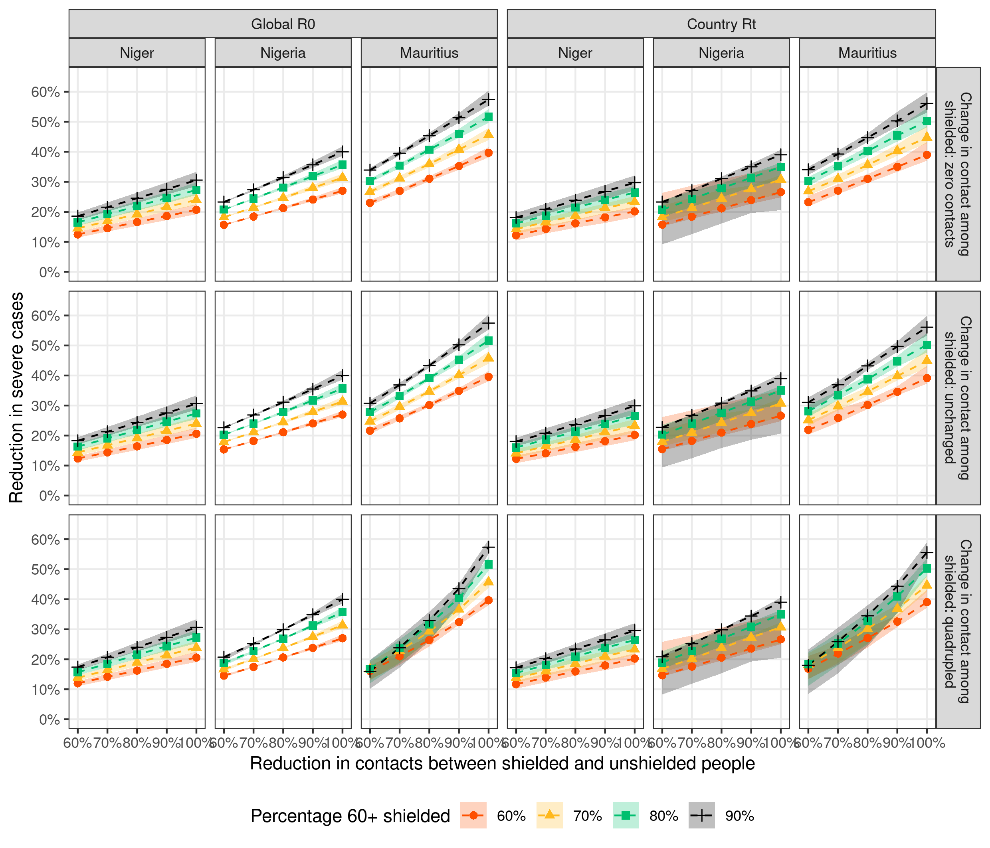


#### 0y shift

#### **
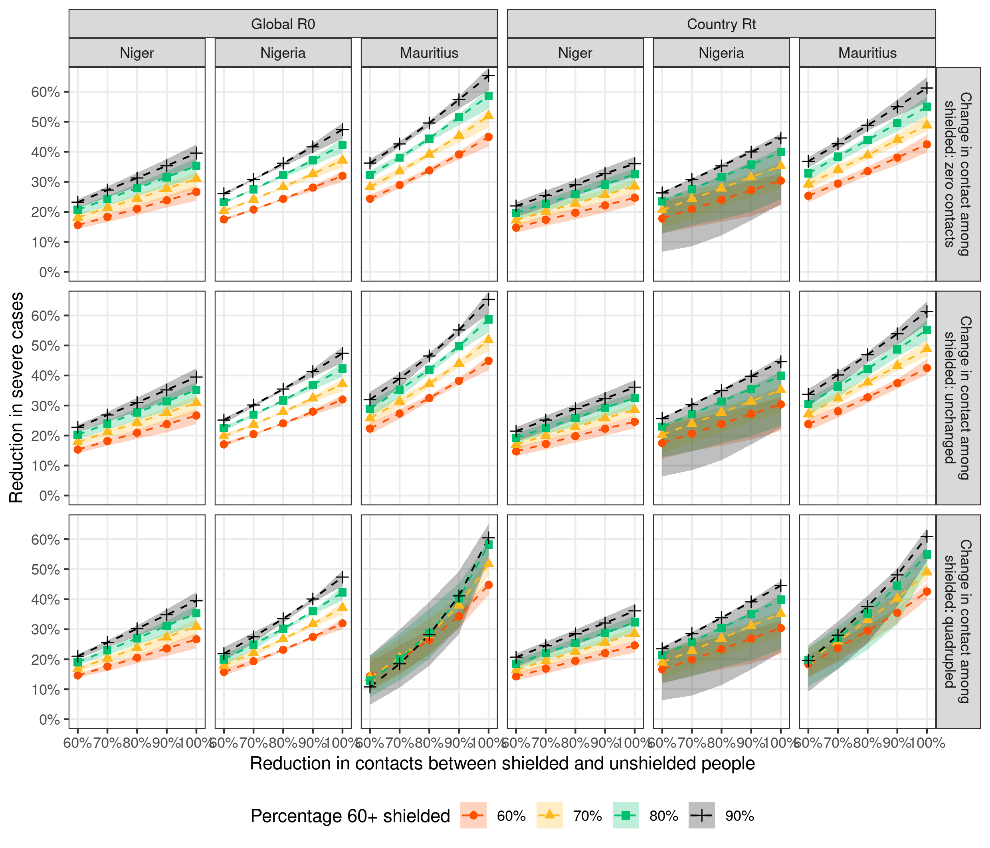
**

#### **Figure S14. Sensitivity analysis: relative impact of shielding under different assumptions on the age shift in the risk of severe disease among African populations.**

#### **Table S6. Outcomes of an unmitigated epidemic under different assumptions on the age shift in the risk of severe disease and on the case-fatality ratio of symptomatic cases among African populations.**

|  | | | Key outcome | 10y age-shift | 5y age-shift | 0y age-shift |
| --- | --- | --- | --- | --- | --- | --- |
| Niger | | | | | | |
| *Using Niger specific* $\boldsymbol{R}_{\boldsymbol{t}}$ *estimates* | | | | | | |
|  | | | Severe cases | 60,000 (300 to 110,000) | 40,000 (100 to 80,000) | 30,000 (100 to 50,000) |
|  |  |  | Critical cases | 30,000 (100 to 50,000) | 20,000 (60 to 30,000) | 10,000 (70 to 20,000) |
| CFR increase | | x 1.0 | Deaths | 10,000 (50 to 20,000) | 5,000 (10 to 10,000) | 5,000 (20 to 8,000) |
|  |  | x 1.5 | Deaths | 20,000 (70 to 30,000) | 10,000 (30 to 20,000) | 7,000 (30 to 10,000) |
|  |  | x 2.0 | Deaths | 30,000 (150 to 50,000) | 15,000 (50 to 30,000) | 11,000 (50 to 15,000) |
|  |  | x 1.0 | Peak deaths | 100 (2 to 400) | 100 (2 to 300) | 50 (1 to 200) |
|  |  | x 1.5 | Peak deaths | 300 (4 to 700) | 200 (2 to 500) | 100 (3 to 300) |
|  |  | x 2.0 | Peak deaths | 400 (5 to 900) | 300 (10 to 700) | 200 (10 to 500) |
|  | | | Peak non-ICU beds needed | 8,000 (40 to 20,000) | 6,000 (20 to 10,000) | 3,000 (20 to 9,000) |
|  |  |  | Peak ICU beds needed | 4,000 (20 to 10,000) | 3,000 (10 to 8,000) | 2,000 (10 to 5,000) |
| *Using global* $\boldsymbol{R}_{\boldsymbol{0}}$ *estimates* | | | | | | |
|  | | | Severe cases | 130,000 (50,000 to 190,000) | 60,000 (40,000 to 90,000) | 60,000 (20,000 to 80,000) |
|  |  |  | Critical cases | 60,000 (20,000 to 80,000) | 20,000 (20,000 to 40,000) | 20,000 (10,000 to 40,000) |
| CFR increase | x 1.0 | | Deaths | 10,000 (5,000 to 20,000) | 15,000 (8,000 to 16,000) | 13,000 (4,000 to 13,000) |
|  | x 1.5 | | Deaths | 30,000 (10,000 to 50,000) | 20,000 (10,000 to 20,000) | 20,000 (6,000 to 20,000) |
|  | x 2.0 | | Deaths | 50,000 (15,000 to 75,000) | 30,000 (15,000 to 30,000) | 30,000 (9,000 to 30,000) |
|  | x 1.0 | | Peak deaths | 600 (100 to 1,000) | 200 (100 to 400) | 200 (10 to 300) |
|  | x 1.5 | | Peak deaths | 900 (200 to 2,000) | 300 (200 to 700) | 400 (100 to 800) |
|  | x 2.0 | | Peak deaths | 1,500 (300 to 3,000) | 500 (300 to 1,000) | 500 (200 to 1,000) |
|  | | | Peak non-ICU beds needed | 30,000 (6,000 to 50,000) | 9,000 (4,000 to 20,000) | 10,000 (3,000 to 20,000) |
|  |  |  | Peak ICU beds needed | 10,000 (3,000 to 30,000) | 5,000 (2,000 to 10,000) | 6,000 (1,000 to 10,000) |
| Nigeria | | | | | | |
| *Using Nigeria specific* $\boldsymbol{R}_{\boldsymbol{t}}$ *estimates* | | | | | | |
|  | | | Severe cases | 1,020,000 (100 to 1,740,000) | 670,000 (60 to 1,140,000) | 460,000 (40 to 790,000) |
|  |  |  | Critical cases | 440,000 (40 to 750,000) | 290,000 (20 to 490,000) | 200,000 (10 to 340,000) |
| CFR increase | | x 1.0 | Deaths | 180,000 (20 to 300,000) | 120,000 (7 to 200,000) | 80,000 (5 to 140,000) |
|  |  | x 1.5 | Deaths | 270,000 (30 to 460,000) | 180,000 (10 to 300,000) | 120,000 (7 to 210,000) |
|  |  | x 2.0 | Deaths | 400,000 (50 to 690,000) | 270,000 (15 to 450,000) | 180,000 (10 to 315,000) |
|  |  | x 1.0 | Peak deaths | 3,000 (1 to 7,000) | 2,000 (1 to 5,000) | 1,000 (1 to 3,000) |
|  |  | x 1.5 | Peak deaths | 4,000 (2 to 10,000) | 3,000 (2 to 7,000) | 2,000 (1 to 5,000) |
|  |  | x 2.0 | Peak deaths | 6,000 (5 to 15,000) | 5,000 (3 to 11,000) | 3,000 (2 to 8,000) |
|  | | | Peak non-ICU beds needed | 120,000 (10 to 330,000) | 80,000 (6 to 220,000) | 50,000 (5 to 150,000) |
|  |  |  | Peak ICU beds needed | 60,000 (7 to 180,000) | 40,000 (4 to 120,000) | 30,000 (3 to 80,000) |
| *Using global* $\boldsymbol{R}_{\boldsymbol{0}}$ *estimates* | | | | | | |
|  | | | Severe cases | 2,090,000 (1,030,000 to 2,670,000) | 970,000 (680,000 to 1,370,000) | 950,000 (470,000 to 1,220,000) |
|  |  |  | Critical cases | 890,000 (440,000 to 1,150,000) | 420,000 (290,000 to 590,000) | 410,000 (200,000 to 520,000) |
| CFR increase | x 1.0 | | Deaths | 370,000 (180,000 to 470,000) | 170,000 (120,000 to 250,000) | 17,000 (80,000 to 220,000) |
|  | x 1.5 | | Deaths | 560,000 (270,000 to 710,000) | 260,000 (180,000 to 370,000) | 250,000 (120,000 to 330,000) |
|  | x 2.0 | | Deaths | 840,000 (410,000 to 1,060,000) | 390,000 (270,000 to 555,000) | 380,000 (180,000 to 490,000) |
|  | x 1.0 | | Peak deaths | 13,000 (2,700 to 20,000) | 3,000 (2,000 to 7,000) | 5,000 (1,000 to 7,000) |
|  | x 1.5 | | Peak deaths | 20,000 (4,000 to 30,000) | 5,000 (3,000 to 10,000) | 7,000 (2,000 to 10,000) |
|  | x 2.0 | | Peak deaths | 30,000 (6,000 to 45,000) | 8,000 (5,000 to 15,000) | 11,000 (3,000 to 15,000) |
|  | | | Peak non-ICU beds needed | 480,000 (120,000 to 800,000) | 160,000 (80,000 to 310,000) | 220,000 (60,000 to 370,000) |
|  |  |  | Peak ICU beds needed | 250,000 (70,000 to 420,000) | 80,000 (40,000 to 170,000) | 120,000 (30,000 to 190,000) |
| Mauritius | | | | | | |
| *Using a distribution of* $\boldsymbol{R}_{\boldsymbol{t}}$ *estimates in African countries* | | | | | | |
|  | | | Severe cases | 30,000 (400 to 60,000) | 20,000 (200 to 40,000) | 10,000 (100 to 30,000) |
|  |  |  | Critical cases | 10,000 (100 to 30,000) | 10,000 (100 to 20,000) | 6,000 (40 to 10,000) |
| CFR increase | | x 1.0 | Deaths | 5,000 (60 to 13,000) | 4,000 (40 to 7,000) | 3,000 (10 to 5,000) |
|  |  | x 1.5 | Deaths | 8,000 (90 to 20,000) | 6,000 (60 to 10,000) | 4,000 (20 to 8,000) |
|  |  | x 2.0 | Deaths | 12,000 (100 to 30,000) | 9,000 (90 to 15,000) | 6,000 (30 to 12,000) |
|  |  | x 1.0 | Peak deaths | 100 (2 to 500) | 70 (1 to 300) | 50 (1 to 200) |
|  |  | x 1.5 | Peak deaths | 200 (3 to 700) | 100 (2 to 500) | 80 (2 to 300) |
|  |  | x 2.0 | Peak deaths | 300 (4 to 1,000) | 150 (3 to 800) | 120 (3 to 500) |
|  | | | Peak non-ICU beds needed | 4,000 (30 to 20,000) | 3,000 (20 to 10,000) | 2,000 (10 to 10,000) |
|  |  |  | Peak ICU beds needed | 2,000 (20 to 10,000) | 2,000 (10 to 8,000) | 1,000 (6 to 5,000) |
| *Using global* $\boldsymbol{R}_{\boldsymbol{0}}$ *estimates* | | | | | | |
|  | | | Severe cases | 50,000 (30,000 to 60,000) | 30,000 (20,000 to 40,000) | 20,000 (10,000 to 30,000) |
|  |  |  | Critical cases | 20,000 (10,000 to 30,000) | 10,000 (9,000 to 20,000) | 10,000 (6,000 to 10,000) |
| CFR increase | x 1.0 | | Deaths | 7,000 (5,000 to 13,000) | 5,000 (4,000 to 7,000) | 5,000 (3,000 to 5,000) |
|  | x 1.5 | | Deaths | 10,000 (8,000 to 20,000) | 7,000 (6,000 to 10,000) | 7,000 (4,000 to 8,000) |
|  | x 2.0 | | Deaths | 15,000 (12,000 to 30,000) | 11,000 (9,000 to 15,000) | 11,000 (6,000 to 12,000) |
|  | x 1.0 | | Peak deaths | 300 (70 to 500) | 100 (70 to 200) | 100 (50 to 300) |
|  | x 1.5 | | Peak deaths | 400 (100 to 700) | 200 (100 to 300) | 200 (70 to 400) |
|  | x 2.0 | | Peak deaths | 600 (100 to 1,100) | 300 (200 to 500) | 300 (100 to 600) |
|  | | | Peak non-ICU beds needed | 10,000 (4,000 to 20,000) | 5,000 (3,000 to 9,000) | 6,000 (2,000 to 10,000) |
|  |  |  | Peak ICU beds needed | 7,000 (2,000 to 10,000) | 3,000 (1,000 to 5,000) | 3,000 (900 to 5,000) |

# References

1. Wallinga J, Teunis P, Kretzschmar M. Using Data on Social Contacts to Estimate Age-specific Transmission Parameters for Respiratory-spread Infectious Agents. Am J Epidemiol. 2006;164:936–44.

2. Diekmann O, Heesterbeek JAP, Roberts MG. The construction of next-generation matrices for compartmental epidemic models. J R Soc Interface. 2010;7:873–85.

3. Davies NG, Kucharski AJ, Eggo RM, Gimma A, Edmunds WJ, Jombart T, et al. Effects of non-pharmaceutical interventions on COVID-19 cases, deaths, and demand for hospital services in the UK: a modelling study. Lancet Public Health. 2020;5:e375–85.

4. Li Q, Guan X, Wu P, Wang X, Zhou L, Tong Y, et al. Early Transmission Dynamics in Wuhan, China, of Novel Coronavirus–Infected Pneumonia. N Engl J Med. 2020;382:1199–207.

5. Bi Q, Wu Y, Mei S, Ye C, Zou X, Zhang Z, et al. Epidemiology and transmission of COVID-19 in 391 cases and 1286 of their close contacts in Shenzhen, China: a retrospective cohort study. Lancet Infect Dis. 2020;0. doi:10.1016/S1473-3099(20)30287-5.

6. Nishiura H, Linton NM, Akhmetzhanov AR. Serial interval of novel coronavirus (COVID-19) infections. Int J Infect Dis. 2020;93:284–6.

7. Liu Y, Centre for Mathematical Modelling of Infectious Diseases nCoV Working Group, Funk S, Flasche S. The contribution of pre-symptomatic infection to the transmission dynamics of COVID-2019. Wellcome Open Res. 2020;5:58.

8. Davies NG, Klepac P, Liu Y, Prem K, Jit M, Eggo RM. Age-dependent effects in the transmission and control of COVID-19 epidemics. Nat Med. 2020;:1–7.

9. Prem K, Van Zandvoort K, Klepac P, Eggo RM, Davies NG, CMMID COVID-19 working group, et al. Projecting contact matrices in 177 geographical regions: an update and comparison with empirical data for the COVID-19 era. https://cmmid.github.io/topics/covid19/synthetic-contact-matrices.html. Accessed 15 Aug 2020.

10. le Polain de Waroux O, Cohuet S, Ndazima D, Kucharski AJ, Juan-Giner A, Flasche S, et al. Characteristics of human encounters and social mixing patterns relevant to infectious diseases spread by close contact: a survey in Southwest Uganda. BMC Infect Dis. 2018;18:172.

11. Kiti MC, Kinyanjui TM, Koech DC, Munywoki PK, Medley GF, Nokes DJ. Quantifying Age-Related Rates of Social Contact Using Diaries in a Rural Coastal Population of Kenya. PLOS ONE. 2014;9:e104786.

12. Melegaro A, Del Fava E, Poletti P, Merler S, Nyamukapa C, Williams J, et al. Social Contact Structures and Time Use Patterns in the Manicaland Province of Zimbabwe. PLoS ONE. 2017;12. doi:10.1371/journal.pone.0170459.

13. Abbott S, Hellewell J, Thompson R, Sherratt K, Gibbs HP, Bosse NI, et al. Covid-19: Temporal variation in transmission during the COVID-19 outbreak. Covid-19. https://epiforecasts.io/covid/. Accessed 30 Aug 2020.

14. Jarvis CI, Van Zandvoort K, Gimma A, Prem K, Auzenbergs M, O’Reilly K, et al. Quantifying the impact of physical distance measures on the transmission of COVID-19 in the UK. BMC Med. 2020;18:124.

15. United Nations, Department of Economic and Social Affairs, Population Division. World Population Prospects - Population Division - United Nations. 2019. https://population.un.org/wpp/Download/Standard/Population/. Accessed 19 Apr 2020.

16. Linton NM, Kobayashi T, Yang Y, Hayashi K, Akhmetzhanov AR, Jung S, et al. Incubation Period and Other Epidemiological Characteristics of 2019 Novel Coronavirus Infections with Right Truncation: A Statistical Analysis of Publicly Available Case Data. J Clin Med. 2020;9:538.

17. Cao B, Wang Y, Wen D, Liu W, Wang J, Fan G, et al. A Trial of Lopinavir–Ritonavir in Adults Hospitalized with Severe Covid-19. N Engl J Med. 2020;:NEJMoa2001282.

18. National Health Service. Hospital Admitted Patient Care Activity 2018-19. NHS Digital. 2019. https://digital.nhs.uk/data-and-information/publications/statistical/hospital-admitted-patient-care-activity/2018-19. Accessed 19 Apr 2020.

19. Novel Coronavirus Pneumonia Emergency Response Epidemiology Team. [The epidemiological characteristics of an outbreak of 2019 novel coronavirus diseases (COVID-19) in China]. Zhonghua Liu Xing Bing Xue Za Zhi Zhonghua Liuxingbingxue Zazhi. 2020;41:145–51.

20. Russell TW, Hellewell J, Jarvis CI, Zandvoort K van, Abbott S, Ratnayake R, et al. Estimating the infection and case fatality ratio for coronavirus disease (COVID-19) using age-adjusted data from the outbreak on the Diamond Princess cruise ship, February 2020. Eurosurveillance. 2020;25:2000256.

21. Funk S. Socialmixr: Social Mixing Matrices for Infectious Disease Modelling. 2018. https://datacompass.lshtm.ac.uk/646/. Accessed 2 Jul 2020.
